# Targeting LIPA independent of its lipase activity is a therapeutic strategy in solid tumors via induction of endoplasmic reticulum stress

**DOI:** 10.1038/s43018-022-00389-8

**Published:** 2022-06-02

**Authors:** Xihui Liu, Suryavathi Viswanadhapalli, Shourya Kumar, Tae-Kyung Lee, Andrew Moore, Shihong Ma, Liping Chen, Michael Hsieh, Mengxing Li, Gangadhara R. Sareddy, Karla Parra, Eliot B. Blatt, Tanner C. Reese, Yuting Zhao, Annabel Chang, Hui Yan, Zhenming Xu, Uday P. Pratap, Zexuan Liu, Carlos M. Roggero, Zhenqiu Tan, Susan T. Weintraub, Yan Peng, Rajeshwar R. Tekmal, Carlos L. Arteaga, Jennifer Lippincott-Schwartz, Ratna K. Vadlamudi, Jung-Mo Ahn, Ganesh V. Raj

**Affiliations:** 1 Department of Urology, University of Texas Southwestern Medical Center at Dallas, Dallas, TX USA; 2 Department of Obstetrics and Gynecology, University of Texas Health Science Center at San Antonio, San Antonio, TX USA; 3 CDP program, Mays Cancer Center, University of Texas Health Science Center at San Antonio, San Antonio, TX USA; 4 Department of Chemistry and Biochemistry, University of Texas at Dallas, Richardson, TX USA; 5 Janelia Research Campus, Howard Hughes Medical Institute, Ashburn, VA USA; 6 Institute of Future Agriculture, Northwest A&F University, Yangling, China; 7 Department of Microbiology, Immunology and Molecular Genetics, The Joe R & Teresa Lozano Long School of Medicine, University of Texas Health Science Center at San Antonio, San Antonio, TX USA; 8 Fujian Provincial Key Laboratory of Neurodegenerative Disease and Aging Research, Institute of Neuroscience, Medical College, Xiamen University, Xiamen, China; 9 Department of Biochemistry and Structural Biology, University of Texas Health Science Center at San Antonio, San Antonio, TX USA; 10 Department of Pathology, University of Texas Southwestern Medical Center at Dallas, Dallas, TX USA; 11 Simmons Cancer Center, University of Texas Southwestern Medical Center at Dallas, Dallas, TX USA; 12 Audie L. Murphy Division, South Texas Veterans Health Care System, San Antonio, TX USA; 13 Department of Pharmacology, University of Texas Southwestern Medical Center at Dallas, Dallas, TX USA

**Keywords:** Drug development, Target identification, Cancer therapy, Cancer

## Abstract

Triple-negative breast cancer (TNBC) has a poor clinical outcome, due to a lack of actionable therapeutic targets. Herein we define lysosomal acid lipase A (LIPA) as a viable molecular target in TNBC and identify a stereospecific small molecule (ERX-41) that binds LIPA. ERX-41 induces endoplasmic reticulum (ER) stress resulting in cell death, and this effect is on target as evidenced by specific *LIPA* mutations providing resistance. Importantly, we demonstrate that ERX-41 activity is independent of LIPA lipase function but dependent on its ER localization. Mechanistically, ERX-41 binding of LIPA decreases expression of multiple ER-resident proteins involved in protein folding. This targeted vulnerability has a large therapeutic window, with no adverse effects either on normal mammary epithelial cells or in mice. Our study implicates a targeted strategy for solid tumors, including breast, brain, pancreatic and ovarian, whereby small, orally bioavailable molecules targeting LIPA block protein folding, induce ER stress and result in tumor cell death.

## Abstract (web summary)

Raj and colleagues show that ERX-41 inhibits lipase-independent functions of LIPA and induces ER stress in different tumor types, providing a therapeutic strategy in PDX models of pancreas, ovarian and triple-negative breast cancer.

## Main

Triple-negative breast cancers are negative for the expression of ER-α, PR or HER2, accounting for ~15% of new breast cancer (BC) diagnoses^1^. The absence of expression of these receptors means that effective agents targeting these receptors will have no therapeutic activity in TNBC. Currently, chemotherapy is the primary treatment option for the majority of patients with TNBC. TNBCs are aggressive tumors and have the highest mortality rate among all BC subtypes: 150,000 deaths worldwide were attributed to metastatic TNBC in 2018 alone^2,3^. There is thus an urgent and unmet need for effective targeted therapies in TNBC.

An ideal targeted therapy exploits specific vulnerabilities in cancer cells that are not seen in normal cells. However, TNBC represents a collection of multiple, biologically distinct subtypes categorized by their transcriptional profiles as basal-like (BL1, BL2), mesenchymal (M) or luminal androgen receptor (LAR) subtypes. Despite advances in tumor characterization, the molecular heterogeneity of TNBC and subtype-specific differences in immune cell composition and genetic and pharmacologic vulnerabilities limit the activity of individual targeted therapies.

We previously identified oligobenzamides D2 and ERX-11 that bind to androgen receptor^4^ and estrogen receptor (ER-α)^5^, respectively. During the process of lead optimization of these agents, we made the serendipitous discovery of a small molecule (ERX-41) with robust activity against multiple TNBC molecular subtypes. Herein we describe the identification, mechanism of action and molecular target of ERX-41 in TNBC and its applicability to other solid tumors.

## Results

### Derivation of ERX-41

**Fig. 1:**
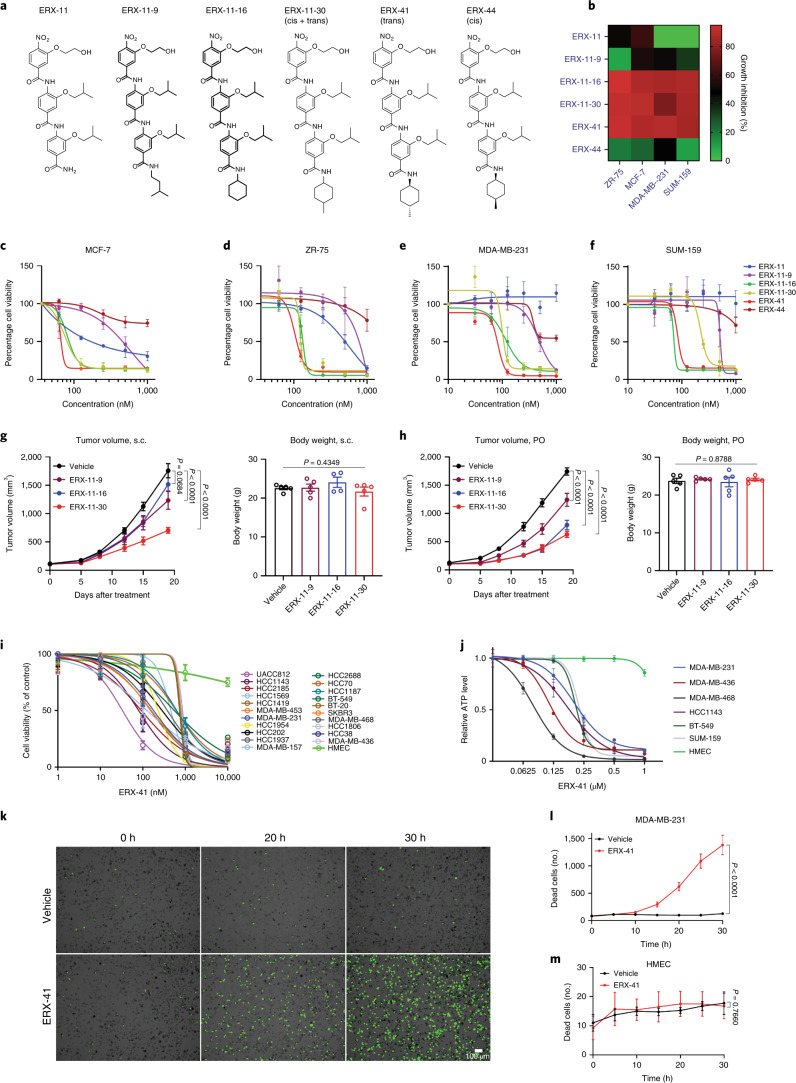
Derivation of ERX-41. **a**, Structures of ERX-11, ERX-11-9, ERX-11-16, ERX-11-30, ERX-41 and ERX-44. **b**, Heatmap showing ability of selected oligobenzamides to block proliferation of ER-α^+^ BC cells and TNBC cells in vitro (*n* = 3 replicates). **c**–**f**, MTT assays showing the effect of selected oligobenzamides on cell viability of MCF-7 (**c**), ZR-75 (**d**), MDA-MB-231 (**e**) and SUM-159 (**f**) cells in vitro. Data presented as mean ± s.e.m., *n* = 3 biologically independent samples. **g**,**h**, Following establishment of MDA-MB-231 TNBC xenografts in the mammary fat pad in vivo, daily administration of 10 mg kg^–1^ ERX-11-9, ERX-11-16, ERX-11-30 or vehicle control was initiated. Body weights are shown for each set, including for ERX-11-9, ERX-11-16 and ERX-11-30 administered s.c. (**g**) or PO (**h**). Data presented as mean ± s.e.m. For tumor volume data, *n* = 8 tumors per group; significance was determined by two-way ANOVA followed by Tukey’s multiple comparisons test. Adjusted *P* values for the last time points are shown. For body weight data, *n* = 5 mice per group; significance was determined by one-way ANOVA. **i**,**j**, Dose–response curve of ERX-41 in multiple TNBC cell lines using WST-1 assay (**i**) and CellTiter-Glo assay (**j**). Data presented as mean ± s.e.m.; *n* = 3 biologically independent samples. **k**, Time-lapsed images of live-cell imaging with SYTOX Green showing the effect of ERX-41-induced cell death in MDA-MB-231 at 0, 20 and 30 h after treatment with either vehicle or 1 μM ERX-41. **l**,**m**, quantification of the number of dead cells over time seen with live-cell imaging in MDA-MB-231 (**l**) and HMEC cells (**m**). Data presented as mean ± s.e.m.; *n* = 4 fields. Experiments were repeated twice independently, with similar results. Numerical source data for **b**–**j**,**l**,**m** are provided. Source data

**Extended Data Fig. 1:**
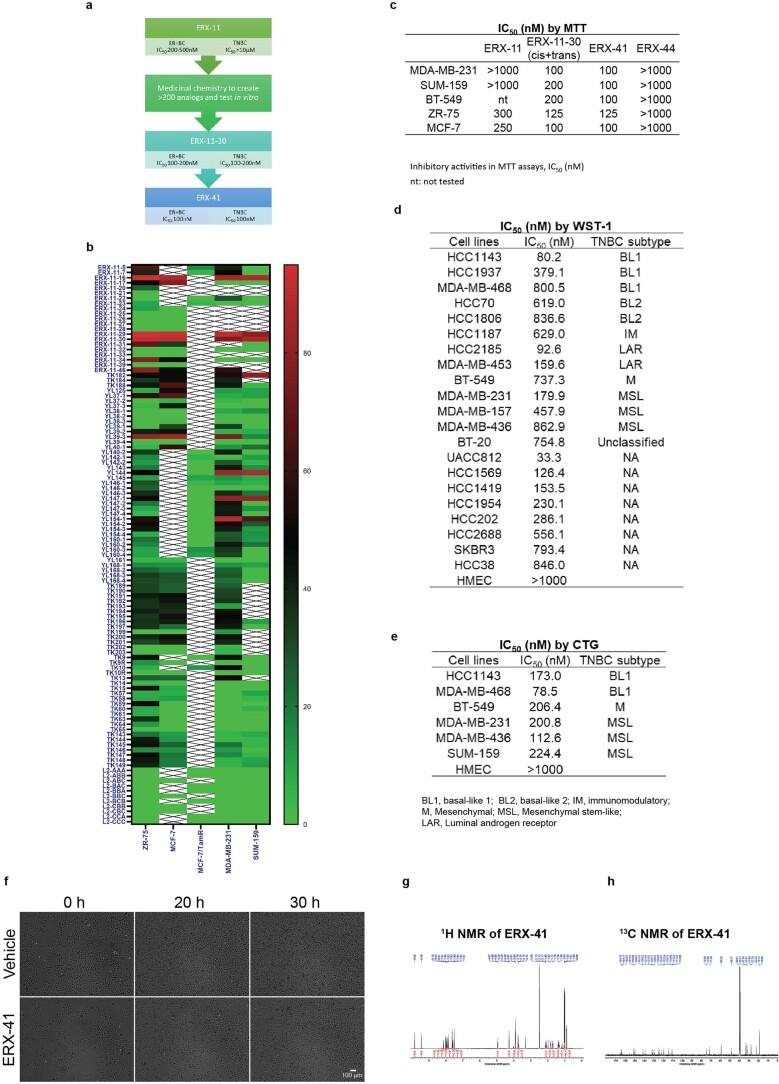
Identification of ERX-41. **a**: Schematic showing derivation of ERX-41 from ERX-11. Over 200 analogues of ERX-11 were synthesized and tested for antiproliferative activity using MTT assays in ZR-75, MCF-7 and MCF-7/Tamoxifen resistant ERα + cell lines and MDA-MB-231 and SUM-159 TNBC cells. The heatmap of the antiproliferative response of these cell lines to selected compounds is shown (**b**). The IC50 in nM as measured by MTT assays for four oligobenzamides in these cell lines is tabulated (**c**). The IC_50_ in nM for ERX-41 in multiple TNBC cells (along with their molecular subtype) is tabulated for the WST-1 assay (**d**) and the CTG assay (**e**). Time lapsed images of live cell imaging with SYTOX Green shows the effect of ERX-41 induced cell death in HMEC cells at 0 h, 20 h and 30 h after treatment (**f**). **g-h:** The ^1^H- and ^13^C-NMR spectra of the hit compound ERX-41 are shown (**g** and **h**, respectively). Source data

We designed and synthesized >200 analogs (Extended Data Fig. 1a) by exchanging the O-alkoxy substituents of D2 and ERX-11 with functional groups, including structurally diverse alkyl chains at the C-terminal carboxamide position. ERX-11 was potent (half-maximal inhibitory concentration (IC_50_ 200–500 nM) against ER-α^+^ BC, as shown for MCF-7 and ZR-75 (Extended Data Fig. 1a and Fig. 1a–d). Importantly, ERX-11 showed no activity (IC_50_ >10 μM) against SUM-159 and MDA-MB-231 TNBC cells (Extended Data Fig. 1a and Fig. 1b,e,f).

Here, we generated several analogs of ERX-11 to improve potency for BC. We found that some analogs (ERX-11-9, ERX-11-16 and ERX-11-30) (Fig. 1a–f and Extended Data Fig. 1b–e) also had potent activity against TNBC. We then tested both oral (PO) and subcutaneous (s.c.) administration of these analogs (to avoid potential issues with biodistribution) against xenografts in nude mice (Fig. 1). While all three analogs above decreased tumor growth rates and showed no toxicity (Fig. 1g,h), ERX-11-30 was the most potent in vivo (Fig. 1g,h).

Since ERX-11-30 is a racemic mixture, we synthesized its stereochemically defined isoforms ERX-11-41 (trans) and ERX-11-44 (cis) (shortened hereafter to ERX-41 and -44, respectively) to determine the stereochemical impact of the 4-methyl group (structures shown in Fig. 1a). Only *trans*-isoform ERX-41 showed activity against both TNBC and ER-α^+^ BC, with IC_50_ ~100–125 nM in MTT assays (Fig. 1b–f). In contrast, ERX-44 was not potent (Fig. 1b–f and Extended Data Fig. 1c). While ERX-41 (Extended Data Fig. 1c) was incrementally more potent than ERX-11 (IC_50_ 100–125 nM versus 200–500 nM) in ER-α^+^ BC, its potency in TNBC was enhanced (>100-fold) from IC_50_ >10 μM to 100 nM (Extended Data Fig. 1c). Based on these findings, we designated ERX-41 (Extended Data Fig. 1g,h) as a viable hit in TNBC.

In 21 cell lines, representing all molecular subtypes of TNBC, ERX-41 showed potent antiproliferative activity with IC_50_ <500 nM using WST-1, and <250 nM using CellTiter-Glo assays (Fig. 1i,j and Extended Data Fig. 1d,e). ERX-41 had no significant effect against normal human mammary epithelial cells (HMECs) at >1 μM with either WST-1, MTT or CellTiter-Glo assay (Fig. 1i,j and Extended Data Fig. 1d,e). Live-cell imaging studies indicated that ERX-41 significantly (>90%) induced TNBC cell death within 30 h of treatment compared with control (<1%) (Fig. 1k,l and Supplementary Video 1). In contrast, ERX-41 did not significantly induce HMEC cell death (Fig. 1m, Extended Data Fig. 1f and Supplementary Video 2).

**Fig. 2:**
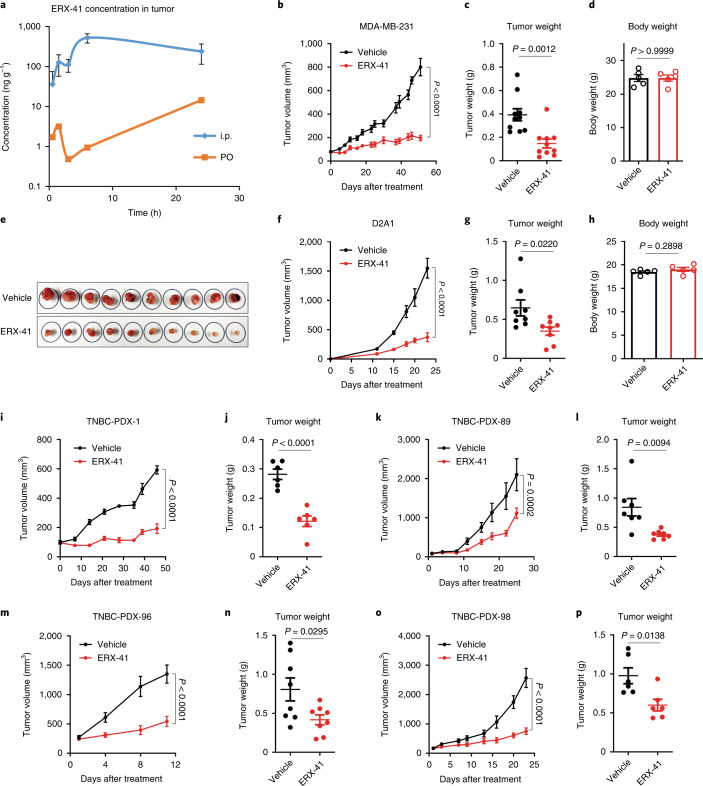
ERX-41 has potency against TNBC. **a**, Following establishment of s.c. MDA-MB-231 xenografts, 10 mg kg^–1^ single-dose ERX-41 was administered either PO or i.p. Tumor was harvested at 0, 0.5, 1.5, 3, 6 and 24 h after drug administration, and drug levels assayed by LC–MS/MS and graphed. Data presented as mean ± s.e.m.; i.p. group, *n* = 3 mice; PO group, *n* = 2 mice. **b**–**e**, Following establishment of MDA-MB-231 xenografts in mammary fat pad, daily administration of 10 mg kg^–1^ ERX-41 or vehicle control was initiated. Tumor volumes were measured using digital calipers and graphed (**b**). Tumor weights (**c**), body weights (**d**) and extirpated tumors (**e**) at study end are also shown. Data shown as mean ± s.e.m. For tumor volume, *n* = 10; significance was determined by two-way ANOVA with Bonferroni’s multiple comparisons test. Adjusted *P* values of last time points are shown. For tumor weight (*n* = 10) and body weight (*n* = 5), significance was determined by unpaired two-tailed Student’s *t*-test. **f**–**h**, After establishment of D2A1 syngeneic xenografts in mammary fat pad, daily administration of 10 mg kg^–1^ ERX-41 or vehicle control was initiated. Tumor volumes were graphed (**f**), along with tumor weights (**g**) and body weights (**h**). Data shown as mean ± s.e.m. For tumor volume data, *n* = 8; significance was determined by two-way ANOVA with Bonferroni’s multiple comparisons test. Adjusted *P* values of last time points are shown. For tumor weight (*n* = 8) and body weight (*n* = 5), significance was determined by unpaired two-tailed Student’s *t*-test. **i**–**p**, Effect of ERX-41 on growth (**i**,**k**,**m**,**o**) and tumor weight (**j**,**l**,**n**,**p**) in four TNBC-PDXs compared with vehicle. Data presented as mean ± s.e.m.; TNBC-PDX-1 (**i**,**j**), *n* = 6; TNBC-PDX-89 (**k**,**l**), *n* = 7; TNBC-PDX-96 (**m**,**n**), *n* = 8; TNBC-PDX-98 (**o**,**p**), *n* = 6 mice. For tumor volume data, significance was determined by two-way ANOVA with Bonferroni’s multiple comparisons test. Adjusted *P* values of last time points are shown. For tumor weight, significance was determined by unpaired two-tailed Student’s *t*-test. This experiment was performed once. Numerical source data for **a**–**d**,**f**–**p** are provided. Source data

Daily PO or intraperitoneal (i.p) administration of ERX-41 at doses up to 100 mg kg^–1^ showed no clear evidence of toxicity. Pharmacokinetic (PK) studies indicated that ERX-41 was orally bioavailable, with peak detectable plasma levels at 4 h after oral administration (10 mg kg^–1^ single dose). Additionally, ERX-41 was detectable within 1.5 h in established s.c. MDA-MB-231 xenografts after either PO or i.p. administration (Fig. 2a).

We show that ERX-41 (10 mg kg^–1^ d^–1^ PO) significantly inhibited the progression of established MDA-MB-231 xenografts in vivo (Fig. 2b). ERX-41 reduced tumor growth, as shown by extirpated tumor weights and sizes at the end of the study (Fig. 2c,e). Importantly, ERX-41 treatment did not show overt signs of toxicity, as evidenced by unchanged body weights of treated mice (Fig. 2d). ERX-41 significantly reduced growth of D2A1 xenografts in syngeneic mice without affecting body weight (Fig. 2f–h). We note that ERX-41 (10 mg kg^–1^ d^–1^ PO) also decreased growth of four distinct TNBC patient-derived xenografts (PDXs) in vivo (Fig. 2i,p) .

**Extended Data Fig. 2:**
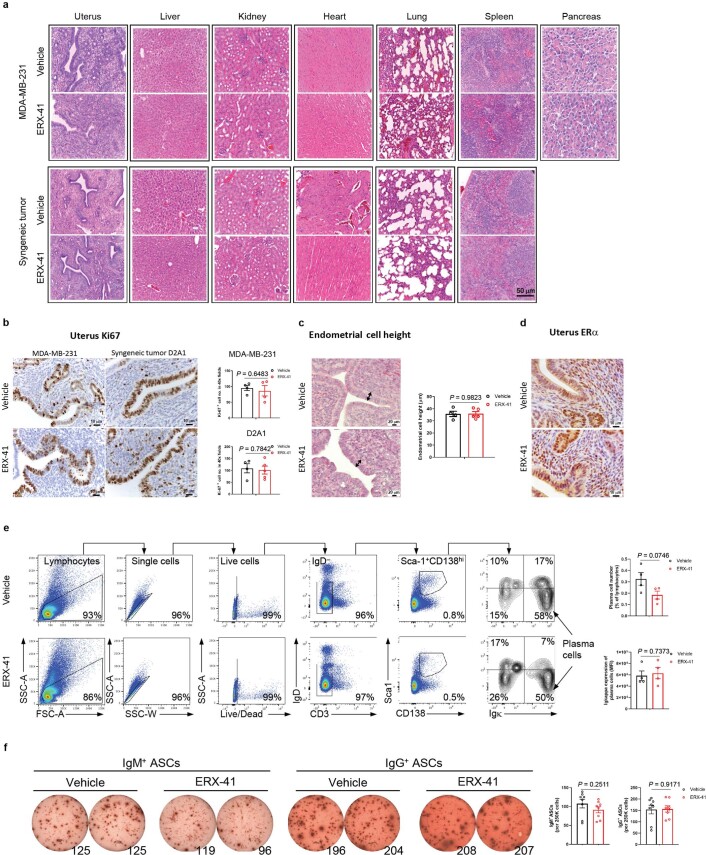
ERX-41 has no observed toxicity *in vivo*. Nude mice with MDA-MB-231 xenograft tumors were treated with either a vehicle or ERX-41 (10 mg/kg/day) for a duration of 52 days. Similarly, BALB/c mice with D2A1 xenograft tumors received a treatment of 20 mg/kg/day for 23 days. Following these treatments, histologic architecture in various organs of both the nude mice and BALB/c mice was assessed using H&E staining (**a**). The uteri in these mice were also evaluated for changes in their proliferation index (ki67 staining (**b**, with quantitation. Data are presented as mean ± SEM, *n* = 4 fields for all groups except the ‘ERX-41’ group in D2A1 mice, whose *n* = 5 fields. Significance was determined by unpaired two tailed Student’s *t*-test)), or endometrial cell height (**c**, with quantitation. Data are presented as mean ± SEM, *n* = 4 fields for ‘Vehicle’ group, *n* = 5 fields for ‘ERX-41’ group. Significance was determined by unpaired two tailed Student’s *t*-test), or ERα expression (**d**). **e-f:** Evaluation of the effect of daily administration of oral ERX-41 (10 mg/kg/day) for 10 days on the distribution of plasma cells in the bone marrow by flow cytometry (**e**, Data are presented as mean ± SEM, *n* = 4 mice. Significance was determined by unpaired two tailed Student’s *t*-test) and antibody-secreting cells (ASCs) that produces IgM or IgG in the bone marrow using ELISPOT analysis (**f**, Data are presented as mean ± SEM, *n* = 8 samples from 4 mice. Significance was determined by unpaired two tailed Student’s *t*-test) with quantitation. The experiments in **a and d** were done once. Numerical source data for **b, c, e and f** are provided. Source data

Histologic evaluation following ERX-41 treatment showed no significant changes in gross histology of multiple organs including heart, lung, spleen, liver, kidney, uterus and pancreas (Extended Data Fig. 2a). Similarly, in syngeneic mice, no significant histologic changes or immune infiltrates were noted in multiple organs, including spleen (Extended Data Fig. 2a), suggesting that ERX-41 is not immunogenic. In addition, ERX-41 did not affect the proliferation index (Extended Data Fig. 2b), endometrial cell height in the uterus (Extended Data Fig. 2c) or ER-α expression (Extended Data Fig. 2d): these findings are relevant, since the uterus is the organ most sensitive to estrogenic stimulus and antiestrogenic treatment. We then evaluated the effect of ERX-41 on bone marrow plasma cells by flow cytometry (Extended Data Fig. 2e) and ELISpot analysis (Extended Data Fig. 2f). Our analyses indicate that ERX-41 affected neither plasma cell numbers, immunoglobulin (Ig) Igκ expression in plasma cells nor number of IgM or IgG antibody-secreting cells (ASCs).

### ERX-41 induces ER stress

**Fig. 3:**
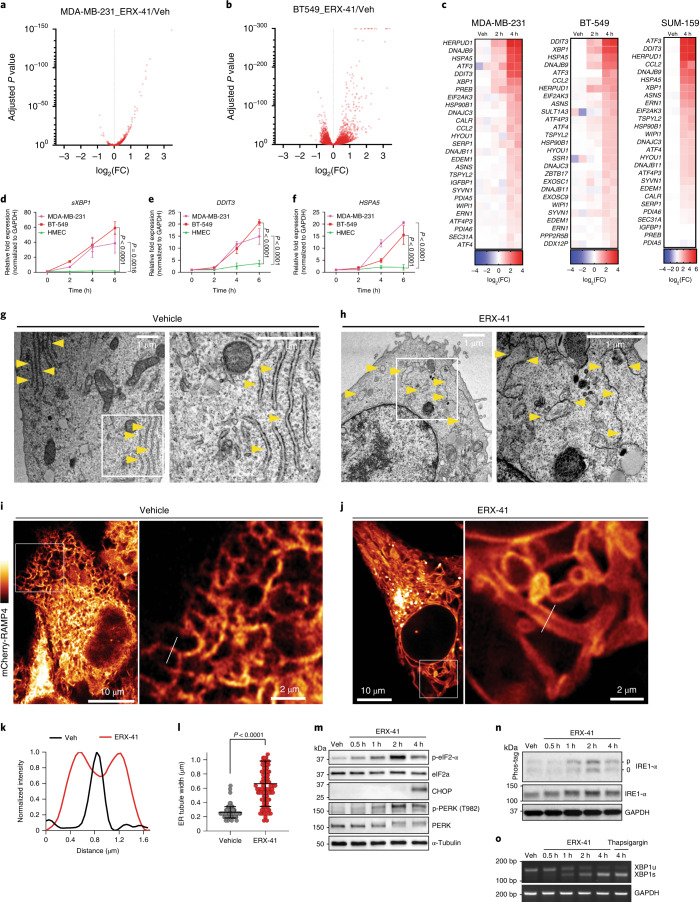
ERX-41 induces ER stress in TNBC. **a**,**b**, Volcano plots showing relative effect after 4 h of treatment by 1 μM ERX-41 vehicle (Veh) in MDA-MB-231 (**a**) and BT-549 (**b**) (*n* = 2 replicates). **c**, Heatmap of top ER stress/UPR genes in TNBC cells after 2 h and 4 h of 1 μM ERX-41. Fold change values in heatmap were calculated by normalization of FPKM values to those for vehicle. **d**–**f**, Effect over time of 1 μM ERX-41 on mRNA levels of ER stress genes *sXBP1* (**d**), *DDIT3* (**e**) and *HSPA5* (**f**) in MDA-MB-231, BT-549 and HMEC cells. Data presented as mean ± s.e.m.; *n* = 3 biologically independent samples. Significance was determined by two-way ANOVA with Tukey’s multiple comparisons test. Adjusted *P* values of last time points are shown. **g**,**h**, TEM of MDA-MB-231 cells showing effect of vehicle (**g**) and 1 μM ERX-41 (**h**) on subcellular structures at 4 h; ER denoted by yellow arrowheads. **i**–**l**, Airyscan imaging showing effect on SUM-159 cells stably expressing the ER membrane marker mCherry-RAMP4 before (**i**) and 2 h after 1 μM ERX-41 (**j**); right, zoomed-in images. Representative graph showing distribution of normalized tubule intensity (white lines in **i**,**j**) from vehicle and ERX-41-treated samples (**k**). Dilated ER is represented by two peaks, with signal between them indicating continuously dilated ER tubules. Histogram comparing distribution of ER tubule width (in μm) between untreated and treated samples (**l**). Data presented as mean ± s.d.; *n* = 102 measurements for vehicle, *n* = 104 measurements for ERX-41. Significance was determined by unpaired two-tailed Student’s *t*-test. **m**, Immunoblotting showing effect over time of 1 μM ERX-41 on UPR protein expression in SUM-159 cells. **n**, Immunoblot showing effect of 1 μM ERX-41 on IRE1-α expression in SUM-159 cells. Phos-tag gel (top) distinguishes between hypophosphorylated (0) and hyperphosphorylated (p) forms of IRE1-α. Total IRE1-α levels are shown (middle) with GAPDH (lower) control. **o**, RT–PCR showing effect over time of 1 μM ERX-41 on expression of unspliced (XBP1u) and spliced (XBP1s) XBP1 and *GAPDH* transcripts in SUM-159 cells. Effect of positive control (100 nM thapsigargin) is also shown. Experiments in **g**,**h**,**j**,**l**–**o** were repeated twice independently, with similar results. Numerical source data for **d**–**f**,**k**,**I** and uncropped blots for **m**–**o** are provided. bp, base pairs. Source data

**Extended Data Fig. 3:**
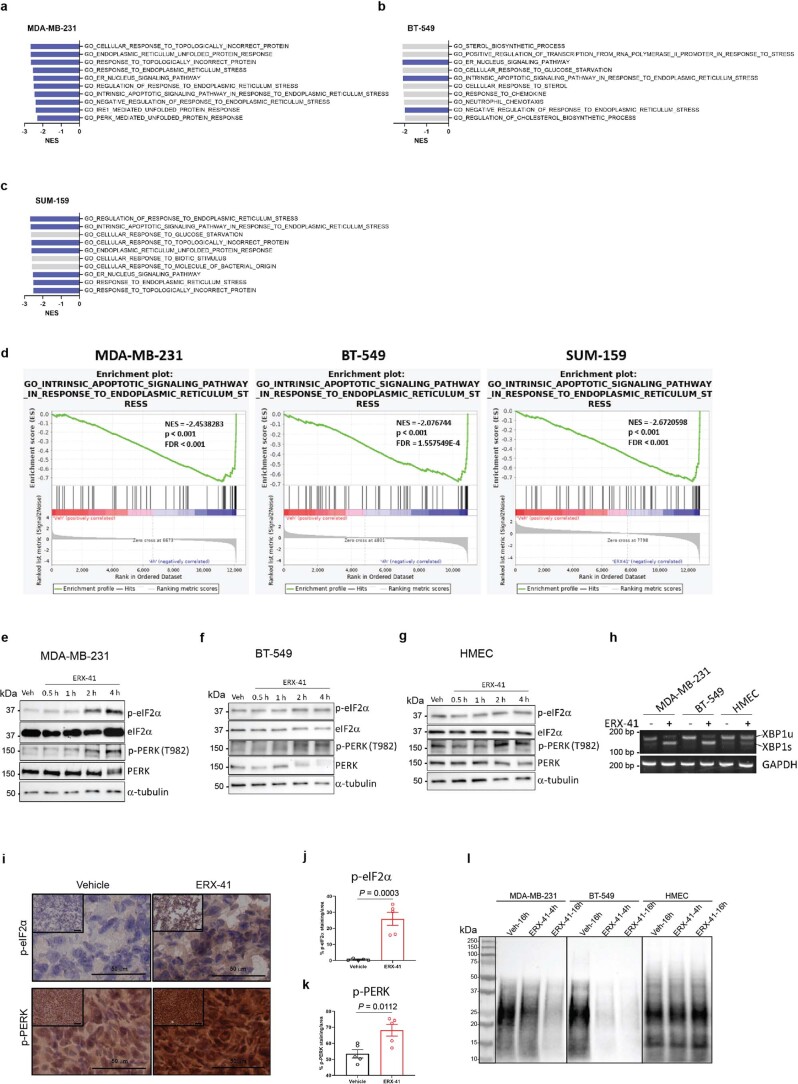
ERX-41 induces ER stress in TNBC. **a-d**: GSEA analysis of the RNA-sequencing data evaluating the treatment-enriched gene sets in three TNBC cell lines, MDA-MB-231 (**a**), BT-549 (**b**) and SUM-159 (**c**) following 4 h exposure to 1μM ERX-41. The top 10 gene sets (total ~15k C5 ontology gene sets) are shown for each cell line. ER- related gene sets are highlighted in blue. For all shown gene sets, the adjusted *P* value is <0.001 for all gene sets. GSEA showing correlation between ERX-41 treatment and induction of GO intrinsic apoptotic signaling pathway in response to ER stress in each of these three TNBC cell lines (**d**). **e-g**: Western blotting shows time course of effect of 1μM ERX-41 or 1μM ERX-11 on expression of UPR proteins in MDA-MB-231 (**e**), BT-549 (**f**) and HMEC cells (**g**). **h**: RT-PCR shows effect of 1μM ERX-41 on expression of unspliced (XBP1u) and spliced (XBP1s) XBP1 and *GAPDH* transcripts in MDA-MB-231, BT-549 and HMEC cells. **i-k**: Immunohistochemical evaluation of the effect of a single dose 10 mg/kg oral ERX-41 on the expression of ER stress markers in MDA-MB-231 TNBC tumor xenografts at 24 h after treatment (**i**), with quantitation of p-eIF2α (**j**) and p-PERK (**k**) in tumor tissue. Data are presented as mean ± SEM. *n* = 5 fields per group. Significance was determined by unpaired two tailed Student’s *t*-test **l:** Time course of effect of 1μM ERX-41 on global *de novo* protein synthesis in two TNBC (MDA-MB-231 and BT-549) and HMEC cells shown by western blots for puromycin labeled nascent proteins. The experiments in **e, f, g, h and l** were done twice independently with similar results. Numerical source data for **a, b, c, j, k** and uncropped images for **e, f, g, h, i** and **l** are provided. Source data

To understand the mechanism of action of ERX-41, we performed unbiased RNA sequencing (RNA-seq) studies in MDA-MB-231 and BT-549 cells (Fig. 3a,b). Gene set enrichment analyses (GSEA) revealed that the top pathways upregulated after 4 h of treatment with ERX-41 were related to induction of ER stress and compensatory unfolded protein response (UPR) pathways (Extended Data Fig. 3a–d). Heatmaps show induction of ER stress and UPR genes in TNBC cells (Fig. 3c). Quantitative PCR with reverse transcription (RT–qPCR) confirmed that canonical ER stress genes, heat shock protein 70 A family member 5 (HSPA5), DNA damage-inducible transcript 3 (DDIT3) and UPR stress sensor–spliced X-box-binding protein 1 (sXBP1) were dramatically upregulated (60–80-fold) in TNBC but not in HMEC cells following ERX-41 treatment (Fig. 3d,f).

We then performed ultrastructural studies using transmission electron microscopy (TEM). ERX-41 induced dramatic ER dilation within 4 h (Fig. 3g,h). Induction of ER dilation by TEM was noted in multiple TNBC but not in normal HMEC cells. Further ultrastructural validation was obtained by Airyscan super-resolution microscopy of live SUM-159 cells stably expressing the ER membrane marker mCherry-RAMP4 (Fig. 3i–l). In vehicle-treated cells, the peripheral ER network appeared as an intricate network of thin tubules connected by three-way junctions (Fig. 3i), and as a single peak on distance histograms (Fig. 3k). Within 2 h of ERX-41 treatment we observed marked disorganization of the peripheral ER network, characterized by striking elongation and dilation of individual tubules (Fig. 3j,k) (mean ± s.d. tubule width at 0 h, 260 ± 80 nm; at 2 h, 660 ± 320 nm; *n* (0/2 h) = 102/104 tubules, *P* < 0.0001) (Fig. 3l). Importantly, since the width of normal ER tubules is ~50–100 nm and thus below the resolving power of Airyscan microscopy (100–200 nm), we expect that the true effect of ERX-41 on tubule width is probably even greater. These data confirm that ERX-41 induces ER stress in TNBC.

We then biochemically confirmed that ERX-41 induces ER stress and downstream UPR pathways via induction of phosphorylated protein kinase R-like ER kinase (p-PERK) and phosphorylated eukaryotic translation initiation factor 2 subunit 1 (p-eIF2-α), and by expression of CCAAT-enhancer-binding homologous protein (CHOP) and phosphorylated inositol-requiring enzyme 1-α (IRE1-α) in TNBC (Fig. 3m,n and Extended Data Fig. 3e,f), but not in HMEC cells (Extended Data Fig. 3g). At the RNA level, ERX-41 induced spliced XBP1 in SUM-159 (Fig. 3o), MDA-MB-231 and BT-549 cells but not in HMEC cells (Extended Data Fig. 3h)

We confirmed that a single dose of ERX-41 induces ER stress in vivo, as evidenced by enhanced p-PERK and p-eIF2-α staining in TNBC xenografts within 24 h of treatment (Extended Data Fig. 3i–k). Since our PK studies showed that treated tumor tissues have detectable levels of ERX-41 at 24 h (Fig. 2a), these data indicate that ERX-41 selectively induces ER stress.

The functional consequence of ERX-41 induction of ER stress is that global de novo protein synthesis in TNBC, but not in HMEC, cells is blocked by ERX-41, as shown by immunoblots for puromycin-labeled nascent proteins (Extended Data Fig. 3l). These data suggest that ERX-41 can induce uncompensated ER stress, resulting in shutdown of ER function and protein synthesis leading to cell death.

### Molecular target of ERX-41 is *LIPA*

**Extended Data Fig. 4:**
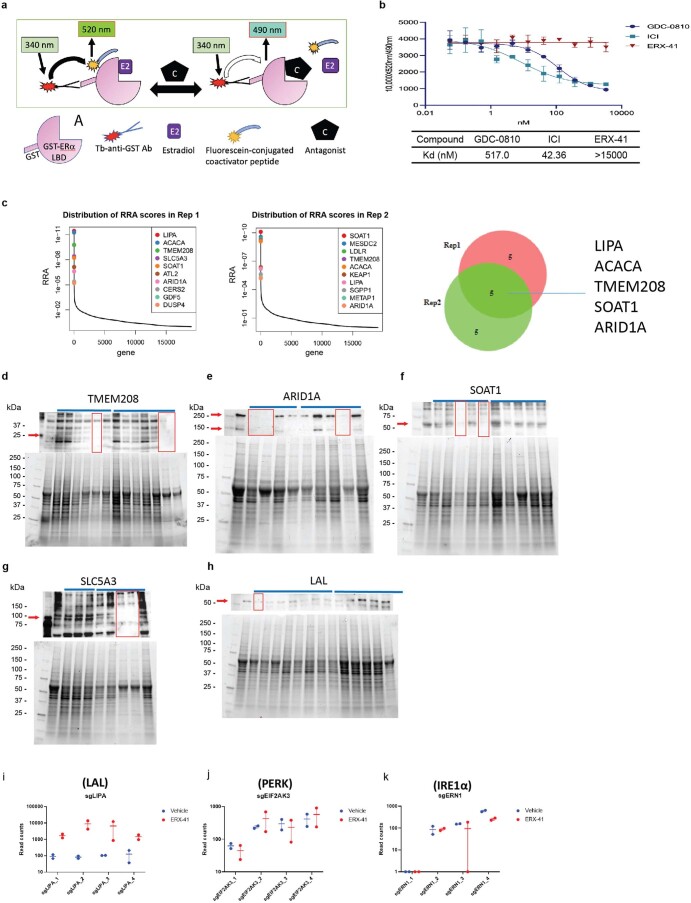
Molecular target of ERX-41 is *LIPA*. **a-b:** Schematic representation of LanthaScreen TR-FRET ER Alpha Coactivator assay (**a**). Tb-anti-GST antibody indirectly labels ERα by binding to the GST tag. Binding of the agonist to ERα causes a conformational change, leading to an increase in the affinity of ERα for a coactivator LXXLL peptide. The close proximity of the fluorescently labeled coactivator peptide to the terbium-labeled antibody results in an increase in the TR-FRET signal. Representative data from TR-FRET ERα Coactivator assay run in antagonist mode. Results after 2-hour incubation for ERX-41, Fulvestrant (ICI) and a SERD (GDC-0810) are graphed and the kd values tabulated below (**b**). Data are presented as mean ± SEM, *n* = 3 biologically independent samples. **c**: Correlation between two biologic replicates of the CRISPR screen in MDA-MB-231 cells is graphed with the overlap depicted using Venn diagram. The top 10 genes in both replicates are highlighted. **d-h**: Western blotting shows the identification of clones of MDA-MB-231 (outlined in red) showing protein level knockout of expression of *TMEM208* (**d**), *ARID1A* (**e**), *SOAT1* (**f**) *SLC5A3* (**g**), and *LIPA* (**g**). Red arrows indicate the predicted MW of each target. **i-k**: The enrichment of four individual CRISPR guide RNAs on the responsiveness of MDA-MB-231 cells to vehicle or 1μM ERX-41 treatment, as shown for *LIPA* (gene of LAL) (**i**), *EIF2AK3* (gene for PERK) (**j**) and ERN1 (gene for IRE1α) (**k**). Data are presented as mean ± SEM, *n* = 2 biological replicates. The experiments in **d, e, f, g and h** were done once. Numerical source data for **b, c, I, j, k** and uncropped blots for **d, e, f, g, h** are provided. Source data

Since ERX-41 was derived from ERX-11 (which targets ER-α), we established that ERX-41 did not interact with ER-α. Using a time-resolved measurement with fluorescence resonance energy transfer (TR–FRET) assay, we demonstrated that ERX-41 does not interact with the ER-α ligand-binding domain (LBD), unlike fulvestrant and selective ER-α degraders such as GDC-0810, tamoxifen and ERX-11 (data shown for fulvestrant and GDC-0810) (Extended Data Fig. 4a,b). These data suggest that ERX-11 and ERX-41 have distinct molecular targets.

**Fig. 4:**
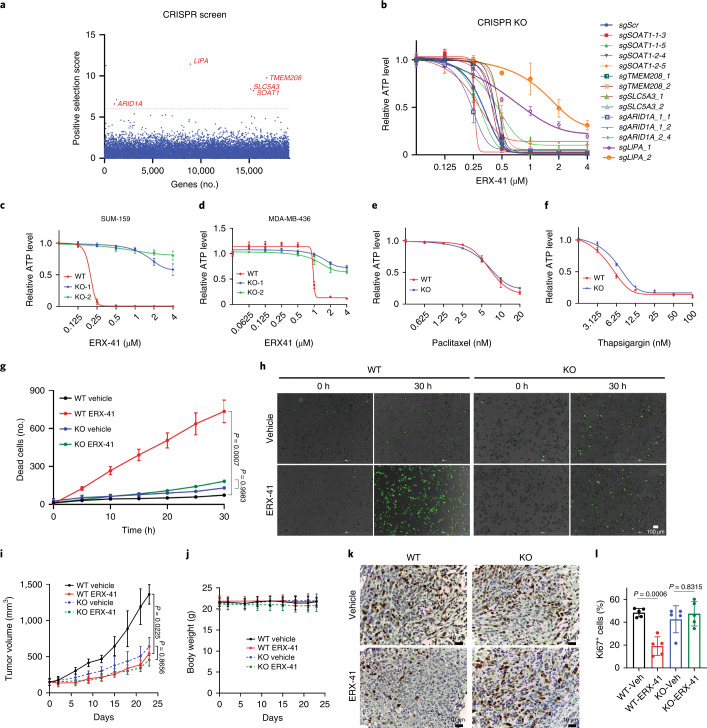
Molecular target of ERX-41 is *LIPA*. **a**, Visualization of CRISPR–Cas9 screen in MDA-MB-231 cells showing genes associated with resistance to ERX-41; the top five genes are highlighted. **b**, KO clones of *LIPA*, *SLC5A3*, *TMEM208*, *SOAT1* and *ARID1A* in MDA-MB-231 cells were evaluated for dose response to ERX-41 using CellTiter-Glo assays. Data presented as mean ± s.e.m., *n* = 3 biologically independent samples. Each gene was knocked out by two different sgRNAs. **c**,**d**, Effect of knockout of *LIPA* in SUM-159 (**c**) and MDA-MB-436 (**d**) on dose response to ERX-41 using CellTiter-Glo assays in vitro. Data presented as mean ± s.e.m., *n* = 3 biologically independent samples. **e**,**f**, Dose responses of parental SUM-159 and clones with *LIPA* KO to paclitaxel (**e**) and thapsigargin (**f**). Data presented as mean ± s.e.m., *n* = 3 biologically independent samples. **g**,**h**, Live-cell imaging studies with SYTOX Green showing ability of 1 μM ERX-41 to induce cell death in parental SUM-159 and SUM-159 clones with *LIPA* KO, with quantitation (**g**) and time-lapsed photomicrographs at 0 h and 30 h (**h**). Data presented as mean ± s.d., *n* = 4 fields per group. Significance was determined by one-way ANOVA with Tukey’s multiple comparisons test. Adjusted *P* values of last time points are shown. **i**–**l**, SCID mice were implanted with either parental SUM-159 or SUM-159 clones with *LIPA* KO and tumors allowed to establish (150 mm^3^). Following daily i.p. administration of vehicle or 10 mg kg^–1^ ERX-41, tumor size was measured and graphed (**i**), with mice body weights (**j**). Data presented as mean ± SEM, *n* = 5 mice per group. Significance was determined by one-way ANOVA with Tukey’s multiple comparisons test. Adjusted *P* values of last time points are shown. Xenograft tumors were harvested and processed for IHC with Ki67. Representative IHC staining is shown (**k**) and proliferative indices for each tumor are quantitated and graphed (**l**). Data presented as mean ± s.d., *n* = 5 per group. Significance was determined by one-way ANOVA with Tukey’s multiple comparisons test. Adjusted *P* values are shown. Experiment shown in **h** was done twice independently, with similar results, while that shown in **i**,**j** was done once. Numerical source data for **a**–**g**,**i**,**j**,**I** are provided. Source data

To identify the molecular target of ERX-41, we performed an unbiased CRISPR–Cas9 knockout (KO) screen in MDA-MB-231 cells. There was significant concordance between two independent experiments of the screen performed at two distinct concentrations of ERX-41, and the top six genes were subjected to a secondary screen in MDA-MB-231 cells (Fig. 4a and Extended Data Fig. 4c). TNBC cell lines with KO of five of the top six genes—*LIPA*, *SLC5A3*, *TMEM208*, *SOAT1 and ARID1A*—were generated and evaluated for response to ERX-41 (Extended Data Fig. 4d–h). Of these, KO of *LIPA* alone (which encodes lysosomal acid lipase (LAL)) was able consistently to abrogate cytotoxic response to ERX-41 (Fig. 4b and Extended Data Fig. 4i). Knockout of individual ER stress/UPR genes such as *PERK* and *IRE1-α* did not affect the ability of ERX-41 to cause cell death, suggesting that multiple ER stress pathways had been activated (Extended Data Fig. 4j,k). While these data do not rule out a role for other identified ERX-41 targets, we were able to show that KO of *LIPA* in SUM-159 and MDA-MB-436 was able to alter the response to ERX-41 (Fig. 4c,d) in CellTiter-Glo assays in vitro. The altered response of SUM-159 clones with *LIPA* KO was specific for ERX-41, as shown by similar responses of SUM-159 parental and *LIPA* KO clones to thapsigargin or paclitaxel (Fig. 4e,f).

Live-cell imaging studies confirmed that KO of *LIPA* altered the response of SUM-159 cells to ERX-41 with a dramatic decrease in cell death (Fig. 4g,h and Supplementary Video 3). We then confirmed that xenografts of SUM-159 with *LIPA* KO did not respond to i.p. administration of ERX-41, in contrast to parental SUM-159 xenografts which responded significantly to ERX-41 in vivo (Fig. 4i,j). Importantly, evaluation of proliferative indices of xenograft tumors showed a decrease in Ki67 staining in parental SUM-159 xenografts but not in SUM-159 *LIPA* KO xenografts (Fig. 4k,l).

**Fig. 5:**
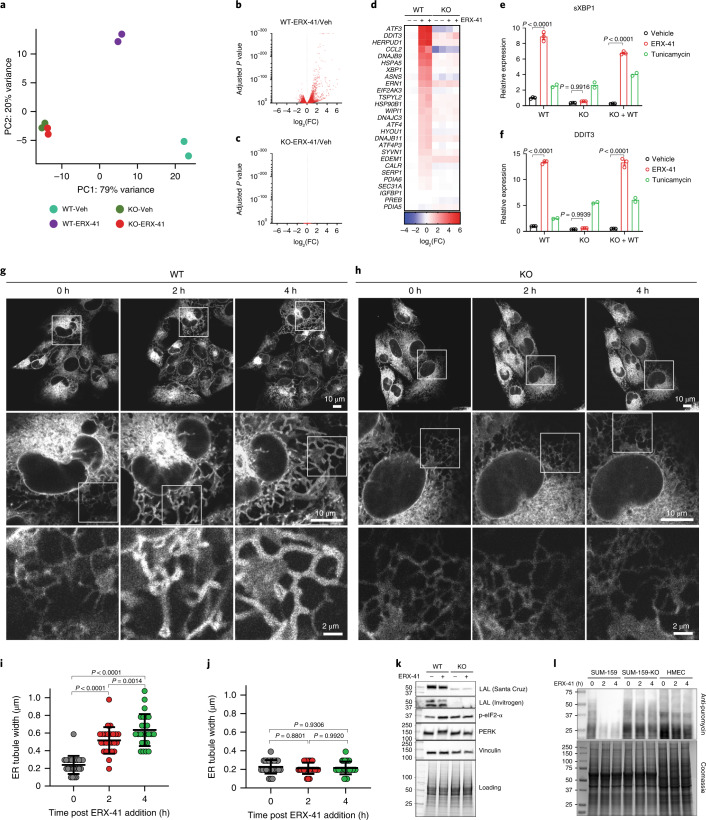
*LIPA* is the target of ERX-41. **a**–**d**, Following treatment with vehicle or 1 μM ERX-41 for 4 h in parental SUM-159 and SUM-159 clones with *LIPA* KO, unbiased RNA-seq was performed. PC analyses of sequencing data (**a**) and volcano plots in parental (**b**) and *LIPA* KO cells (**c**), and heatmap of canonical genes involved in ER stress and UPR response (**d**), are shown. Fold change values in **d** were calculated by normalization of FPKM values to averaged FPKM values of WT-vehicle condition. **e**,**f**, RT–qPCR evaluation of expression of sXBP1 (**e**) and DDIT3 (**f**) in parental SUM-159 cells and SUM-159 cells with *LIPA* KO. Data presented as mean ± s.e.m.; for vehicle and ERX-41, *n* = 3 biologically independent samples; for tunicamycin, *n* = 2 biologically independent samples. Significance was determined by two-way ANOVA with Tukey’s multiple comparisons test. Adjusted *P* values of last time points are shown. **g**–**j**, Live-cell confocal microscopy showing serial effect of 1 μM ERX-41 on morphology of SUM-159 cells stably expressing ER membrane marker mCherry-RAMP4 at 0, 2 and 4 h (**g**). Serial magnification of same field of view, with field view (top), single-cell view (middle) and detail (bottom). The same experiment was repeated in SUM-159 cells with *LIPA* KO (**h**). **i**,**j**, Histograms showing the effect of 1 μM ERX-41 on ER tubule width in parental (**i**) and *LIPA* KO cells (**j**). Data presented as mean ± s.d.; *n* = 25 measurements per condition. Significance was determined by two-way ANOVA with Tukey’s multiple comparisons test. Adjusted *P* values of last time points are shown. **k**, Immunoblot showing the effect of 1 μM ERX-41 on expression of LAL and ER stress proteins p-eIF2-α and PERK in parental SUM-159 and *LIPA* KO clone**. l**, Time course of effect of 1 μM ERX-41 on global de novo protein synthesis in parental SUM-159 and *LIPA* KO clone, shown by immunoblots for puromycin-labeled nascent proteins. Effect on HMEC cells is shown for comparison. Experiments shown in **g**,**h**,**k**,**l** were performed twice independently, with similar results. Numerical source data for **b**–**f**,**i**,**j** and uncropped blots for **k**,**l** are provided. Source data

**Extended Data Fig. 5:**
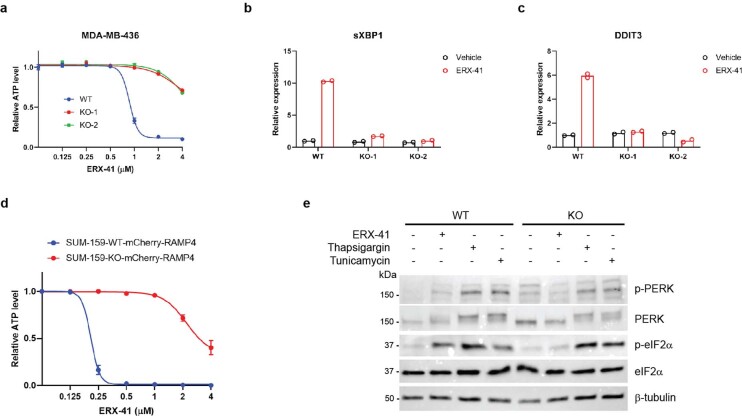
*LIPA* is the target of ERX-41. **a-c**: Dose response curve of the effect of ERX-41 on the proliferation of parental MDA-MB-436 cells and two MDA-MB-436 clones with *LIPA* KO by CellTiter-Glo assays (**a**). RT-qPCR evaluation of the effect of 1μM ERX-41 on expression of sXBP1 (**b**) and DDIT3 (**c**) in parental MDA-MB-436 cells and MDA-MB-436 clones with *LIPA* KO is graphed. *n* = 2 biologically independent samples. **d:** Effect of ERX-41 on the proliferation of parental SUM-159 cells and SUM-159 cells with *LIPA* KO stably expressing the ER membrane marker mCherry-RAMP4 as measured by CellTiter-Glo. Data are presented as mean ± SEM. *n* = 3 biologically independent samples. **e:** Western blot shows effect of 1μM ERX-41 or thapsigargin or tunicamycin treatment on expression of UPR proteins in parental SUM-159 and SUM-159 cells with *LIPA* KO. The experiments **e** were done twice independently with similar results. Numerical source data for **a, b, c, d** and uncropped blots for **e** are provided. Source data

To ascertain that *LIPA* is associated with ERX-41-induced ER stress, we performed unbiased RNA-seq studies with and without ERX-41 in parental and *LIPA* KO SUM-159 cells. Principal component (PC) analyses showed that gene expression profiles in these cells tend to cluster independently (Fig. 5a). While volcano plots showed significant alteration in gene expression in parental SUM-159 cells (Fig. 5b), there were no genes significantly altered in *LIPA* KO SUM-159 cells following ERX-41 treatment (Fig. 5c). Evaluation of canonical genes involved in ER stress and UPR response shows induction of these genes by ERX-41 in parental SUM-159 cells but not in cells with *LIPA* KO (Fig. 5d). These findings were confirmed by RT–qPCR, showing that ERX-41 induces expression of UPR genes *sXBP1* and *DDIT3* in parental SUM-159 cells but not in SUM-159 cells with *LIPA* KO (Fig. 5e,f). These data were validated in MDA-MB-436 cells with *LIPA* KO (Extended Data Fig. 5a–c). Reconstitution of WT-*LIPA* (KO + WT) in SUM-159 cells with *LIPA* KO restored the inducibility of sXBP and DDIT3 by ERX-41 (Fig. 5e,f).

Ultrastructural studies using live-cell confocal microscopy show that *LIPA* KO abrogated ER morphological changes at 2 and 4 h after treatment (Fig. 5g,h). While changes in ER luminal diameter are more readily apparent with Airyscan imaging, live-cell confocal microscopy allows serial evaluation over time. As noted with Airyscan microscopy, increases in ER tubule diameter from ~200 to ~500 nm at 2 h and to ~620 nm at 4 h were apparent in parental SUM-159 cells after ERX-41 treatment (Fig. 5g,i). In contrast, no significant changes in ER tubule diameter were noted in *LIPA* KO SUM-159 cells after ERX-41 treatment (Fig. 5h,j). Importantly, SUM-159 cells with stably transduced mCherry-RAMP4 responded to ERX-41 as did parental SUM-159, with IC_50_ ~200 nM in WT cells and ~4 μM in *LIPA* KO cells (Extended Data Fig. 5d).

Immunoblotting revealed that ERX-41 activates PERK (noted by upshifting of the PERK band) and induced p-eIF2-α in parental SUM-159 cells, but not in SUM-159 cells, with *LIPA* KO (Fig. 5k). Importantly, inducibility of UPR genes in SUM-159 cells with *LIPA* KO with the known ER stress inducer thapsigargin (inhibitor of ER Ca^2+^ ATPase) or tunicamycin (blocks N-linked glycosylation) was preserved (Fig. 5e,f and Extended Data Fig. 5e). Consequently, ERX-41 blocked de novo protein synthesis in parental SUM-159 cells, but not in SUM-159 cells, with *LIPA* KO or in HMEC cells (Fig. 5l).

### *LIPA* subcellular localization in the ER

**Extended Data Fig. 6:**
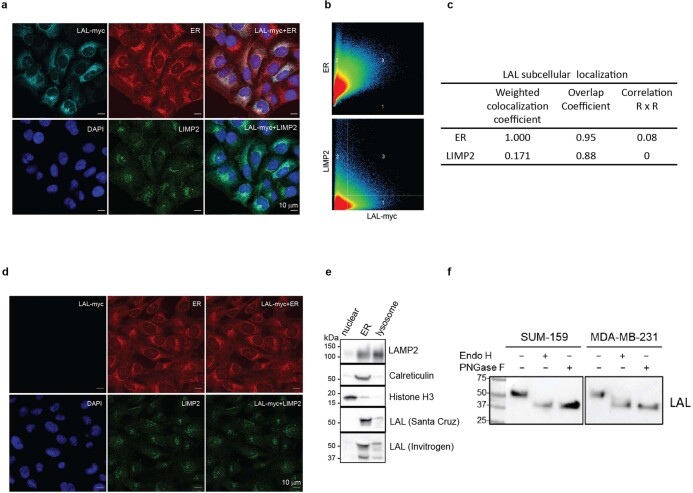
LAL subcellular localization in the ER. **a-d:** Immunofluorescence shows colocalization of myc-tagged LAL protein with either ER (ER tracker) or lysosomal markers (LIMP2) in SUM-159 cells with overexpressed LAL-myc (**a**) and in parental SUM-159 cells without LAL-myc (**d**). Colocalization between expression parameters in the LAL-myc expressing cells are graphically correlated with quadrant quantification (**b**) and weighted colocalization factor calculations are tabulated (**c**). **e:** Western blot shows expression of LAL (using two different antibodies) along with ER (calreticulin), lysosomal (LAMP2) and nuclear (Histone H3) markers in different subcellular fractions of SUM-159 cells. **f**: Sensitivity of endogenous LAL from SUM-159 and MDA-MB-231 cells to glycosidase cleavage with endoglycosidase H (endo H) or Peptide-N-Glycosidase F (PNGase F). The experiments in **a, d, e and f** were done twice independently with similar results. (**f**). Uncropped blots for **e** and **f** are provided. Source data

The known function of LAL protein as a lysosomal acid lipase relates to its subcellular lysosomal localization. The ability of ERX-41 to induce ER stress prompted evaluation of LAL subcellular localization in TNBC using coimmunofluorescence with both ER and lysosomal markers in SUM-159 cells with overexpressed myc-tagged LAL (Extended Data Fig. 6a). We noted high colocalization of LAL protein with the ER tracker, with a weighted colocalization factor of 1.0 (Extended Data Fig. 6b,c). In contrast, LAL protein poorly colocalized with lysosomal marker LIMP2, with a weighted colocalization factor of 0.17 (Extended Data Fig. 6a–c). The specificity of myc antibody was confirmed by negative staining in parental SUM-159 cells, which have no myc-tagged LAL expression (Extended Data Fig. 6d). These data were supported by biochemical evaluation of subcellular fractions of SUM-159 cells (Extended Data Fig. 6e), which confirmed enrichment of LAL protein in the ER-enriched subcellular fraction. In addition, the sensitivity of glycosylated LIPA to endoglycosidase H (Endo H) and peptide-*N*-glycosidase F (PNGase F) cleavage supports its ER localization^6^ (Extended Data Fig. 6f). If glycosylation occurred in the Golgi, then glycosylated *LIPA* would be sensitive to PNGase F but not to Endo H cleavage. Since the role of *LIPA* in the ER has not been previously described, these data support our central finding that *LIPA* plays a role in ER homeostasis. Taken together, these data suggest a previously uncharacterized function for *LIPA* in the ER that is targeted by ERX-41 and that induces ER stress in TNBC.

### *LIPA* as a target in TNBC and solid tumors

**Fig. 6:**
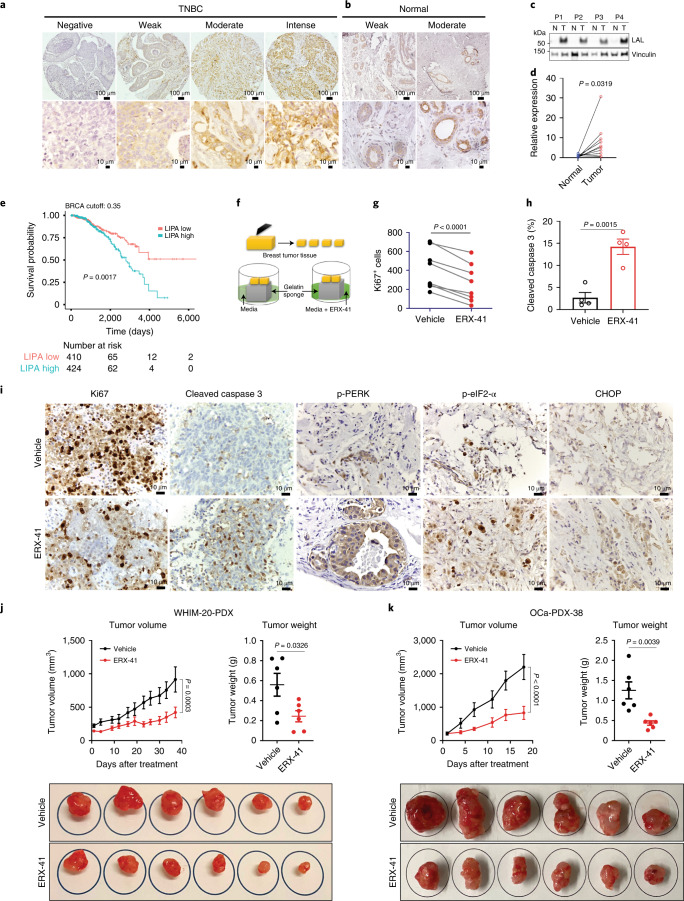
*LIPA* is a viable molecular target in TNBC and other cancers. **a**–**c**, Samples from 51 patients with TNBC (**a**) and from 20 individuals with normal breast tissue (**b**) were evaluated for LAL expression using TNBC tissue microarray, with representative images. **c**, Immunoblots showing expression of LAL protein in matched tumor (T) and adjacent normal tissue (N) from four patients with TNBC (P1–4). **d**, Quantitation of LAL protein expression from matched tumor and adjacent normal tissues; *n* = 10 per group. **e**, Top, Kaplan–Meier curves showing correlation between expression levels of LAL and survival outcomes using a TCGA dataset; bottom, numerical tabulation. **f**–**h**, Following surgical extirpation, primary TNBC tumors were cultured ex vivo with either vehicle or 1 μM ERX-41, as shown by the schematic (**f**). Effect on proliferation index (Ki67) (**g**) and apoptosis marker (cleaved caspase 3, **h**), *n* = 8 patients per group. Significance was determined by paired two-tailed Student’s *t*-test). Data presented as mean ± s.e.m. **i**, Representative IHC images of the effect of ERX-41 on Ki67, cleaved caspase 3 and UPR proteins are shown. **j**, Following establishment of ER-α^+^ WHIM-20 BC PDX in mammary fat pad in NSG mice, daily administration of 10 mg kg^–1^ ERX-41 or vehicle control was initiated. Tumor volume was measured using digital calipers and graphed. Tumor weights and extirpated tumors at end of study are shown. **k**, Following establishment of s.c. ovarian cancer PDX (OCa-PDX-38) tumors in NSG mice, daily administration of 10 mg kg^–1^ ERX-41 or vehicle control was initiated. Tumor volume was measured using digital calipers and graphed. Tumor weights and extirpated tumors at end of study are shown. **j**,**k**, Data presented as mean ± s.e.m. For tumor volume data, *n* = 6 tumors; significance was determined by two-way ANOVA with Bonferroni’s multiple comparisons test. Adjusted *P* values of the last time points are shown. For tumor weight data, *n* = 6 tumors. Significance was determined by unpaired two-tailed Student’s *t*-test. Experiments shown in **a**,**b**,**i** were performed once. Numerical source data for **d**,**g**–**i**,**k** and uncropped blots for **c** are provided. BRCA, breast invasive carcinoma. Source data

**Extended Data Fig. 7:**
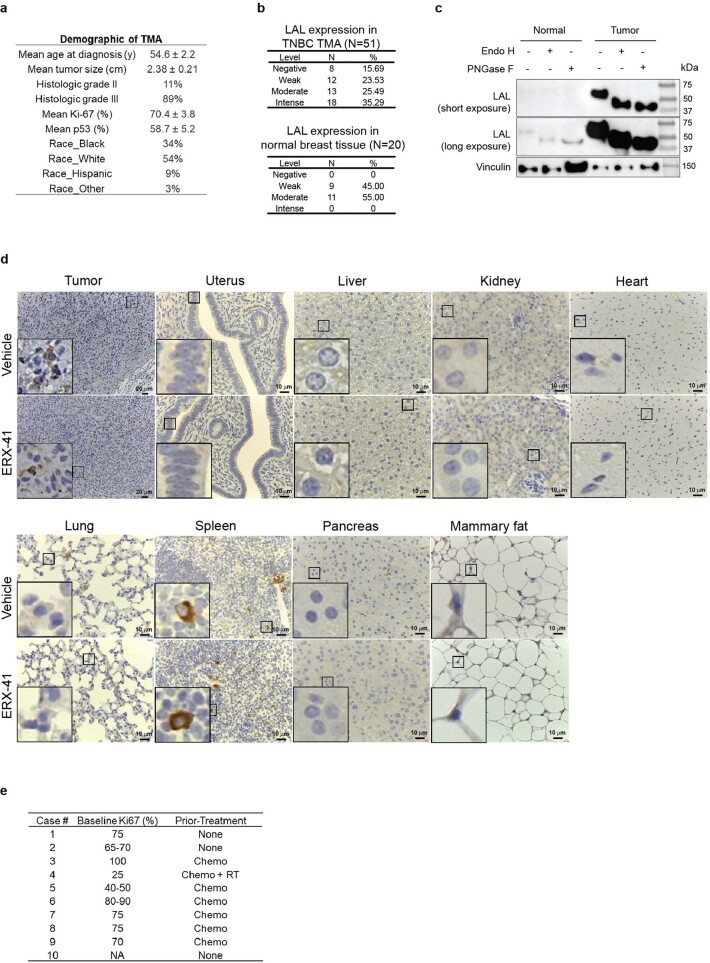
*LIPA* is a viable molecular target in TNBC. **a:** Table shows the demographics of patients from whom the TNBC TMA was constructed**. b:** Quantification of LAL expression levels in tissue microarray of 51 TNBC patients and 20 normal breast tissues, expressed as negative, weak, moderate or intense staining. **c:** Lysates TNBC tumor or adjacent normal tissues from the same patient were subject to Endo H or PNGase digestion. Sensitivity of LAL in patient tissues to glycosidase cleavage was evaluated by western blotting with vinculin control. **d:** After daily administration of 10 mg/kg ERX-41 for 1 week, tissues from the implanted MDA-MB231 xenograft tumor and various organs were harvested and evaluated for expression of LAL protein using immunohistochemistry with the Santa Cruz monoclonal antibody that recognizes both mouse and human LAL protein. Inset shows detail of the highlighted area. **e:** Table details the demographics of patients from whom primary tumor explants were obtained. The experiments in **c** were done once with 4 different patient samples with similar results. Uncropped blots for **c** are provided. Source data

We then evaluated LAL protein expression in TNBC using tissue microarray (Extended Data Fig. 7a). We found that >80% of primary TNBC tumors had significant and detectable LAL protein expression (Fig. 6a and Extended Data Fig. 7b); in contrast, normal breast tissue had lower LAL expression (Fig. 6b and Extended Data Fig. 7b). Protein expression was noted to be higher in TNBC tumors than in adjacent normal breast tissue (Fig. 6c,d). Importantly, glycosylation of LAL in TNBC tumors is similar to that observed in TNBC cells (Extended Data Fig. 7c). Analysis of publicly available datasets (The Cancer Genome Atlas (TCGA)) indicated that high LAL expression correlated with significantly worse overall survival outcomes in patients with BC (Fig. 6e). These data suggest that LAL is a viable molecular target in TNBC.

The minimal toxicity of ERX-41 in vivo prompted our evaluation of LAL expression in normal mouse organs by immunohistochemistry (IHC). We noted that LAL expression in multiple mouse tissues, including uterus, liver, kidney, heart, lung, spleen, pancreas and mammary fat pad, was much lower than in tumor tissue (Extended Data Fig. 7d). LAL expression was lowest in heart, pancreas and mammary fat pad, intermediate in liver and kidney and highest in the spleen. Given the role of the spleen in the lymphatic system, our previous analyses showing that ERX-41 had no effect on plasma cells are of relevance. These data suggest that enhanced expression of LAL in tumors may account for their sensitivity to ERX-41.

To ascertain that ERX-41 has activity against primary TNBC tumors, we leveraged our previous experience with ex vivo patient-derived explant (PDE) cultures (Fig. 6f)^5,7–9^. PDE cultures maintain the native tissue architecture and better recapitulate the heterogeneity of human TNBC in a laboratory setting (Extended Data Fig. 7e). We noted that ERX-41 had significant activity, as evidenced by decreased proliferation (Ki67 staining) and increased apoptosis (cleaved caspase 3 staining) of primary TNBC PDEs (Fig. 6g–i). Immunohistochemical evaluation of UPR markers in these explants showed enhanced p-PERK, CHOP (protein product of *DDIT3* gene) and p-eIF2-α staining within 24 h after treatment with ERX-41 (Fig. 6i). These data further validate the potential utility of ERX-41, specifically in patients who would receive the drug.

**Extended Data Fig. 8:**
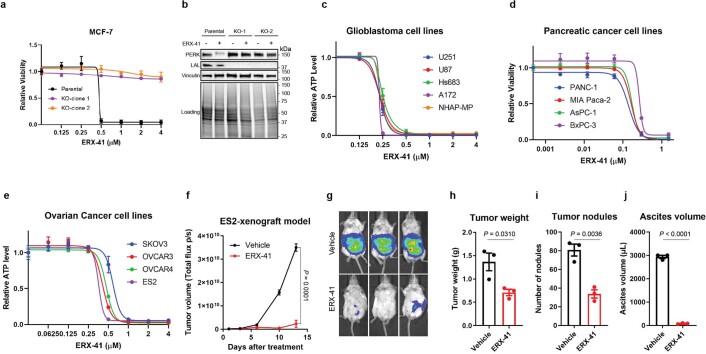
*LIPA* is a viable molecular target in other solid tumors. **a:** Dose response curves of parental and *LIPA* KO clones of ERα + MCF-7 cells show effect of increasing concentrations of ERX-41 at 48 h after treatment. Data are presented as mean ± SEM, *n* = 3 biologically independent experiments. **b:** Evaluation of the effect of 1μM ERX-41 on the induction of p-PERK (up shifted band) in parental and *LIPA* KO clones of ERα + MCF-7 cells. **c-e:** CTG assays showing the dose response curves for the effect of ERX-41 on cell viability of a number of glioblastoma **(c)**, pancreatic **(d)** and ovarian **(e)** cancer cell lines *in vitro*. Data are presented as mean ± SEM, *n* = 3 biogically independent experiments. **f-j:** ES2/GFP/LUC cells were injected i.p. and tumors established. Mice (n = 3) were treated with vehicle or ERX-41 (10 mg/kg/day/i.p). Tumor volume **(f)**, tumor picture **(g)**, tumor weight **(h)**, number of tumor nodules **(i)** and ascites volume **(j)** are shown. Data are presented as mean ± SEM, *n* = 3 mice per group. Significance was determined by unpaired two tailed Student’s *t*-test. The experiments in **b** were done twice independently with similar results. Numerical source data for **a, c, d, e, f, h, i, j** and uncropped blots for **b** are provided. Source data

We postulated that ERX-41 would have activity in other tumors with high basal levels of ER stress, such as ER-α^+^ BC, glioblastoma and pancreatic and ovarian cancers. Initial evaluation confirmed responsiveness to ERX-41 in vitro (Extended Data Fig. 8a,c–e). Importantly, we have shown that knockdown of LAL expression in these cell lines using CRISPR abrogated the ability of ERX-41 to induce ER stress (Extended Data Fig. 8b, for ER-α^+^ MCF-7 cells). We also confirmed that ERX-41 has activity against both ER-α^+^ BC PDX (WHIM-20) (Fig. 6j) and an ovarian cancer cell line xenograft (ES2) (Extended Data Fig. 8f–j), and PDX (OCa-PDX-38) (Fig. 6k). These data indicate that ERX-41 has activity against multiple tumors by targeting LAL and inducing ER stress.

### ERX-41 targets *LIPA* in ER and is independent of LIPA lipase activity

**Fig. 7:**
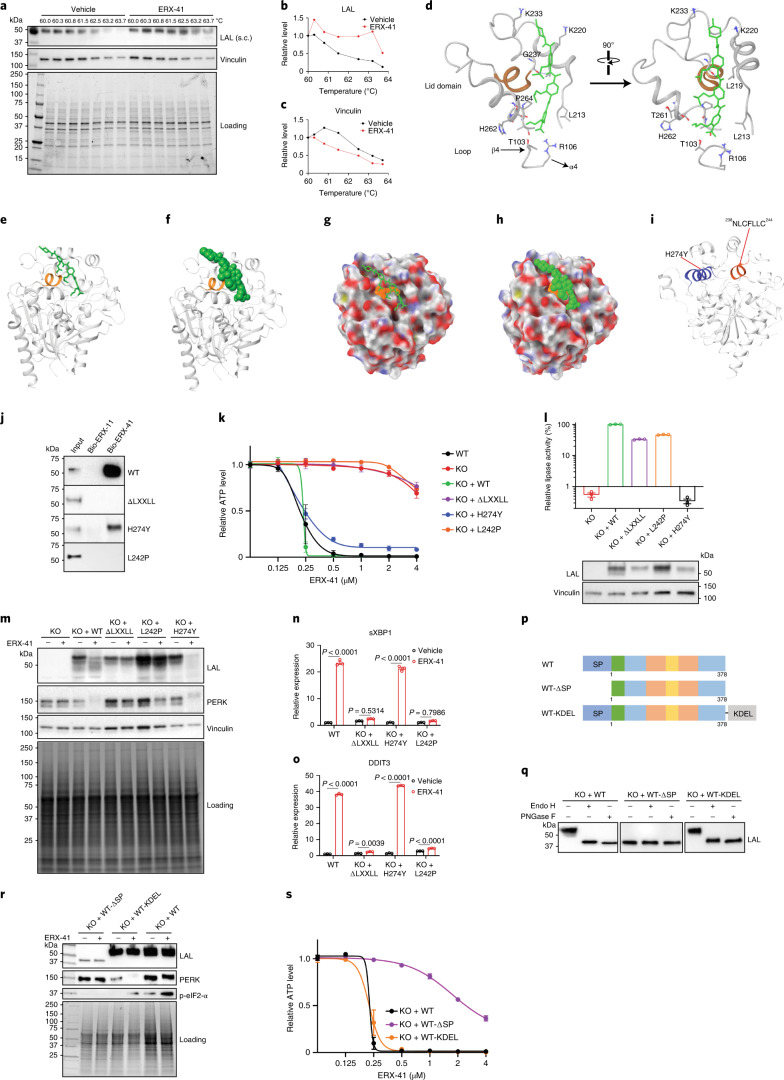
ERX-41 binds LAL and is independent of lipase activity. **a**–**c**, Cellular thermal shift assays show heat stability of LAL or vinculin protein in SUM-159 cells (**a**); relative levels of LAL (**b**) and vinculin (**c**) were quantitated and graphed. **d**, Predicted binding of ERX-41 (colored in green) to LAL (PDB code: 6V7N, LXXLL domain colored in orange). **e**–**f**, Representation of ERX-41 as a stick model (**e**) or space-filled model (**f**) bound to LAL in ribbon diagram. **g**–**h**, Representation of ERX-41 as a stick model (**g**) or space-filled model (**h**) bound to the modeled surface of LAL. **i**, PDB structure of LAL protein showing relative positions of catalytic (with H274Y MT) and LXXLL domains (L242P MT). **j**, Lysates from described SUM-159 cells were incubated with biotinylated ERX-11/ERX-41, subjected to streptavidin binding and eluates evaluated for LAL pulldown by immunoblotting. **k**, Dose–response curve of ERX-41 in described SUM-159 cells was measured by CellTiter-Glo. Data presented as mean ± s.e.m.; *n* = 4 for all groups except KO + L242P, with *n* = 3 biologically independent samples. **l**, Top, evaluation of basal lipase activity in parental SUM-159 (WT), clones with *LIPA* KO (KO) or with reconstitution of WT-LIPA (KO + WT), H274Y MT-*LIPA* (KO + H274Y), ΔLXXLL MT-*LIPA* (KO + ΔLXXLL) or L242P MT-*LIPA* (KO + L242P). Data presented as mean ± s.e.m.; *n* = 3 biologically independent samples. Bottom, protein expression of LAL and vinculin control. **m–o**, Effect of vehicle or 1 μM ERX-41 on induction of UPR genes at protein (**m**) or RNA level (**n**,**o**) in recombinant SUM-159 cells (**n**, sXBP1; **o**, DDIT3). Data presented as mean ± s.e.m.; *n* = 3 independent biological samples. Significance was determined by two-way ANOVA with Tukey’s multiple comparisons test. Adjusted *P* values are shown. **p**–**s**, Recombinant cDNAs with WT-*LIPA*, lacking signal peptide (WT-∆SP) or with ER-retention peptide (WT-KDEL) were synthesized (**p**) and stably transduced in SUM-159 cells with LIPA KO. **q**, Sensitivity of expressed recombinant LAL proteins to Endo H or PNGase F cleavage. **r**, Effect of vehicle or 1 μM ERX-41 on induction of UPR genes at protein level in recombinant SUM-159 cells. **s**, Dose–response curves to ERX-41 in these SUM-159 cells were performed by CellTiter-Glo and graphed. Data presented as mean ± s.e.m.; *n* = 3 biologically independent samples. Immunoblot studies in **a**–**c**,**j**,**m**,**q**,**r** were performed twice independently, with similar results. Numerical source data for **d**,**g**–**i**,**k** and uncropped blots for **c** are provided. Source data

We used cellular thermal shift assays to confirm that ERX-41 binds to the protein product of the *LIPA* gene, LAL protein. Our studies indicate that ERX-41 was able to stabilize LAL within the cell and shifted thermal sensitivity, suggesting that ERX-41 binds to LAL (Fig. 7a,b). In contrast, ERX-41 did not affect vinculin thermal stability (Fig. 7c). Our subsequent assays confirmed binding of LAL to ERX-41 (Fig. 7j) and indicate that ERX-41, but not ERX-11, binds to LAL in the cellular context of TNBC.

**Extended Data Fig. 9:**
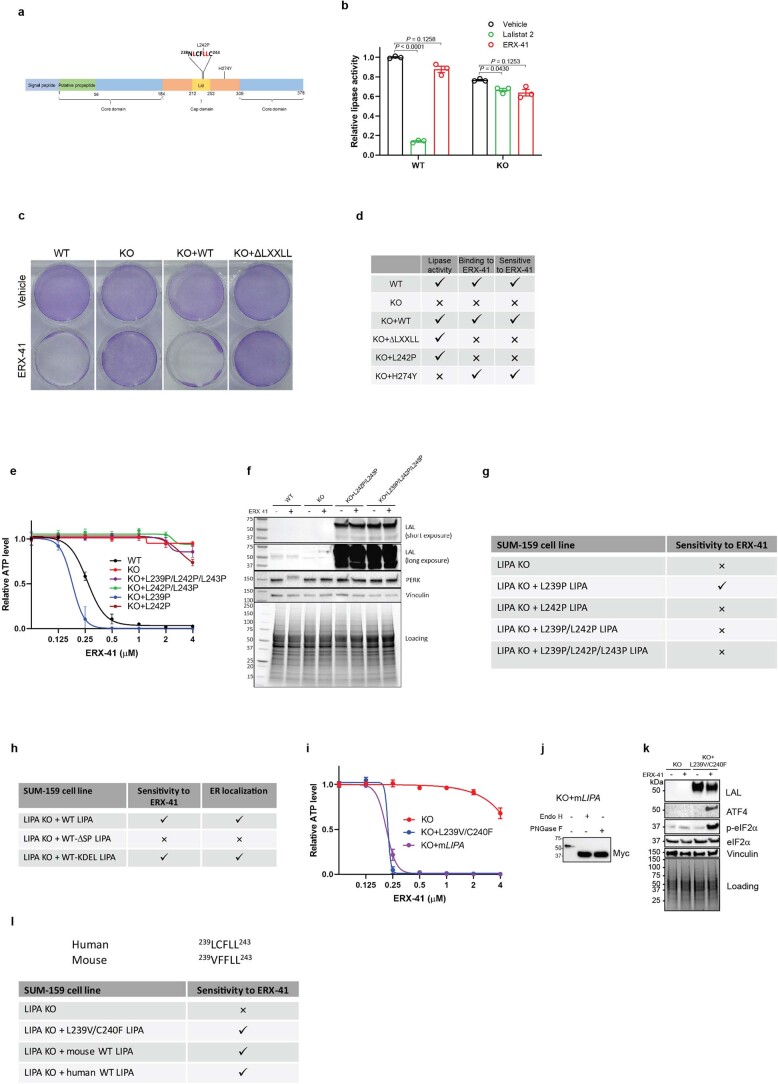
ERX-41 is independent of LAL lipase activity but dependent on LXXLL motif. **a:** Structure of LAL (not to scale) is depicted with 21aa N-terminal signal peptide, putative propeptide (1-56), core (1-183 and 309-378) and cap domain (184-308). The flexible lid domain (212-251), LXXLL motif (238-244), L242P point mutation and LAL lipase dead point mutation H274Y are highlighted. **b:** Effect of ERX-41 and Lalistat 2 (known LAL lipase inhibitor) on lipase activity of parental SUM-159(WT) and *LIP*A KO clones. Data are presented as mean ± SEM, *n* = 3 replicates. Significance was determined by two-way ANOVA with Tukey’s multiple comparisons test with adjusted *P* values shown. **c:** Crystal violet staining of parental SUM-159(WT), *LIPA* KO(KO) clones and *LIPA* KO clones with reconstitution of WT *LIPA* (KO + WT), or ∆LXXLL MT-*LIPA* (KO + ∆LXXLL) after 3d treatment with vehicle or 1 μM ERX-41. **d**: Tabulated summary of data in main Fig. 7j, k and l. **e-g**: **Effect of LXXLL mutations**: cellTiterGlo assays show dose responses to ERX-41 in described SUM-159 cells (**e**). Data are presented as mean ± SEM, *n* = 3 biological replicates. Effect of vehicle or 1μM ERX-41 on p-PERK induction in these SUM-159 cells is shown by western blotting (**f**) with tabulation (**g**). **h**: Tabulated summary of data in main Fig. 7p–s. **i-l**: **Effect of mouse** ***LXXLL sequences***: SUM-159 cells with *LIPA* KO were reconstituted with myc-tagged recombinant *LIPA* with either human *LIPA* with substituted mouse LXXLL sequence (L239V/C240F) or complete mouse *LIPA* cDNA (m*LIPA*). Dose responses to ERX-41 in these SUM-159 cells using CellTiterGlo are graphed (**i**). Data are presented as mean ± SEM, *n* = 3 biological replicates. Sensitivity of expressed recombinant LAL proteins to Endo H and PNGase F cleavage are shown (**j**). Effect of vehicle or 1μM ERX-41 on induction of UPR genes is shown by western blotting (**k**), and results tabulated (**l**). The experiments in **c, f, j and k** were done twice independently with similar results. Numerical source data for **b, e, i** and uncropped blots for **f, j, k** are provided. Source data

To study how ERX-41 interacts with LAL, we used in silico molecular docking simulation to evaluate potential binding sites of ERX-41 on LAL (Fig. 7d–i). We note that LAL has a single ^239^LXXLL^243^ motif and that ERX-41 (shown in green in Fig. 7d–i) could potentially interact with this LXXLL motif (LXXLL motif shown in orange; Fig. 7d–i and Extended Data Fig. 9a). Structurally, the lipase activity of LAL appeared to be spatially distinct from the LXXLL motif: previous studies have identified that the point mutation H274Y in LAL helix 13 abrogates the lipase function of *LIPA* (Fig. 7i)^10^. ERX-41 showed no inhibition of lipase activity of *LIPA* while, in contrast, Lalistat 2 (specific inhibitor of LAL lipase activity) attenuated lipase activity in SUM-159 cells (Extended Data Fig. 9b). The specificity of Lalistat 2 for LAL is confirmed by its lack of inhibitory activity on lipase function (from other lipases) in *LIPA* KO cells^11,12^.

To study the effect of these *LIPA* domains, we synthesized *LIPA* plasmid constructs under a constitutive promoter, including wild-type (WT) *LIPA* (WT-LIPA), H274Y mutant *LIPA* (H274Y MT-*LIPA*), ΔLXXLL mutant *LIPA* (deletion of^238^NLCFLLC^244^) and L242P mutant *LIPA* (point mutation of the second L in the LXXLL motif). We then confirmed direct interaction of ERX-41 with *LIPA* using biotinylated ERX-41 pulldown, which indicated interaction between ERX-41 and proteins encoded by WT-*LIPA* and H274Y MT-*LIPA* but not by ΔLXXLL MT-*LIPA* or L242P MT-*LIPA* (Fig. 7j). Reconstitution of WT-*LIPA* (KO + WT) in SUM-159 cells with *LIPA* KO restored sensitivity to ERX-41, with an IC_50_ of 250 nM (Fig. 7k). Interestingly, reconstitution with the lipase-incompetent H274Y MT-*LIPA* (KO + H274Y) also restored sensitivity to ERX-41 (Fig. 7k). In contrast, reconstitution of lipase-competent ΔLXXLL MT-*LIPA* (KO + ΔLXXLL) did not restore sensitivity to ERX-41, suggesting that the LXXLL motif is critical for LAL–ERX-41 interaction (Fig. 7k and Extended Data Fig. 9c,d). Evaluation of the lipase activity of these constructs confirmed that WT-*LIPA*, ΔLXXLL MT-*LIPA* and L242P MT-*LIPA* had lipase activity while H274Y MT-*LIPA* did not (Fig. 7i and Extended Data Fig. 9d). Further detailed mutational evaluation of the ^239^LXXLL^243^ domain indicated that the leucine at the 242 position is critical for ERX-41 activity (Fig. 7j–o and Extended Data Fig. 9d–g). In contrast, the leucine at the 239 position is not critical for ERX-41 binding or activity (Extended Data Fig. 9e–g). These data are validated by the inability of ERX-41 to affect proliferation or induce ER stress in SUM-159 clones with reconstitution of lipase-competent LXXLL motif single mutations (KO + L242P), double mutations (KO + L239P/L242P) or triple mutations (KO + L239P/L242P/L243P) (Extended Data Fig. 9e–g). These findings are further supported by the ability of WT-*LIPA* and H274Y MT-*LIPA*, but not ΔLXXLL MT-*LIPA* or L242P MT-*LIPA*, to restore the ability of ERX-41 to induce ER stress at the protein level (shown by activation of PERK in Fig. 7m) and UPR genes at the RNA level (sXBP1 and DDIT3 levels in Fig. 7n,o) in SUM-159 cells with *LIPA* KO. These data, taken together, indicate that ERX-41 interacts with LAL through residues in its LXXLL domain and that its ability to induce ER stress and cell death in TNBC is independent of the lipase activity of LAL (Extended Data Fig. 9g).

We then evaluated whether LAL localization to the ER is critical for LAL function by using additional LAL recombinants with altered subcellular localization (Fig. 7p–s). We noted that a recombinant *LIPA* complementary DNA lacking the signal peptide (WT-∆SP) is neither localized to the ER nor glycosylated (Fig. 7q), nor able to restore the ability of ERX-41 to cause ER stress (Fig. 7r) or affect cell proliferation (Fig. 7s). In contrast, a recombinant *LIPA* cDNA with an added KDEL sequence at the C terminus (WT-KDEL) is localized to the ER, is glycosylated (Fig. 7q) and restores the ability of ERX-41 to cause ER stress (Fig. 7r) and affect cell proliferation (Fig. 7s). These data indicate that ER localization of LAL is critical for cellular responsiveness to ERX-41 (Fig. 7i, tabulated in Extended Data Fig. 9h).

Finally, since mouse LAL has a slightly different LXXLL sequence (VFFLL), we confirmed that this altered sequence in the context of human *LIPA* or the entire mouse *LIPA* cDNA is glycosylated and could bind to ERX-41 and restore responsiveness (ER stress and cell death) to ERX-41 in SUM-159 *LIPA* KO cells (Extended Data Fig. 9i–l). In conjunction with earlier findings that a tumor cell line of murine origin (D2A1) is sensitive to ERX-41 (Fig. 2f–h), these data indicate that the nontoxic characteristics of ERX-41 in the mouse cannot be attributed to species differences in LAL sequences.

### ERX-41 targeting of *LIPA* disrupts protein folding in the ER

**Fig. 8:**
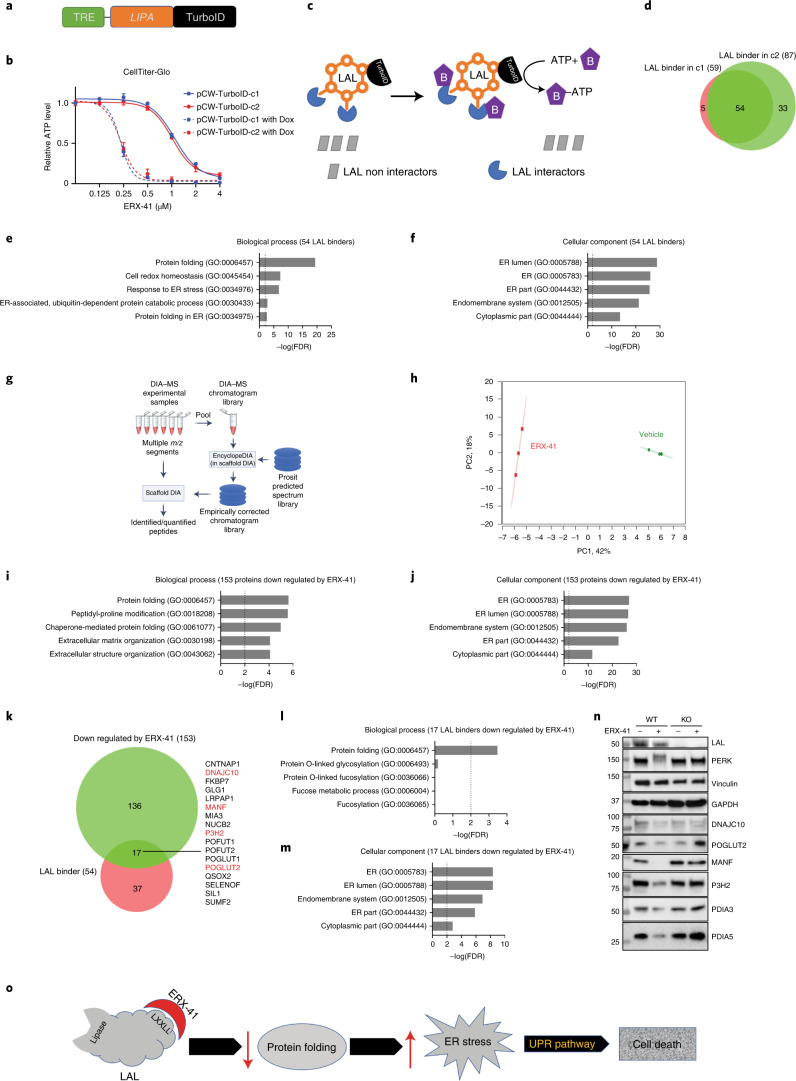
ERX-41 binding of *LIPA* decreases expression of ER-resident proteins involved in protein folding. **a**, Structure of recombinant *LIPA* fused to TurboID under control of a doxycycline (Dox)-inducible promoter (TRE). **b**, CellTiter-Glo studies showing the effect of ERX-41 on the growth of two independent clones of SUM-159 cells, with *LIPA* KO stably transduced by the recombinant *LIPA-*TurboID construct without and with doxycycline induction. Data presented as mean ± s.e.m.; *n* = 3 biologically independent samples. **c**, Schematic showing that LAL interacting proteins (blue) are biotinylated while LAL noninteracting proteins (gray boxes) are not. Biotinylated proteins were then isolated by streptavidin binding and identified by unbiased mass-spectrometric analyses. **d**, Venn diagram showing overlap between LAL binders identified in two clones. **e**,**f**, GO analysis showing both the biological processes (**e**) and cellular component (**f**) of the top 54 LAL binders. **g**, Schematic of global DIA–MS. **h**, PC analyses showing the effect of ERX-41 on the expression of a number of proteins. **i**,**j**, GO analysis showing both the biological processes (**i**) and cellular component (**j**) of the top 153 ERX-41-downregulated proteins. **k**, Venn diagram showing overlap between ERX-41-downregulated proteins and LAL binders, with the protein list displayed. **l**,**m**, GO analysis showing both the biological processes (**l**) and cellular component (**m**) of the 17 ERX-41-downregulated and LAL-binding protein binders. **n**, Immunoblot showing the effect of ERX-41 in SUM-159 cells (WT) and SUM-159 cells with *LIPA* KO (KO) on the expression of LAL, ER stress markers and number of ER-resident proteins, including those identified in panel. Immunoblots were performed twice independently, with similar results. **o**, Model showing that ERX-41 binds to the LXXLL domain of LAL protein and induces ER stress, resulting in cell death. Numerical source data for **b**,**e**,**f**,**i**,**j**,**l**,**m** and uncropped blots for **n** are provided. FDR, false discovery rate. Source data

**Extended Data Fig. 10:**
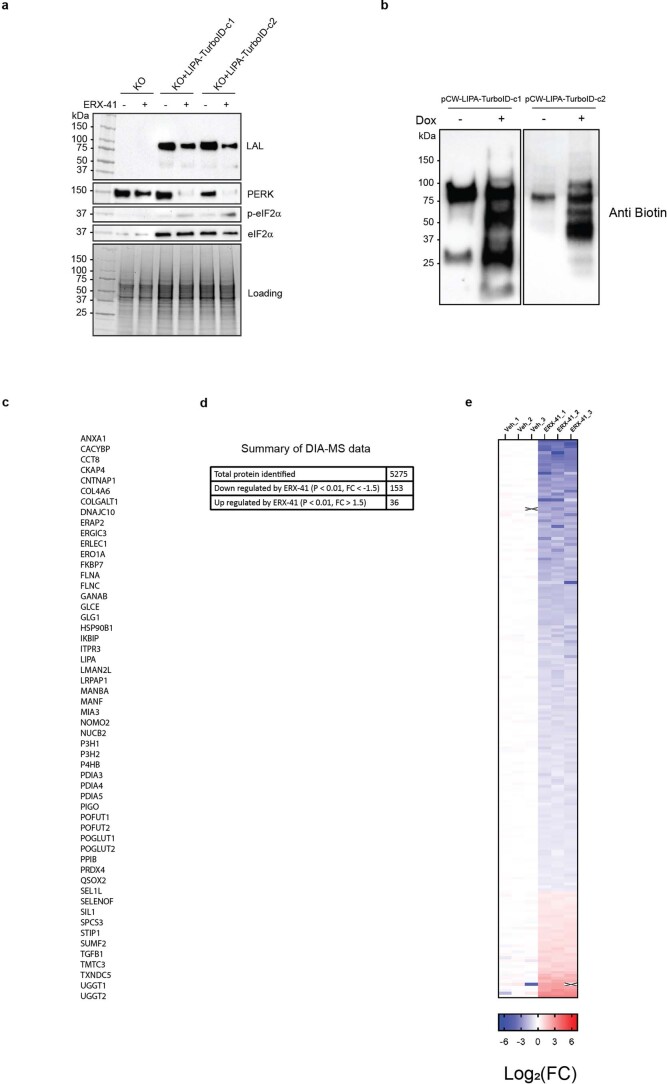
ERX-41 binding *LIPA* affects ER-resident proteins involved in protein folding. **a:** Effect of vehicle or 1μM ERX-41 on induction of UPR gene at the protein level in SUM-159 *LIPA* KO cells with or without *LIPA*-TurboID expression is evaluated by western blotting. **b:** Western blotting shows the induction of biotinylated proteins in two different clones expressing pCW-*LIPA*-TurboID after 2 days treatment of doxycycline. **c:** The gene names of proteins down regulated by ERX-41. **d:** Summary of DIA-MS data. **e:** The heatmap of differential expressed proteins (*P* < 0.01 and fold change > or < 1.5) identified by DIA-MS. The experiments in **a** and **b** were done twice independently with similar results. Numerical source data for **e** and uncropped blots for **a, b** are provided. Source data

To molecularly characterize how targeting of LAL causes ER stress, we first decided to define the LAL interactome using a recombinant LAL fused to TurboID (Fig. 8a). Importantly, this recombinant LAL could reconstitute sensitivity to ERX-41 in SUM-159 *LIPA* KO cells in CellTiter-Glo studies (Fig. 8b) and restore the ability of ERX-41 to cause ER stress (Extended Data Fig. 10a). LAL interactors were biotinylated, isolated by streptavidin pulldown (Extended Data Fig. 10b) and identified by unbiased MS analyses in two independent experiments in two different cell clones (Fig. 8c,d). Gene ontogeny (GO) analysis of the 54 LAL interacting proteins (Extended Data Fig. 10c) revealed that four of the top five cellular processes are involved in critical ER protein maturation functions, including protein folding in the ER (Fig. 8e). Cellular component analyses indicated that LAL binders were most commonly localized to the ER (Fig. 8f).

We then used a next-generation proteomic method, DIA–MS (Fig. 8g). Utilizing an unbiased quantitative DIA–MS approach, we identified that short-term (6 h) ERX-41 treatment significantly affected the expression of 189 cellular proteins (Extended Data Fig. 10d,e), including 153 down- and 36 upregulated proteins (PC analyses of proteins provided in Fig. 8h). GO analysis of the 153 ERX-41-downregulated proteins revealed that three of the top five cellular processes were involved in ER protein maturation functions, such as protein folding in ER (Fig. 8i), and that these proteins were localized to the ER (Fig. 8j).

We then combined data from both unbiased proteomic approaches to identify a core set of proteins that were both LAL binders and affected by ERX-41 treatment (Fig. 8k). Again, GO analyses of these 17 proteins confirmed their involvement in protein folding (Fig. 8l) and localization to the ER (Fig. 8m). We then validated the LAL dependence of ERX-41 activity using both LAL-expressing and LAL-KO cells: immunoblots showed that ERX-41 affects the expression of these proteins in LAL-expressing but not in LAL-KO cells (Fig. 8n). These data support the model that ERX-41 binding to LAL in the ER affects expression of several ER localized proteins involved in protein folding, causing significant ER stress/UPR leading to cell death. The lipase function of LAL is neither affected by ERX-41 nor is critical for ERX-41 activity; these findings are modeled in Fig. 8o.

## Discussion

Since TNBCs have high growth rates, they have sustained and enhanced demand for de novo protein synthesis, folding and maturation. TNBC tumor growth, metastasis, chemotherapy, hostile environmental conditions—such as hypoxia—and oxidative stress further jeopardize the fidelity of protein folding in their ER, and cause ER stress^13^. Compensatory UPR pathway proteins 78-kDa-glucose-regulated protein (GRP78), PERK and activating transcription factor 6 (ATF6) are overexpressed in TNBC, increased during TNBC progression and are correlated with poor patient survival in TNBC^14,15^. Several genome screens identified components of the ER stress pathway as targets of vulnerability in various cancers, including TNBC^16–18^. Although UPR can resolve ER stress and restore homeostasis, unresolved ER stress can be lethal to cells via ER stress-induced apoptosis. However, persistent and severe ER stress kills cancer cells by inducing their autophagy, apoptosis, necroptosis or immunogenic cell death.

In the present study, using a variety of biochemical and ultrastructural studies, we have shown that ERX-41 induces ER stress. Our confocal microscopy results showing ER stress are noteworthy, since ER luminal diameter (50–60 nm) is normally below the resolution of confocal microscopy (100–200 nm). Since ER dilation measured following ERX-41 treatment approached 600 nm, the true fold effect of ERX-41 on ER morphology is profound and significant. We observed that ERX-41 induces ER stress, shuts down de novo protein synthesis, blocks proliferation and induces apoptosis of TNBC in vitro, ex vivo and in vivo. Our results suggest that ERX-41 aggravates this already engaged system in TNBC to exhaust its protective features and cause apoptosis. In normal cells and tissues ERX-41 does not induce ER stress, suggesting that the basal level of ER stress and the compensatory UPR pathway may dictate responsiveness to ERX-41. These data indicate that ERX-41 targets a fundamental vulnerability in TNBC—the high basal level of ER stress—and may be able to overcome the inter- and intratumoral heterogeneity of TNBC.

Knockout of *LIPA* abrogates the ability of ERX-41 to induce ER stress, UPR, block de novo protein synthesis and cause cell death in vitro and in vivo. Reconstitution of *LIPA* in this KO system restores the ability of ERX-41 to induce ER stress, UPR and cause cell death. Importantly, reconstitution of a *LIPA* mutan*t* in this KO system that is incapable of binding ERX-41 does not restore the ability of ERX-41 to induce ER stress, UPR and cause cell death. Our identification of a single-point mutation within *LIPA* that abrogates responsiveness to ERX-41 represents a gold standard validation that LAL is the target of ERX-41. These data establish that the interaction between ERX-41 and LAL is critical for ERX-41-induced cell death. We have also shown that LAL binds to ERX-41 using two distinct assays—the cellular thermal shift assay in intact cells and biotinylated pulldown of LAL from cell lysates. Cocrystallization of LAL with ERX-41 will further inform structural details of this interaction, and are ongoing. Collectively, our data confirm the central role of *LIPA* in the activity of ERX-41 against TNBC.

Our unbiased proteomic studies offer some insight as to how ERX-41 binding to LAL causes ER stress. We have identified the interactome of LAL, which appears to be primarily composed of ER-resident proteins and proteins involved in critical ER maturation functions such as ER folding. Importantly, our unbiased global proteomic studies indicate that ERX-41 causes a decrease in the expression of several known ER-resident proteins and proteins involved in ER folding. These data suggest that ERX-41 binding to LAL causes downregulation of several ER proteins involved in protein maturation and effectively causes ER stress.

### Identification of *LIPA* as a targetable molecular vulnerability is the critical finding in this study

Since the lipase function of LAL is not targeted by ERX-41, we also noted that Lalistat 2, a known inhibitor of LAL lipase activity, does not induce ER stress or cause cell death in TNBC. Our studies suggest that LAL localization to the ER is critical for ERX-41 activity: a recombinant LAL lacking the signal peptide does not become glycosylated or respond to ERX-41. Our chemistry-first approach enabled the identification of an uncharacterized function of LAL related to its ability to function as a molecular chaperone of ER-resident proteins involved in protein folding. While LAL protein levels in tumors have been inadequately profiled, the expression of *LIPA* messenger RNA appears to be highest in glioblastoma and breast, ovarian and pancreatic cancers, and our studies indicate that their representative tumors are sensitive to ERX-41 treatment both in vitro and in vivo. Further studies are needed to evaluate whether the LAL expression levels and basal level of ER stress in tumors could serve as biomarkers of cellular response to ERX-41.

In conclusion, this manuscript reveals the important finding of a potent therapeutic agent (ERX-41) with a clear molecular target (LAL) and mechanism of action (disruption of protein folding and induction of ER stress) that may have utility in treating patients with multiple solid tumors.

## Methods

Our research complies with all relevant ethical regulations, and was performed in accordance with UTHSA and UTSW IACUC approved protocols.

### Chemicals and synthetic procedure

All chemical reagents and solvents were obtained from commercial sources and used without additional purification. ^1^H and ^13^C nuclear magnetic resonance (NMR) spectra were recorded on a Bruker Avance III HD 600-MHz NMR spectrometer. Chemical shifts (δ) are reported in ppm from an internal standard of residual DMSO-d6 (2.50 and 39.5 ppm in ^1^H and ^13^C-NMR spectra, respectively). Data are reported as follows: chemical shift (δ), multiplicity (s, singlet; d, doublet; dd, doublet of doublet; t, triplet; q, quartet; br s, broad singlet; m, multiplet), coupling constant (*J*) in Hertz (Hz), integration. Mass spectra were recorded on a Shimadzu AXIMA Confidence MALDI-TOF mass spectrometer (nitrogen UV laser, 50 Hz, 337 nm) using α-cyano-4-hydroxycinnamic acid as matrix.

The overall scheme of ERX-41 synthesis is shown in Supplementary Note. Synthesis of compound 2:compound 1 was synthesized as previously reported. *Trans*-4-methylcyclohexylamine (0.73 g, 6.4 mmol) was added to a solution of compound 1 (2.7 g, 3.2 mmol), HATU (1.4 g, 3.7 mmol), DIEA (1.2 ml, 6.9 mmol) and DMF (30 ml). The reaction mixture was stirred at room temperature (RT) for 24 h and then diluted with EtOAc (100 ml) and 0.5 N HCl (100 ml). Layers were separated and the aqueous layer extracted with EtOAc (100 ml). Organic layers were combined, washed with 0.5 N HCl and brine, dried over anhydrous sodium sulfate and concentrated under reduced pressure. The resulting solid was washed with EtOAc and dried in vacuo to yield compound 2 as a white solid (1.75 g). The product was used in the following reaction without further purification.

For synthesis of ERX-41, please refer to the scheme shown in Supplementary Note. Concentrated HCl (30 ml) was added to a solution of compound 2 (1.75 g) and THF (300 ml). The reaction mixture was stirred at RT for 24 h and then concentrated under reduced pressure. The resulting solid was washed with MeOH and dried in vacuo to yield ERX-41 as a light-yellow solid (1.3 g, 57% over two reaction steps). ^1^H NMR (DMSO-*d*_6_, 600 MHz): *δ* 9.85 (br s, 1H), 9.45 (br s, 1H), 8.15 (d, *J* = 7.7 Hz, 1H), 8.02 (d, *J* = 8.4 Hz, 1H), 7.96 (d, *J* = 8.6 Hz, 1H), 7.92 (d, *J* = 8.2 Hz, 1H), 7.85 (s, 1H), 7.64 (s, 1H), 7.63 (d, *J* = 8.2 Hz, 1H), 7.60 (d, *J* = 8.4 Hz, 1H), 7.52 (br s, 1H), 7.51 (d, *J* = 6.2 Hz, 1H), 4.96 (t, *J* = 5.2 Hz, 1H), 4.29 (t, *J* = 5.0 Hz, 2H), 3.91 (d, *J* = 5.9 Hz, 2H), 3.90 (d, *J* = 5.9 Hz, 2H), 3.76 (q, *J* = 5.1 Hz, 2H), 3.75–3.71 (m, 1H), 2.15–2.07 (m, 2H), 1.84 (d, *J* = 12.4 Hz, 2H), 1.71 (d, *J* = 12.5 Hz, 2H), 1.40–1.31 (m, 3H), 1.05–1.03 (m, 1H, overlapped with the signal of CH(CH_3_)_2_), 1.03 (d, *J* = 6.6 Hz, 6H), 1.01–1.00 (m, 1H, overlapped with the signal of CH(CH_3_)_2_), 1.01 (d, *J* = 6.6 Hz, 6H), 0.90 (d, *J* = 6.6 Hz, 3 H). ^13^C NMR (DMSO-*d*_6_, 150 MHz): *δ* 164.7, 164.2, 163.6, 151.1, 150.8, 149.8, 141.4, 139.2, 131.9, 131.6, 129.8, 129.6, 125.0, 124.0, 122.2, 119.73, 119.66, 119.59, 114.2, 111.1, 110.9, 74.6, 74.5, 71.5, 59.2, 48.4, 33.8, 32.3, 31.6, 27.9, 27.8, 22.2, 19.13, 19.05. MALDI-TOF (*m*/*z*): [M + Na]^+^ calculated for C_38_H_48_N_4_NaO_9_: 727.2, found 727.6.

The scheme of synthesis and characterization of ERX-11-9, ERX-11-16, ERX-11-30 and ERX-11-44 are also shown in Supplementary Note.

### Molecular docking study

AutoDock Tools 1.5.6 (ADT, Scripps Research Institute; RRID: SCR_012746) was used to create input PDBQT files of protein and ligand. The input file of human LAL was prepared using published coordinates (PDB code: 6V7N). Water molecules were removed from the protein structure and hydrogens added. All other atom values were generated automatically by ADT. The grid box was centered on the helical motif (^238^NLCFLLC^244^), and to facilitate free movement of the ligand. The grid box was set to 35 × 35 × 35 Å, with the *x*, *y* and *z* coordinates of the center of the grid box set to 123, 29 and 140, respectively. The input file of ERX-41 was created from its energy-minimized conformation using ADT. Docking calculation was performed with AutoDock Vina 1.1.2. A search exhaustiveness of 16 was used, and all other parameters were left as default values. Predicted binding mode was visualized using Maestro (v.9.1, Schrödinger; RRID: SCR_016748).

### Cell culture

All human BC cell lines were directly obtained from either ATCC, UTSW or the UTHSA ObGyn core and cultured according to ATCC guidelines. All cell lines utilized were free of mycoplasma contamination. Authentication was performed by short-tandem repeat profiling in the UTSW sequencing core facility. All cell lines used in this paper, sources and culture conditions are listed in Supplementary Table 1.

### Estrogen receptor binding

LanthaScreen TR–FRET ERα Coactivator Assay (no. A15885, Life Technologies) was performed as per the manufacturer’s instructions. Compounds were tested in the range 0.0003–16.66 μM using serial dilutions, with assays in antagonist assay mode. Final assay buffer composition included 3.5 nM ER-α-LBD (GST), 250 nM fluorescein-conjugated coactivator PGC1a peptide, 5 nM terbium (Tb)-labeled anti-GST antibody and 5 nM estradiol. The plate was incubated at RT for 2 h and FRET analyzed on a PHERAstar microplate reader with the settings excitation 340 nm, emission 495 and 520 nm. The emission ratio (520/495) was analyzed and plotted. Curves were generated using a sigmoidal dose–response equation (variable slope) in GraphPad Prism 9.0 software.

### Cell viability assays

Cells were seeded in 96-well plates (2 × 10^3^ cells per well) 1 day before treatment. Cells were treated with varying concentrations of ERX-41 or ERX-11 analogs for 3–6 days. The effects of ERX-41 and ERX-11 analogs on cell viability were then measured in triplicate or sextuplicate with multiple biological replicates using WST-1 (Promega), MTT as previously described^19^ or CellTiter-Glo 2.0 (Promega) assay.

### Live-cell imaging

Live-cell images were acquired using a Lionheart FX automated microscope (BioTek). Cells were plated 1 day before experiments. For cell death assay, 0.2 μM SYTOX Green was added to the plate 15’ min beforehand.

### CRISPR screen

The Human Brunello CRISPR knockout pooled library was purchased from Addgene (no. 73178; through D. Root and J. Doench). The library containing lentivirus was transduced into cells in biological replicate or triplicate at a multiplicity of infection of ~0.5 and minimum 500× coverage. Two days after transduction, uninfected cells were removed with puromycin selection. The library containing cells was treated with either vehicle or ERX-41 for 2 weeks. Genomic DNA was extracted from identical numbers of cells. The single-guide RNA cassette was retrieved by PCR, followed by next-generation sequencing (NGS). NGS data were analyzed by MAGeCK^20^.

### Lentiviral construct cloning

LentiCRISPR v.2 vector was used for CRISPR KO experiments. sgRNAs, which were chosen from the human CRISPR knockout pooled library, were cloned into lentiCRISPR v.2 vector^21^. Human *LIPA* cDNA (GenBank: BC012287) or mouse *LIPA* cDNA (NM_021460) was cloned into pWPI for overexpression experiments. For the TurboID experiment, the *LIPA*-TurboID sequence was cloned into pCW57-MCS1-2A-MCS2 vector. LentiCRISPR v.2 was purchased from Addgene (no. 52961; through F. Zhang). pWPI was purchased from Addgene (no. 12254; through D. Trono). pCW57-MCS1-2A-MCS2 was purchased from Addgene (no. 71782; through A. Karpf). Since *LIPA* overexpression was introduced in a *LIPA* CRISPR KO background, to avoid being targeted by CRISPR–Cas9, the *LIPA* sgRNA target sequence including PAM, ‘tt aac cga att cct cat ggg agg’, was mutated to ‘tt aac cga att cct caC ggA agA’ in all of our *LIPA* overexpression constructs, without protein coding change. An In-Fusion Cloning kit (Takara) was used to generate different mutants of *LIPA*.

### Live-cell confocal and Airyscan imaging

To visualize the ER structure, stable SUM-159 cell lines expressing mCherry-RAMP4 were established. pLenti-X1-hygro-mCherry-RAMP4 was purchased from Addgene (no. 118391; through J. Corn)^22^. Live-cell images were acquired with a Confocal Zeiss LSM880 Airyscan. ER tubule width was calculated by drawing 1-px-wide line scans perpendicular to the long axis of individual ER tubules. In MATLAB, peak intensity along each line scan was determined and the distance between half-maximum intensity on either side of the peak was measured. The code for this analysis is available at https://github.com/andmoo91/HalfMaxScript.

### Lentivirus production

Lentiviral constructs (lentiCRISPR v.2 for KO, pWPI for overexpression), along with helper plasmids Δ8.9 and VsVg, were transfected into HEK293T cells using polyethylenimine (PEI, 1 mg ml^–1^; Polysciences). Medium was changed the following day. Lentivirus was collected after an additional 48–72 h. Filtered (0.45 μm) lentivirus containing medium was used to infect cells, with 6 μg ml^–1^ polybrene.

### Pulldown assay

Cells were lysed in high-salt lysis buffer (1.25 mM Hepes pH 7.5, 400 mM NaCl, 0.5% NP-40, 5% glycerol and 2 mM MgCl_2_) supplemented with 1 mM DTT and 1/100 Halt protease inhibitor cocktail (Thermo Scientific) on ice for 15 min, followed by centrifugation at 20,000*g* for 20 min at 4 °C. The supernatant was mixed with a 1.67× volume of no-salt lysis buffer (1.25 mM Hepes pH 7.5, 5% glycerol and 2 mM MgCl_2_) supplemented with 1 mM DTT and 1/100 Halt protease inhibitor cocktail (Thermo Scientific). The lysate was incubated with either biotinylated ERX-11 or biotinylated ERX-41 overnight, followed by 1 h of incubation with M-270 streptavidin Dynabeads. After four washes with lysis buffer, samples were eluted by boiling in 2× Laemmli buffer. Bound proteins were resolved on SDS–polyacrylamide gel electrophoresis (SDS–PAGE) followed by immunoblotting.

### Immunoblotting

Whole-cell lysates from BC cells were prepared with 2× sample buffer (60 mM Tris-HCl pH 6.8, 2% sodium dodecyl sulfate, 10% glycerol) and, before loading, were mixed with loading buffer (5% 2-mercaptoethanol, 0.1% bromophenol blue) and boiled for denaturation^19^. Proteins were separated by SDS–PAGE and subjected to immunoblot analysis using antibodies. For detection of hyperphosphorylated forms of IRE1-α, we used Phos-tag-based immunoblot^23^. Briefly, SuperSep Phos-tag gels (Fujifilm) were used to separate protein samples. Phos-tag gels were washed 3× for 20 min with transfer buffer with 10 mM EDTA before transfer. For detection of nascent translated proteins, puromycin (final concentration 10 μg ml^–1^) was added 30 min before harvesting of cells. Samples were subjected to immunoblotting, and nascent proteins were detected by anti-puromycin antibody.

### RT–qPCR

Total RNA was prepared from BC cells using the RNeasy Mini Kit (Qiagen) according to the manufacturer’s protocols. Subsequently, total RNAs were reverse transcribed into cDNA using iScript Reverse Transcription Supermix (Bio-Rad) and with SYBR Green on an Illumina Real-Time PCR system. Primers utilized are listed in Supplementary Table 2.

### Cellular thermal shift assay

Cellular thermal shift assays were performed as previously described^24^. Briefly, cells were treated with DMSO or 10 μM ERX-41 for 30 min then trypsinized, pelleted and resuspended in PBS supplemented with Halt protease inhibitor cocktail (Thermo Scientific), with DMSO or 10 μM ERX-41. Resuspended cells were aliquoted into PCR strips. Cells were incubated in a thermal cycler (Bio-Rad) at gradient temperatures for 3 min, followed by incubation at 25 °C for 3 min. Cells were snap-frozen in liquid nitrogen and subjected to two freeze–thaw cycles. Samples were briefly vortexed and centrifuged at 20,000*g* for 20 min at 4 °C. Cleared cell lysates were mixed with a 1/3 volume of 4× Laemmli sample buffer. After boiling, cell lysates were resolved in SDS–PAGE followed by immunoblotting.

### LIPA staining in TNBC tissue microarray and primary normal breast tissue

TNBC tissue microarray (TMA) was generated by the UTSW Pathology Laboratory from 51 patients with high-grade TNBC. All cases were treatment naive. This study was approved by UTSW Institutional Review Board (no. STU-032011-117). TNBC diagnoses were based on IHC staining with image quantitation of ER, PR and HER2, and were confirmed by a board-certified BC pathologist (Y. Peng) at UTSW. Cerebellar tissue was included on a TMA slide as negative control. Slides cut from tissue blocks were immunostained for LIPA (1:500). An additional 20 slides were obtained from breast-reduction surgeries from women without known BC. Stained slides were scored manually per tissue core independently by a pathologist who was blinded to clinical data. Immunostaining data were registered semiquantitatively in staining intensity (0, no staining; 1, weak staining; 2, moderate staining; and 3, intense staining; representative staining examples ranging from 0 to 3 are provided).

### TNBC PDE studies

For PDE studies, excised tissue samples were processed and cultured ex vivo as previously described^5,7–9^. Deidentified patient tumors were obtained from UTSW Tissue Repository after institutional review board approval (no. STU-032011-187). Inclusion criteria included women with previous histologic confirmation of TNBC and who were undergoing surgical extirpation or biopsy of their primary tumor. Previous treatment with chemotherapy and/or radiation was allowed. Exclusion criteria included concurrent or previous diagnosis of other malignancies or previous evidence of ER-α^+^ or HER2^+^ BC. All cases were reviewed by UTSW Tissue Repository in advance, and patients provided consent for their tissue to be used for laboratory research. Only deidentified information was shared with the laboratory. None of the laboratory personnel had access to additional patient information. No attrition was noted. Briefly, fresh tumor samples were incubated on gelatin sponges in culture medium containing 10% FBS, followed by treatment with either vehicle or 2.5 μM ERX-41 for 72 h. Representative tissues were fixed in 10% formalin at 4 °C overnight and subsequently processed into paraffin blocks. Sections were then processed for IHC analysis.

### IHC

Immunohistochemical studies were performed as previously described^5^. Tissue sections were blocked in background sniper (Biocare Medical, no. RS966L) followed by overnight incubation with primary antibodies anti-Ki-67 (1:1,000), anti-CHOP (1:2,000), anti-PERK (phosphoT982; 1:150) and anti-phospho-eIF2-α (Ser51; 1:50), and subsequent secondary antibody incubation for 60 min at RT. Immunoreactivity was visualized using DAB substrate and counterstained with hematoxylin (Vector Laboratories). The percentage of ki67^+^ proliferating cells was calculated in five randomly selected high-power fields (×40).

### Immunofluorescence

Cells were fixed with ice-cold methanol and blocked with 5% normal serum and 0.3% Triton X-100 PBS. After overnight incubation with primary antibodies at 4 °C and four washes with 0.3% Triton X-100 PBS, samples were incubated with secondary antibodies at RT for 1 h. After four washes with 0.3% Triton X-100 PBS, cells were further washed with Hank’s balanced salt solution plus calcium and magnesium. Samples were then incubated with 0.2 μM ER Tracker Red (Molecular Probes) at 37 °C for 30 min. Cells were further washed with Hank’s balanced salt solution plus calcium and magnesium before mounting. Samples were imaged with a confocal microscope (Zeiss LSM 880).

### TEM

DMSO- or ERX-41-treated cells were fixed with 2.5% glutaraldehyde (Electron Microscopy Sciences) in 0.1 M sodium cacodylate pH 7.4 buffer (Electron Microscopy Sciences) for 20 min at RT. After rinsing with 0.1 M sodium cacodylate pH 7.4 buffer, cells were further fixed with 1% osmium and 0.8% K_3_Fe(CN_6_) in 0.1 M sodium cacodylate pH 7.4 buffer. After prestaining with 4% uranyl acetate in 50% ethanol, cells were dehydrated with series concentrations of ethanol (50–100%). After transitioning from propylene oxide to resin and embedding in Embed 812 resin (Electron Microscopy Sciences), cells were located using light microscopy and trimmed out. Sections (60–70 nm) were cut, mounted on formvar-coated grids and viewed with a transmission electron microscope (Tecnai G2 spirit, FEI) equipped with a LaB6 source using a voltage of 120 kV.

### RNA-seq

mRNA-seq sequencing library construction was performed using the TruSeq RNA Library Preparation Kit (Illumina) according to the manufacturer’s instructions. mRNA-seq data were analyzed by RNASeq Analysis Workflow (v.0.4.2 and v.0.5.15, https://git.biohpc.swmed.edu/BICF/Astrocyte/rnaseq) developed by the Bioinformatics Core Facility of UTSW. Briefly, FASTQ reads from the Genomics Core at UTSW were mapped to the human GRCh38 genome using HiSAT2 (RRID: SCR_015530). Differential expression analysis was performed using DEseq2 (RRID: SCR_015687). Abundances of transcripts were calculated using ballgown program. Values of fragments per kilobase of transcript per million mapped reads (FPKM) were used for Gene Set Enrichment Analysis (GSEA). GSEA was performed using GSEA software (RRID: SCR_005724) and C5 ontology gene sets (14,765 gene sets in total). mRNA-seq data are available from NCBI GEO under accession no. GSE168800.

### Lipase activity assay

Lipase activity assays were performed as described^25^. Briefly, a 0.345 mM substrate solution was prepared from 1.2 ml of 13.3 mM 4-MUP and 42 ml of 100 mM sodium acetate buffer pH 4.0, 1.0% (v/v) Triton X-100 and 3.0 ml of 0.5% (w/v) cardiolipin. For enzyme reactions, 50 μl of substrate in buffer solution, 40 μl of diluted cell lysate, 10 μl of DMSO and either 30 μM Lalistat 2 or 30 μM ERX-41 were combined in a black, 96-well plate. Plates were sealed with an adhesive aluminum film and incubated in an incubator at 37 °C for 1 h. Reactions were terminated using 200 μl of 150 mM EDTA at pH 11.5. Plate fluorescence was read immediately with a synergy H1 fluorescence microplate reader (BioTek) using a 365-nm excitation filter and a 450-nm emission filter. *LIPA* lipase activity was calculated by subtracting the enzymatic activity of inhibited (with Lalistat 2) reaction from that of the uninhibited (without Lalistat 2) reaction.

### Endo H and PNGase F assays

Endo H and PNGase F (New England Biolabs) assays were performed according to the manufacturer’s manual. Briefly, cell lysates were denatured at 100 °C for 10 min after the addition of glycoprotein denaturing buffer. For Endo H digestion, denatured cell lysate was incubated at 37 °C for 2 h following the addition of GlycoBuffer 3 and Endo H. For PNGase F digestion, denatured cell lysate was incubated at 37 °C for 2 h following the addition of GlycoBuffer 2, NP-40 (final concentration 1%) and PNGase F. Digested samples were mixed with a 1/3 volume of 4× Laemmli sample buffer. After boiling, samples were resolved in SDS–PAGE followed by immunoblotting.

### ELISpot

For ELISpot analysis of total IgM^+^ and IgG^+^ ASCs, Multi-Screen filter plates (Millipore) were activated with 35% ethanol, washed with PBS and coated with either anti-IgM (SouthernBiotech, no. 1020-01) or anti-IgG (SouthernBiotech, no. 1030-01) in PBS. Single bone marrow cell suspensions were prepared as above and cultured at 250,000 cells ml^–1^ in RPMI 1640 medium (Invitrogen) supplemented with FBS (10% v/v, Invitrogen), penicillin/streptomycin/amphotericin B (1% v/v) and 50 μM β-mercaptoethanol (RPMI-FBS) at 37 °C for 16 h. Following removal of supernatants, plates were incubated with either biotinylated goat anti-mouse IgM (SouthernBiotech, no. 1020-08) or goat anti-mouse IgG1 antibiotic (SouthernBiotech, no. 1070-08) for 2 h and, after washing, incubated with horseradish peroxidase-conjugated streptavidin. Plates were developed using the Vectastain AEC peroxidase substrate kit (Vector Laboratories). The stained area in each well was quantified using CTL ImmunoSpot software (Cellular Technology), and is depicted as the number of spots for quantification

### Flow cytometry

Single-cell suspensions were flushed from tibiae and fibulae with sterile DPBS using a 10-ml syringe and 30G needle. Red blood cells were removed by incubation with ACK lysis buffer (Lonzo) for 2 min. Bone marrow cells (2 × 10^6^) were first stained in Hank’s buffered salt solution plus 0.1% BSA (HBSS-BSA) for 20 min with fixable viability dye (FVD, eFluor 506; eBioscience. no. 50-246-097) and fluorophore-labeled mAbs to surface markers, including CD3 (APC-Cy7, Clone 17A2; BioLegend, no. 100221), IgD (FITC, Clone 11-26 c.2a; BioLegend, no. 405704), CD138 (PE-Cy7, Clone 281-2; BioLegend, no. 142514), SCA-1 (PerCP, Clone D7; BioLegend, no. 108121) and B220 (BV421, Clone RA3-6B2; BioLegend, no. 103240), in the presence of mAb Clone 2.4G2, which blocks FcγII and FcγIII receptors. After washing, cells were then fixed and permeablized by incubation for 1 h in 250 μl of BD Cytofix/Cytoperm buffer (BD Fixation/Permeablization Kit, no. 554714) at 4 °C. After washing twice with BD Perm/Wash buffer, cells were counted again and 10^6^ cells resuspended in 100 μl of BD Cytofix/Cytoperm buffer for intracellular staining with anti-Igκ mAb (PE, eBioscience, Clone 1871, no. MKAPPA04) for 30 min. After washing with BD Perm/Wash buffer, cells were analyzed by LSRII (BD). FACS data were analyzed by FlowJo software (BD).

### TurboID pulldown

TurboID pulldown assay was performed as previously described^26,27^. Briefly, pCW57-LIPA-TurboID transduced cells were treated with or without 1 mg ml^–1^ doxycycline for 48 h, with 50 mM biotin for 15 min and then harvested and lysed in RIPA buffer (Thermo Scientific) supplemented with Halt protease inhibitor cocktail (Thermo Scientific) and Phosphatase Inhibitor Cocktail sets I and II (MilliporeSigma) on ice for 15 min, followed by cenrifuging for 20 min at 20,000*g* and 4 °C. Cleared cell lysates were incubated with M-270 streptavidin Dynabeads overnight, then beads were washed twice with RIPA buffer (2 min), once with 1 M KCl (2 min), once with 0.1 M Na_2_CO_3_ (10 s), once with 2 M urea in 10 mM Tris-HCl (pH 8.0) (10 s) and twice with RIPA buffer (2 min). Enriched material was eluted from beads by boiling samples in 4× Laemmli buffer supplemented with 2 mM biotin and 20 mM DTT at 95 °C for 10 min. Eluted samples were resolved in SDS–PAGE, followed by MS with Lumos.

### GO analysis

Gene ontology analysis was performed using the Database for Annotation, Visualization and Integrated Discovery bioinformatics resource v.6.8 (https://david.ncifcrf.gov/tools.jsp)^28,29^

### TCGA analysis

Tcga_shiny (v.1.0.3, https://git.biohpc.swmed.edu/BICF/Astrocyte/tcga_shiny), developed by the Bioinformatics Core Facility of UTSW, was used for TCGA analysis.

### Antibodies

The antibodies used in this study are listed in Supplementary Table 2, with detailed information on vendor, catalog number, clone number (if relevant) and dilution.

### Primers

Primers used in this study and their sequences are listed in Supplementary Table 3.

### Venn diagram analysis

Venn diagram analysis was done using BioVenn (https://www.biovenn.nl/index.php)^30^.

### Animal studies

All animal experiments were performed using UTHSA and UTSW IACUC-approved protocols. For cell-based xenograft tumor assays, TNBC cells (2 × 10^5^ to 2 × 10^6^) were mixed with an equal volume of matrigel and implanted in the mammary fat pad of 6-week-old female athymic nude mice (RRID: RGD_5508395 (link)) as previously described^19^. BALB/c mice were used for the D2A1 syngeneic model. Once tumors had reached measurable size, mice were divided into control (vehicle) and ERX-41 (10 mg kg^–1^ PO) treatment groups. Mice bearing TNBC-PDX tumors were purchased from Jackson laboratory (nos. TM00089, TM00096, TM00098). The establishment of TNBC-PDX line UTPDX0001 is previously described^31^. The WHIM-20 (ERY537SMT) PDX model was purchased from Horizon Labs. OCa-PDX-38 was obtained from the UTHSA ObGyn tissue procurement core. When tumors had reached ~750 mm^3^ they were dissected into 2-mm^3^ pieces and implanted into the mammary fat pad of 6-week-old female NSG mice. When tumor volume had reached measurable size, mice were randomized for treatment and monitored daily for adverse toxic effects and tumor volume was measured every 3–4 days using calipers. For establishment of ES2 tumors, ES2/GFP/LUC cells were injected i.p. into female SCID mice and tumors measured twice weekly using the Xenogen in vivo imaging system. All mice were scheduled for euthanasia once tumor volume had reached 2,000 m^3^, as indicated in the IACUC protocols. In nine out of 12 experiments presented in this paper, mice were euthanized before tumors had reached 2,000 mm^3^. However, in three PDX tumor studies (Figs. 2k,o and 6k), some mice in vehicle-treated controls exceeded our goal of 2,000-mm^3^ tumor size due to the unexpected, unpredictable and rapid growth rate of their tumors. In each case, when tumor volume reached >2,000 mm^3^ we took the decision that the mice had reached endpoint criteria and were then scheduled for euthanasia. Since euthanasia scheduling typically took 1–4 days, tumors inevitably continued to grow rapidly, few reaching greater size (>2,000 mm^3^) at the time of sacrifice (Figs. 2k,o and 6k). In each case, we followed the humane endpoint guidelines as per IACUC policy and ensured that humane endpoints were not reached. All mice were active, with none showing any signs of moribund distress or weight loss over the entire experimental duration. At the end of each experiment the mice were euthanized and tumors were removed, weighed and processed for histological studies and protein analysis. Doses were selected based on a pilot maximal tolerated dose study of 10, 50 and 100 mg kg^–1^ ERX-41 for 14 days using C57BL6 mice. Mice were monitored daily for adverse toxic effects. IHC analysis was conducted as described previously^32^, and immunoreactivity was visualized using DAB substrate and counterstained with hematoxylin (Vector Laboratories). Control rabbit IgG staining was used as a negative control.

### KO studies

For xenograft KO tumor models, 1.8 × 10^6^ SUM-159 parental or *LIPA* KO cells were harvested with 1% trypsin-EDTA, washed and resuspended in sterile PBS. Cells were initially implanted into the mammary fat pad of 6-week-old female C.B-17 (SCID) mice purchased from Envigo. Mice were monitored twice weekly for tumor growth, measured by Vernier calipers, where tumor volume was calculated by the formula (length × width^2^)/2. Tumors were dissected into 2-mm^3^ cubes when they reached 800 mm^3^ in size and subsequently implanted into the mammary fat pad of 12 SCID female mice. Once tumor fragments had reached 150 mm^3^ in size, mice were randomly divided into either the vehicle group (*n* = 6, control, 0.3% hydroxypropyl cellulose, i.p.) or treatment group (*n* = 6, ERX-41, 10 mg kg^–1^ d^–1^ i.p.). Institutional guidelines were followed to determine experimental end points.

### Analytical LC–MS/MS conditions

Compound levels in plasma and tissues for in vivo PK studies were monitored by LC–MS/MS using an AB Sciex 6500^+^ QTRAP mass spectrometer coupled to a Shimadzu Prominence LC. Analytes were detected with the mass spectrometer in positive multiple-reaction monitoring mode by following the precursor to fragment ion transition 705.3 → 592.3. An Agilent C18 XDB column (5 µm, 50 × 4.6 mm^2^) was used for chromatography for PK studies with the following conditions: buffer A, dH_2_0 + 0.1% formic acid; buffer B, methanol + 0.1% formic acid, 0–1.0 min 5% B, 1–2 min gradient to 98% B, 2–3 min 98% B, 3.0–3.2 min gradient to 5% B, 3.2–4.5 min 5% B. Tolbutamide (transition 271.2–91.2) from Sigma was used as an internal standard (IS). PK studies were performed in MDA-MB-231 xenograft tumor-bearing female NOD-SCID mice. ERX-41 was given either PO or i.p. (10 mg kg^–1^ single dose). Animals were sacrificed in groups of three, and blood was obtained by cardiac puncture at each time point (0, 0.5, 1.5, 3, 6 and 24 h post dose) using the anticoagulant EDTA and plasma isolated by centrifugation. Tumor and liver were also collected and snap-frozen in liquid nitrogen after rinsing with PBS to remove surface-adherent blood. Tissues were homogenized in a threefold volume (weight by volume) of PBS to generate a homogenate. Next, 100 µl of plasma or tissue homogenate was mixed with 200 µl of methanol containing 0.15% formic acid and 15 ng ml^–1^ tolbutamide IS. Samples were vortexed for 15 s, incubated at RT for 10 min and spun twice at 16,100*g*, 4°C in a refrigerated microcentrifuge. The amount of compound present in plasma, tumor and liver was quantified by LC–MS/MS to determine the amount of compound in each matrix. Standard curves were generated using either blank plasma (Bioreclamation; RRID: SCR_004728) or blank tissue homogenate spiked with known concentrations of test compound, and processed as described above. Concentrations of drug in each time-point sample were quantified using Analyst 1.7.1 software (Sciex). A value of threefold above the signal obtained from blank plasma or tissue homogenate was designated the limit of detection (LOD). Lower limit of quantitation (LLOQ) was defined as the lowest concentration at which back calculation yielded a concentration within 20% of theoretical and that was >LOD. LLOQ for plasma and tumor was 0.1 ng ml^–1^, and 1 ng ml^–1^ in liver. PK parameters *C*_max_, *T*_max_, Terminal T_1/2_ (calculated as Ln(2)/*λ*_z_), AUC_last_ (area under the concentration time curve to the last measured value determined by linear trapezoidal rule), Cl (clearance measured as Dose/AUC_inf_) and *V*_z_ (volume of distribution based on the terminal phase, Dose/AUC_inf_* *λ*_z_) were calculated using the noncompartmental analysis tool of Phoenix WinNonLin v.8.01 (Certara/Pharsight; RRID: SCR_003163).

### Subcellular fractionation

SUM-159-KO + WT cell pellets were suspended in PBS + 0.5% BSA and subjected to sonication and sequential centrifugation (2,000*g* for 12 min, 15,000*g* for 60 min). The pellet following 2,000*g* centrifugation is the nuclear fraction, supernatant following 15,000*g* centrifugation is the ER fraction and the pellet following 15,000*g* centrifugation is the lysosome fraction. Enrichment for nuclear, ER and lysosomal fractions was validated by immunoblotting for histone H3, calreticulin and LAMP2, respectively.

### MS-based DIA analyses of whole-cell lysates

SUM-159 cells were treated with vehicle or ERX-41 (500 nM). After 6 h of treatment, cells were pelleted, snap-frozen and then lysed in buffer containing 5% SDS/50 mM triethylammonium bicarbonate (TEAB) in the presence of protease and phosphatase inhibitors (Halt, Thermo Scientific) and nuclease (Pierce Universal Nuclease for Cell Lysis, Thermo Scientific). Aliquots corresponding to 100 µg of protein (EZQ Protein Quantitation Kit, Thermo Scientific) were reduced with tris (2-carboxyethyl) phosphine hydrochloride, alkylated in the dark with iodoacetamide and applied to S-Traps (mini; Protifi) for tryptic digestion (sequencing grade; Promega) in 50 mM TEAB. Peptides were eluted from S-Traps with 0.2% formic acid in 50% aqueous acetonitrile and quantified using Pierce Quantitative Fluorometric Peptide Assay (Thermo Scientific). DIA–MS was conducted on an Orbitrap Fusion Lumos mass spectrometer (Thermo Scientific). Online HPLC separation was accomplished with an RSLC NANO HPLC system (Thermo Scientific/Dionex: column, PicoFrit (New Objective; 75 μm i.d.) packed to 15 cm with C18 adsorbent (Vydac; 218MS 5 μm, 300 Å); mobile phase A, 0.5% acetic acid (HAc)/0.005% trifluoroacetic acid (TFA) in water; mobile phase B, 90% acetonitrile/0.5% HAc/0.005% TFA/9.5% water; gradient 3 to 42% B in 120 min; flow rate, 0.4 μl min^–1^. A pool was made of the six samples (three replicates from each group), and 2-µg peptide aliquots were analyzed using gas-phase fractionation and 4-*m/z* windows (30,000 resolution for precursor and product ion scans, all in the Orbitrap) to create a DIA chromatogram library^33^ by searching against the Prosit-generated predicted spectral library^34^ based on the UniProt_human_20191022 protein sequence database. Experimental samples were blocked by replicate and randomized within each replicate for sample preparation and analysis; injections of 2 µg of peptides and a 2-h HPLC gradient were employed. MS data for experimental samples were acquired in the Orbitrap using 8-*m/z* windows (staggered; 30,000 resolution for precursor and product ion scans) and searched against the chromatogram library. Scaffold DIA (v.3.1.0, Proteome Software) was used for all DIA–MS data processing. GO analysis of differentially expressed proteins was conducted using biological processes and the cellular component, focusing on the group that exhibited ≥1.5-fold change (FC) comparing vehicle and ERX-41.

### Statistics and reproducibility

Statistical differences between groups were analyzed with either a *t*-test or analysis of variance (ANOVA), as appropriate, using GraphPad Prism 9 software (RRID: SCR_002798). All data represented in plots represent mean ± s.e.m. or mean ± s.d. Data distribution was assumed to be normal, but this was not formally tested. All in vitro assays were performed in biological replicates. No data were excluded and in vitro experiments were not randomized. A value of *P* < 0.05 was considered statistically significant. For electron microscopy (Fig. 3g,h), in confocal microscopy studies (Figs. 3i,j and 5g,h) and immunofluorescence (Extended Data Fig. 6a,d) where representative images were used, at least two independent experiments were performed with similar results. Tissue microarray staining (Fig. 6a) and mouse organ tissue staining (Extended Data Figs. 2a,d and 7d) were performed once. IHC staining studies (Fig. 6i and Extended Data Fig. 3i) were performed in multiple samples. For blots/gels with representative images (Figs. 3m–o, 5k,l, 7a,j,m,q,r and 8n and Extended Data Figs. 3e–h,l, 5e, 6e,f, 7c, 8b, 9f,g,j.k and 10a,b), each experiment was performed at least twice with similar results. The data shown in Extended Data Fig. 4d–h were performed once only.

For animal studies, sample size of tumors/treatment was derived using effect information from previous studies, and calculations were based on a model of unpaired data power of 0.8, *P* < 0.05. Xenograft tumor experiments were randomized. Data collection and analysis were not performed blind to the conditions of the experiments.

### Reporting summary

Further information on research design is available in the Nature Research Reporting Summary linked to this article.

### Supplementary information

Supplementary Information Synthetic schema of ERX-41, ERX-11-9, ERX-11-16, ERX-11-30 and ERX-44, along with their characterization by NMR.

Reporting Summary

Supplementary Tables Supplementary Table 1, cell lines; Supplementary Table 2, antibodies; Supplementary Table 3, primers.

Supplementary Video 1 Live-cell imaging of MDA-MB-231 cells with vehicle or ERX-41 treatment.

Supplementary Video 2 Live-cell imaging of HMEC cells with vehicle or ERX-41 treatment.

Supplementary Video 3 Live-cell imaging of SUM-159 cells with vehicle or ERX-41 treatment.

### Source data

Source Data Fig. 1 Numerical source data.

Source Data Fig. 2 Numerical source data.

Source Data Fig. 3 Numerical source data.

Source Data Fig. 3 Uncropped blots and gels.

Source Data Fig. 4 Numerical source data.

Source Data Fig. 5 Numerical source data.

Source Data Fig. 5 Uncropped blots and gels.

Source Data Fig. 6 Numerical source data.

Source Data Fig. 6 Uncropped blots and gels.

Source Data Fig. 7 Numerical Source Data.

Source Data Fig. 7 Uncropped blots and gels.

Source Data Fig. 8 Numerical source data.

Source Data Fig. 8 Uncropped blots and gels.

Source Data Extended Data Fig. 1 Numerical source data.

Source Data Extended Data Fig. 2 Numerical source data.

Source Data Extended Data Fig. 3 Numerical source data.

Source Data Extended Data Fig. 3 Uncropped blots, gels and images.

Source Data Extended Data Fig. 4 Numerical source data.

Source Data Extended Data Fig. 4 Uncropped blots.

Source Data Extended Data Fig. 5 Numerical source data.

Source Data Extended Data Fig. 5 Uncropped blots.

Source Data Extended Data Fig. 6 Uncropped blots.

Source Data Extended Data Fig. 7 Uncropped blots.

Source Data Extended Data Fig. 8 Numerical source data.

Source Data Extended Data Fig. 8 Uncropped blots and gels.

Source Data Extended Data Fig. 9 Numerical source data.

Source Data Extended Data Fig. 9 Uncropped blots and gels.

Source Data Extended Data Fig. 10 Numerical source data.

Source Data Extended Data Fig. 10 Uncropped blots and gels.

## Data Availability

mRNA-seq data are available from NCBI GEO under accession no. GSE168800. Proteomics data are available from ProteomeXchange (accession no. PXD032693) and MassIVE (accession no. MSV000089091). Source data are provided with this paper. Data that support the findings of this study are available from the corresponding authors upon request.
