# Author Correction: Targeting LIPA independent of its lipase activity is a therapeutic strategy in solid tumors via induction of endoplasmic reticulum stress

**DOI:** 10.1038/s43018-024-00783-4

**Published:** 2024-05-20

**Authors:** Xihui Liu, Suryavathi Viswanadhapalli, Shourya Kumar, Tae-Kyung Lee, Andrew Moore, Shihong Ma, Liping Chen, Michael Hsieh, Mengxing Li, Gangadhara R. Sareddy, Karla Parra, Eliot B. Blatt, Tanner C. Reese, Yuting Zhao, Annabel Chang, Hui Yan, Zhenming Xu, Uday P. Pratap, Zexuan Liu, Carlos M. Roggero, Zhenqiu Tan, Susan T. Weintraub, Yan Peng, Rajeshwar R. Tekmal, Carlos L. Arteaga, Jennifer Lippincott-Schwartz, Ratna K. Vadlamudi, Jung-Mo Ahn, Ganesh V. Raj

**Affiliations:** 1 Department of Urology, University of Texas Southwestern Medical Center at Dallas, Dallas, TX USA; 2 Department of Obstetrics and Gynecology, University of Texas Health Science Center at San Antonio, San Antonio, TX USA; 3 CDP program, Mays Cancer Center, University of Texas Health Science Center at San Antonio, San Antonio, TX USA; 4 Department of Chemistry and Biochemistry, University of Texas at Dallas, Richardson, TX USA; 5 Janelia Research Campus, Howard Hughes Medical Institute, Ashburn, VA USA; 6 Institute of Future Agriculture, Northwest A&F University, Yangling, China; 7 Department of Microbiology, Immunology and Molecular Genetics, The Joe R & Teresa Lozano Long School of Medicine, University of Texas Health Science Center at San Antonio, San Antonio, TX USA; 8 Fujian Provincial Key Laboratory of Neurodegenerative Disease and Aging Research, Institute of Neuroscience, Medical College, Xiamen University, Xiamen, China; 9 Department of Biochemistry and Structural Biology, University of Texas Health Science Center at San Antonio, San Antonio, TX USA; 10 Department of Pathology, University of Texas Southwestern Medical Center at Dallas, Dallas, TX USA; 11 Simmons Cancer Center, University of Texas Southwestern Medical Center at Dallas, Dallas, TX USA; 12 Audie L. Murphy Division, South Texas Veterans Health Care System, San Antonio, TX USA; 13 Department of Pharmacology, University of Texas Southwestern Medical Center at Dallas, Dallas, TX USA

**Keywords:** Drug development, Target identification, Cancer therapy, Cancer

Correction to: *Nature Cancer* 10.1038/s43018-022-00389-8, published online 2 June 2022

In the version of this article initially published, in the legend to Extended Data Fig. 2a, the dose of ERX-41 was reported as 10 mg/kg/day for both BALB/c and nude mice, but the BALB/c mice were in fact administered 20 mg/kg/day. The correct figure legend for Extended Data Fig. 2a is “Nude mice with MDA-MB-231 xenograft tumors were treated with either a vehicle or ERX-41 (10 mg/kg/day) for a duration of 52 days. Similarly, BALB/c mice with D2A1 xenograft tumors received a treatment of 20 mg/kg/day for 23 days. Following these treatments, histologic architecture in various organs of both the nude mice and BALB/c mice was assessed using H&E staining (a).” This has been corrected in the HTML and PDF versions of the article.

