# Supplementary material for: Targeting LIPA independent of its lipase activity is a therapeutic strategy in solid tumors via induction of endoplasmic reticulum stress
Source: Nat Cancer. 2022 Jun 2;3(7):866–84. doi: 10.1038/s43018-022-00389-8 (PMC9325671; doi:10.1038/s43018-022-00389-8)

nature portfolio

https://doi.org/10.1038/s43018-022-00389-8

Supplementary information

Targeting LIPA independent of its lipase activity is a therapeutic strategy in solid tumors via induction of endoplasmic reticulum stress

In the format provided by the authors and unedited

Chemicals and synthetic procedure

All chemical reagents and solvents were obtained from commercial sources and used without additional purification. ^{1}H and ^{13}C nuclear magnetic resonance (NMR) spectra were recorded on a Bruker Avance III HD 600 MHz NMR NMR spectrometer. Chemical shifts are reported in parts per million (δ) from an internal standard of residual DMSO-d6 (2.50 or 39.5 ppm). Data are reported as follows: chemical shift (δ), multiplicity (s, singlet; d, doublet; dd, doublet of doublet; t, triplet; q, quartet; br s, broad singlet; m, multiplet), coupling constant (J) in Hertz (Hz), integration. High resolution mass spectrometry (HRMS) data were obtained on a Shimadzu LCMS-IT-TOF using electrospray ionization (ESI). Mass spectra (MS) were recorded on a Shimadzu AXIMA Confidence MALDI-TOF mass spectrometer (nitrogen UV laser, 50 Hz, 337 nm) by using α-cyano-4-hydroxycinnamic acid (CHCA) as a matrix.

Scheme S1. Synthesis of ERX-41

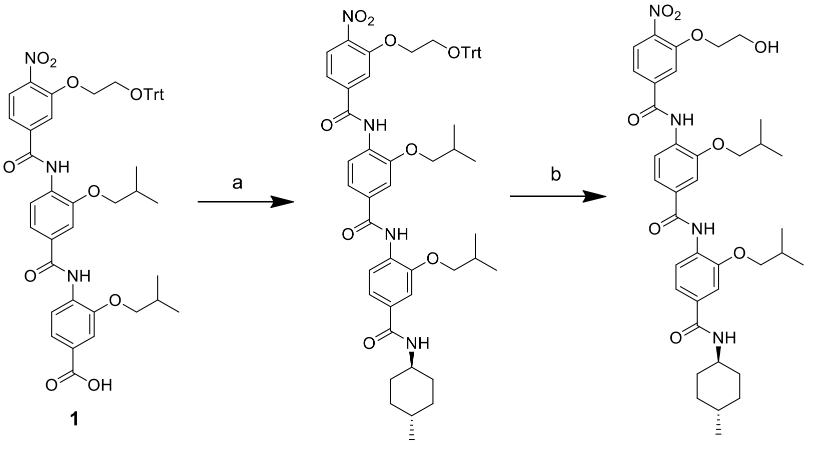

Reagents and conditions: (a) trans-4-methylcyclohexylamine, HATU, DIEA, DMF, rt, 24 h; (b) conc. HCl, THF, rt, 24 h.

Compound **1**

The title compound was prepared following published procedure [1].

ERX-41

Trans-4-methylcyclohexylamine (0.73 g, 6.4 mmol) was added to a solution of compound **1** (2.7 g, 3.2 mmol), HATU (1.4 g, 3.7 mmol), and DIEA (1.2 mL, 6.9 mmol) in DMF (30 mL). The reaction mixture was stirred at room temperature for 24 h and then diluted with EtOAc (200 mL) and 0.5 N HCl (100 mL). The layers were separated, and the aqueous layer was extracted with EtOAc (100 mL). The organic layers were combined, washed with 0.5 N HCl and brine, dried over anhydrous sodium sulfate, and concentrated under reduced

pressure. The resulting solid was washed with EtOAc and dried in vacuo to give compound **2** as a white sold (1.75 g). The product was used in the next reaction without further purification. Concentrated HCl (30 mL) was added to a solution of compound **2** (1.75 g) and THF (300 mL). The reaction mixture was stirred at room temperature for 24 h and then concentrated under reduced pressure. The resulting solid was washed with MeOH and dried in vacuo to give **ERX-41** as a light yellow solid (1.3 g, 57% yield over 2 reaction steps). ^{1}H NMR (DMSO-d_6, 600 MHz): δ 9.85 (br s, 1 H), 9.45 (br s, 1 H), 8.15 (d, J = 7.7 Hz, 1 H), 8.02 (d, J = 8.4 Hz, 1 H), 7.96 (d, J = 8.6 Hz, 1 H), 7.92 (d, J = 8.2 Hz, 1 H), 7.85 (s, 1 H), 7.64 (s, 1 H), 7.63 (d, J = 8.2 Hz, 1 H), 7.60 (d, J = 8.4 Hz, 1 H), 7.52 (s, 1 H), 7.51 (d, J = 6.2 Hz, 1 H), 4.96 (t, J = 5.2 Hz, 1 H), 4.29 (t, J = 5.0 Hz, 2 H), 3.91 (d, J = 5.9 Hz, 2 H), 3.90 (d, J = 5.9 Hz, 2 H), 3.76 (q, J = 5.1 Hz, 2 H), 3.75–3.71 (m, 1 H), 2.15–2.07 (m, 2 H), 1.85–1.82 (m, 2 H), 1.73–1.70 (m, 2 H), 1.40–1.31 (m, 3 H), 1.05–1.03 (m, 1 H, overlapped with the signal of CH(C$\underline{H}_3$)$_2$), 1.03 (d, J = 6.6 Hz, 6 H), 1.01–1.00 (m, 1 H, overlapped with the signal of CH(C$\underline{H}_3$)$_2$), 1.01 (d, J = 6.6 Hz, 6 H), 0.90 (d, J = 6.6 Hz, 3 H). ^{13}C NMR (DMSO-d_6, 150 MHz): δ 164.7, 164.2, 163.6, 151.1, 150.8, 149.8, 141.4, 139.2, 131.9, 131.6, 129.8, 129.6, 125.0, 124.0, 122.2, 119.73, 119.66, 119.59, 114.2, 111.1, 110.9, 74.6, 74.5, 71.5, 59.2, 48.4, 33.8, 32.3, 31.6, 27.9, 27.8, 22.2, 19.13, 19.05. HRMS-ESI (m/z): [M-H]$^-$ calcd for $C_{38}H_{47}N_4O_9$: 703.3349, found 703.3331.

Scheme S2. Synthesis of **ERX-11-9**, **ERX-11-16**, **ERX-11-30**, and **ERX-44**

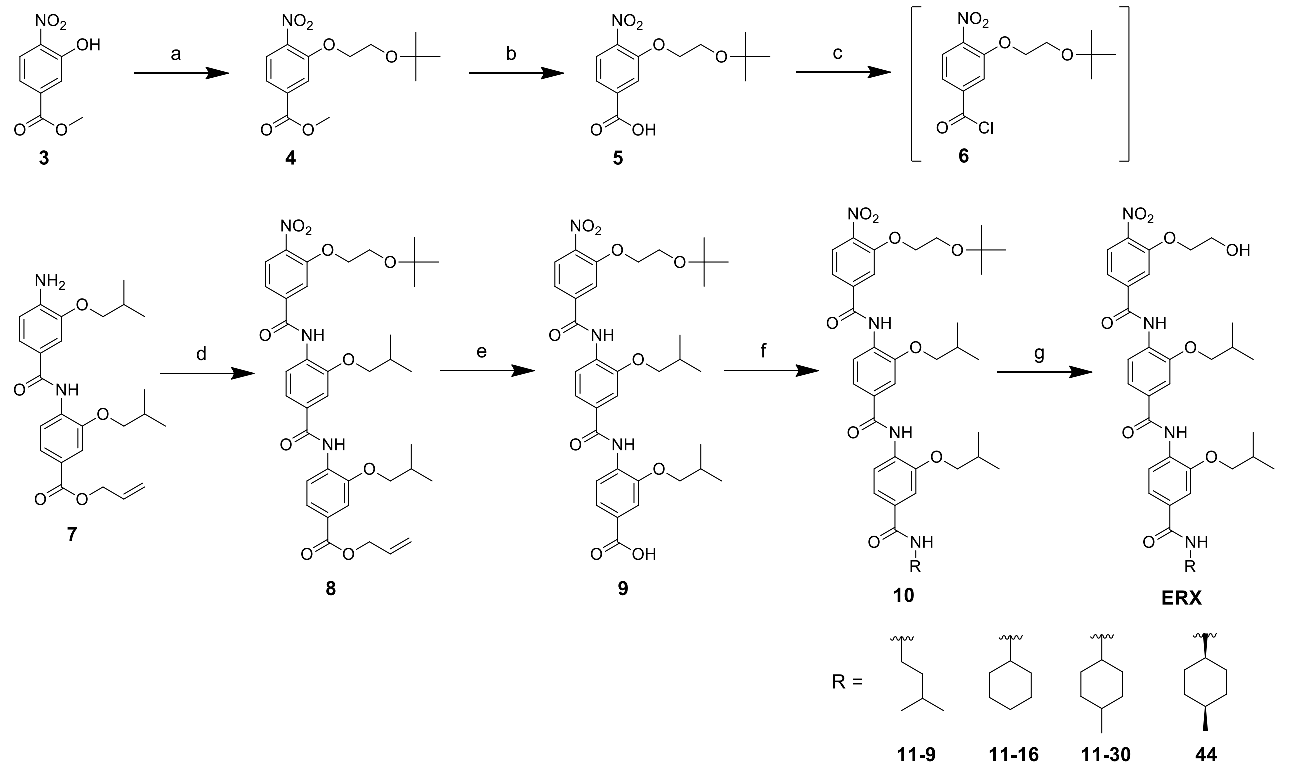

Reagents and conditions: (a) 2-tert-butoxyethyl methanesulfonate, K_2CO_3, DMF, 80 °C, 12 h; (b) NaOH, H_2O,

THF, MeOH, rt, 12 h; (c) $(COCl)_2$, cat. DMF, DCM, rt, 2 h; (d) **6**, DIEA, DCM, rt, 24 h; (e) $Pd(PPh_3)_4$, $PhSiH_3$, THF, rt, 1 h; (f) R-NH_2, HATU, DIEA, DMF, rt, 24 h; (b) TFA, rt, 1 h, then, LiOH, DMF, rt, 12 h.

Compound **3**

The title compound was prepared following published procedure [1].

Compound **5**

To a solution of compound **3** (5.25 g, 26.6 mmol) and K_2CO_3 (7.38 g, 53.4 mmol) in DMF (300 mL) was added 2-tert-butoxyethyl methanesulfonate (6.80 g, 34.6 mmol). The reaction mixture was heated to 80 °C for 12 h. After cooling to room temperature, the reaction mixture was concentrated under reduced pressure. The resulting residue was then diluted with EtOAc (200 mL) and brine (200 mL). The organic layer was separated, washed with brine (100 mL × 2), and concentrated under reduced pressure to give compound **4** as a yellow liquid (7.95 g), which was used in the next reaction without further purification. To a solution of compound **4** in THF (100 mL) and MeOH (100 mL) was added 10% aqueous NaOH solution (40 mL), and the reaction mixture was stirred at room temperature for 12 h. The reaction mixture was then concentrated to a volume of 80 mL under reduced pressure and washed with hexanes (80 mL). The resulting residues was acidified to pH 1-2 with 1 N HCl and extracted with EtOAc (200 mL). The organic layer was washed with brine (100 mL × 2) and concentrated under reduced pressure to give compound **5** as a light yellow solid (6.9 g, 92% yield over 2 reaction steps). ^{1}H NMR (DMSO-d_6, 600 MHz): δ 13.59 (br s, 1 H), 7.93 (d, J = 8.4 Hz, 1 H), 7.84 (d, J = 1.4 Hz, 1 H), 7.63 (dd, J = 8.4, 1.4 Hz, 1 H), 4.30–4.29 (m, 2 H), 3.65–3.63 (m, 2 H), 1.12 (s, 9 H), ^{13}C NMR (DMSO-d_6, 150 MHz): δ 165.8, 150.9, 142.4, 135.4, 124.7, 121.4, 116.2, 72.7, 69.8, 60.0, 27.2. HRMS-ESI (m/z): [M-H]$^-$ calcd for $C_{13}H_{16}NO_6$: 282.0983, found 282.0972.

Compound **7**

The title compound was prepared following published procedure [1].

Compound **8**

Oxalyl chloride (2.25 mL, 26.0 mmol) and two drops of anhydrous DMF were slowly added to a suspension of compound **5** (3.68 g, 13.0 mmol) in DCM (100 mL). The reaction mixture was stirred at room temperature for 2 h, and the solvent and excess oxalyl chloride were removed under reduced pressure. The resulting acyl chloride **6** was then dissolved in DCM (20 mL) and slowly added to a solution of compound **7** (4.40 g, 10.0 mmol) and DIEA (3.50 mL, 20.1 mmol) in DCM (50 mL). The reaction mixture was stirred at room temperature for 24 h and concentrated under reduced pressure. The resulting residue was then diluted with EtOAc (200 mL) and 1 N HCl (100 mL). The organic layer was separated, washed with saturated $NaHCO_3$ (100 mL) and brine (100 mL),

and concentrated under reduced pressure. The resulting solid was washed with EtOAc/hexanes (1:2, 40 mL × 3) and dried in vacuo to give compound **8** as a light yellow solid (6.6 g, 94% yield). ^{1}H NMR (DMSO-d_6, 600 MHz): δ 9.84 (br s, 1 H), 9.51 (br s, 1 H), 8.15 (d, J = 8.1 Hz, 1 H), 8.01 (d, J = 8.1 Hz, 1 H), 7.94 (d, J = 8.4 Hz, 1 H), 7.88 (d, J = 1.5 Hz, 1 H), 7.68 (dd, J = 8.4, 1.8 Hz, 1 H), 7.63 (d, J = 1.8 Hz, 1 H), 7.63 (dd, J = 8.1, 1.5 Hz, 1 H), 7.60 (dd, J = 8.4, 1.8 Hz, 1 H), 7.59 (d, J = 1.5 Hz, 1 H), 6.10–6.03 (m, 1 H), 5.42 (dd, J = 17.2, 1.5 Hz, 1 H), 5.29 (dd, J = 10.5, 1.3 Hz, 1 H), 4.82 (d, J = 5.5 Hz, 2 H), 4.34–4.33 (m, 2 H), 3.93 (d, J = 6.2 Hz, 2 H), 3.92 (d, J = 6.6 Hz, 2 H), 3.68–3.67 (m, 2 H), 2.17–2.08 (m, 2 H), 1.13 (s, 9 H), 1.04 (d, J = 6.6 Hz, 6 H), 1.01 (d, J = 6.6 Hz, 6 H). ^{13}C NMR (DMSO-d_6, 150 MHz): δ 165.0, 164.3, 163.6, 150.9, 150.8, 149.6, 141.5, 139.1, 132.7, 131.9, 131.7, 123.0 125.8, 124.9, 124.0, 122.1, 122.0, 119.8, 119.7, 118.0, 114.6, 112.1, 111.1, 74.6, 74.5, 72.8, 69.7, 65.1, 59.8, 27.82, 27.76, 27.2, 19.1. HRMS-ESI (*m/z*): [M-H]$^-$ calcd for $C_{38}H_{46}N_3O_{10}$: 704.3189, found 704.3207.

Compound **9**

$PhSiH_3$ (0.95 mL, 7.7 mmol) was added to a solution of compound **8** (2.70 g, 3.8 mmol) and $Pd(PPh_3)_4$ (443 mg, 0.38 mmol) in THF (100 mL), and the reaction mixture was stirred at room temperature for 2 h. The reaction mixture was then concentrated under reduced pressure. The resulting solid was washed with diethyl ether (50 mL × 3) and dried in vacuo to give compound **9** as a light yellow solid (2.3 g, 92% yield). ^{1}H NMR (DMSO-d_6, 600 MHz): δ 12.90 (br s, 1 H), 9.84 (br s, 1 H), 9.48 (br s, 1 H), 8.09 (d, J = 8.1 Hz, 1 H), 8.01 (d, J = 8.4 Hz, 1 H), 7.94 (d, J = 8.1 Hz, 1 H), 7.88 (s, 1 H), 7.63–7.57 (m, 4 H), 7.57 (s, 1 H), 4.33 (t, J = 4.6 Hz, 2 H), 3.92 (d, J = 6.2 Hz, 4 H), 3.67 (t, J = 4.6 Hz, 2 H), 2.16–2.08 (m, 2 H), 1.14 (s, 9 H), 1.03 (d, J = 6.6 Hz, 6 H), 1.01 (d, J = 6.6 Hz, 6 H). ^{13}C NMR (DMSO-d_6, 150 MHz): δ 166.9, 164.2, 163.6, 151.0, 150.8, 149.5, 141.5, 139.1, 131.7, 131.4, 129.9, 127.2, 124.9, 123.9, 122.1, 121.9, 119.8, 119.7, 114.6, 112.3, 111.0, 74.6, 74.5, 72.8, 69.7, 59.8, 27.80, 27.76, 27.2, 19.1. HRMS-ESI (*m/z*): [M-H]$^-$ calcd for $C_{35}H_{42}N_3O_{10}$: 664.2876, found 664.2864.

ERX-11-9

A solution of compound **9** (300 mg, 0.45 mmol), HATU (188 mg, 0.49 mmol), and DIEA (0.24 mL, 1.4 mmol) in DMF (10 mL) was stirred at room temperature for 30 min, and isoamylamine (118 mg, 1.4 mmol) was added to the solution. The reaction mixture was stirred at room temperature for 24 h and then diluted with EtOAc (100 mL) and 1 N HCl (100 mL). The organic layer was separated, washed with saturated $NaHCO_3$ (50 mL) and brine (50 mL), and concentrated under reduced pressure. The resulting solid was washed with EtOAc (20 mL × 3) and dried in vacuo to give compound **10-11-9** as a light yellow solid (230 mg, 70%). Compound **10-11-9** (230 mg, 0.31 mmol) was dissolved in TFA (5 mL) and the reaction mixture was stirred at room temperature for 1 h. The reaction mixture was concentrated under reduced pressure and precipitated by adding diethyl ether. The precipitate was filtered, washed with diethyl ether (10 mL × 3) and dried in vacuo to give a light yellow solid.

This product contained approximately 10% of the corresponding trifluoroacetate (data not shown). In order to hydrolyze the ester, this product was added to a solution of 1 N LiOH (1 mL) and DMF (20 mL), and stirred at room temperature for 12 h. The reaction mixture was then diluted with EtOAc (50 mL) and 1 N HCl (30 mL). The organic layer was separated, washed with brine (30 mL × 2), and concentrated under reduced pressure. The resulting solid was washed with EtOAc (10 mL × 3) and dried in vacuo to give **ERX-11-9** as a light yellow solid (110 mg, 52% yield). ^{1}H NMR (DMSO-d_6, 600 MHz): δ 9.85 (br s, 1 H), 9.46 (br s, 1 H), 8.40 (t, J = 5.5 Hz, 1 H), 8.02 (d, J = 8.1 Hz, 1 H), 7.96 (d, J = 8.4 Hz, 1 H), 7.93 (d, J = 8.1 Hz, 1 H), 7.85 (s, 1 H), 7.64 (s, 1 H), 7.63 (d, J = 8.4 Hz, 1 H), 7.60 (d, J = 8.1 Hz, 1 H), 7.53 (s, 1 H), 7.50 (d, J = 8.4 Hz, 1 H), 4.96 (br s, 1 H), 4.29 (t, J = 5.0 Hz, 2 H), 3.91 (d, J = 7.3 Hz, 2 H), 3.90 (d, J = 7.3 Hz, 2 H), 3.77–3.74 (m, 2 H), 3.31–3.28 (m, 2 H), 2.15–2.08 (m, 2 H), 1.66–1.59 (m, 1 H), 1.44 (q, J = 7.0 Hz, 2 H), 1.03 (d, J = 6.6 Hz, 6 H), 1.01 (d, J = 6.6 Hz, 6 H), 0.92 (d, J = 6.6 Hz, 6 H). ^{13}C NMR (DMSO-d_6, 150 MHz): δ 165.3, 164.2, 163.6, 151.1, 150.8, 149.9, 141.4, 139.2, 131.9, 131.5, 129.8, 129.6, 125.0, 124.0, 122.4, 119.8, 119.6, 119.5, 114.2, 111.1, 110.8, 74.6, 74.5, 71.5, 59.2, 38.2, 37.5, 27.9, 27.8, 25.4, 22.5, 19.11, 19.05. HRMS-ESI (m/z): [M-H]$^-$ calcd for $C_{36}H_{45}N_4O_9$: 677.3192, found 677.3161.

ERX-11-16

The title compound was prepared from compound **9** (300 mg, 0.45 mmol) and cyclohexylamine (134 mg, 1.4 mmol) following the procedure described for **ERX-11-9**, and obtained as a light yellow solid (140 mg, 45% yield over 2 reaction steps). ^{1}H NMR (DMSO-d_6, 600 MHz): δ 9.85 (br s, 1 H), 9.45 (br s, 1 H), 8.17 (d, J = 8.1 Hz, 1 H), 8.02 (d, J = 8.1 Hz, 1 H), 7.97 (d, J = 8.4 Hz, 1 H), 7.93 (d, J = 8.4 Hz, 1 H), 7.85 (s, 1 H), 7.64 (s, 1 H), 7.63 (d, J = 8.4 Hz, 1 H), 7.60 (d, J = 8.1 Hz, 1 H), 7.52 (s, 1 H), 7.52 (d, J = 6.2 Hz, 1 H), 4.96 (br s, 1 H), 4.29 (t, J = 5.0 Hz, 2 H), 3.92 (d, J = 5.7 Hz, 2 H), 3.91 (d, J = 5.7 Hz, 2 H), 3.80–3.76 (m, 1 H), 3.76 (t, J = 4.8 Hz, 2 H), 2.15–2.08 (m, 2 H), 1.85–1.81 (m, 2 H), 1.78–1.84 (m, 2 H), 1.64–1.52 (m, 1 H), 1.36–1.28 (m, 4 H), 1.17–1.12 (m, 1 H), 1.04 (d, J = 6.6 Hz, 6 H), 1.01 (d, J = 6.6 Hz, 6 H). ^{13}C NMR (DMSO-d_6, 150 MHz): δ 164.6, 164.2, 163.6, 151.1, 150.8, 149.8, 141.4, 139.2, 131.9, 131.6, 129.8, 129.6, 125.0, 124.0, 122.2, 119.73, 119.67, 119.6, 114.2, 111.1, 110.9, 74.6, 74.5, 71.5, 59.2, 48.4, 32.5, 27.9, 27.8, 25.3, 25.0, 19.13, 19.05. HRMS-ESI (m/z): [M-H]$^-$ calcd for $C_{37}H_{45}N_4O_9$: 689.3192, found 689.3175.

ERX-11-30

The title compound was prepared from compound **9** (300 mg, 0.45 mmol) and 4-methylcyclohexylamine (153 mg, 1.4 mmol) following the procedure described for **ERX-11-9**, and obtained as a light yellow solid (96 mg, 30% yield over 2 reaction steps). ^{1}H NMR (DMSO-d_6, 600 MHz): δ 9.85 (br s, 1 H), 9.46 (br s, 0.2 H), 9.45 (br s, 0.8 H), 8.15 (d, J = 8.1 Hz, 0.8 H), 8.06 (d, J = 7.7 Hz, 0.2 H), 8.02 (d, J = 8.1 Hz, 1 H), 7.96 (d, J = 8.4 Hz, 1

H), 7.92 (d, J = 8.4 Hz, 1 H), 7.85 (s, 1 H), 7.64 (s, 1 H), 7.63 (d, J = 8.4 Hz, 1 H), 7.60 (d, J = 8.4 Hz, 1 H), 7.52 (s, 0.8 H), 7.52 (d, J = 6.2 Hz, 1 H), 7.49 (s, 0.2 H), 4.96 (t, J = 5.1 Hz, 1 H), 4.29 (t, J = 5.0 Hz, 2 H), 3.91 (d, J = 5.9 Hz, 2 H), 3.90 (d, J = 5.9 Hz, 2 H), 3.76 (q, J = 4.8 Hz, 2 H), 3.76–3.70 (m, 1 H), 2.15–2.07 (m, 2 H), 1.86–1.82 (m, 1.6 H), 1.73–1.69 (m, 1.6 H), 1.72–1.65 (m, 0.6 H), 1.60–1.50 (m, 0.8 H), 1.48–1.42 (m, 0.4 H), 1.40–1.31 (m, 2.4 H), 1.05–1.03 (m, 0.8 H, overlapped with the signal of $CH(C\underline{H}_3)_2$), 1.03 (d, J = 6.6 Hz, 6 H), 1.01–1.00 (m, 0.8 H, overlapped with the signal of $CH(C\underline{H}_3)_2$), 1.01 (d, J = 6.6 Hz, 6 H), 0.96 (d, J = 7.0 Hz, 0.6 H), 0.90 (d, J = 6.6 Hz, 2.4 H). ^{13}C NMR (DMSO-d_6, 150 MHz): δ 165.1, 164.7, 164.2, 163.6, 151.1, 150.8, 149.82, 149.77, 141.4, 139.2, 131.9, 131.8, 131.6, 129.8, 129.6, 125.1, 124.0, 122.23, 122.19, 119.74, 119.67, 119.6, 114.2, 111.1, 110.9, 74.6, 74.5, 71.5, 59.2, 48.4, 47.0, 33.8, 32.3, 31.6, 29.9, 27.9, 27.83, 27.77, 22.2, 19.4, 19.14, 19.13, 19.06. HRMS-ESI (m/z): $[M-H]^-$ calcd for $C_{38}H_{47}N_4O_9$: 703.3349, found 703.3345.

ERX-44

The title compound was prepared from compound **9** (300 mg, 0.45 mmol) and cis-4-methylcyclohexylamine (153 mg, 1.4 mmol) following the procedure described for **ERX-11-9**, and obtained as a light yellow solid (110 mg, 35% yield over 2 reaction steps). ^1H NMR (DMSO-d_6, 600 MHz): δ 9.85 (br s, 1 H), 9.46 (br s, 1 H), 8.06 (d, J = 7.3 Hz, 1 H), 8.02 (d, J = 8.4 Hz, 1 H), 7.96 (d, J = 8.4 Hz, 1 H), 7.93 (d, J = 8.1 Hz, 1 H), 7.85 (s, 1 H), 7.84 (s, 1 H), 7.63 (d, J = 8.1 Hz, 1 H), 7.61 (d, J = 8.1 Hz, 1 H), 7.52 (d, J = 8.1 Hz, 1 H), 7.49 (s, 1 H), 4.96 (t, J = 5.1 Hz, 1 H), 4.29 (t, J = 5.0 Hz, 2 H), 3.92 (d, J = 6.2 Hz, 4 H), 3.91–3.86 (m, 1 H), 3.76 (q, J = 4.5 Hz, 2 H), 2.16–2.08 (m, 2 H), 1.72–1.65 (m, 3 H), 1.60–1.50 (m, 4 H), 1.48–1.42 (m, 2 H), 1.03 (d, J = 6.8 Hz, 6 H), 1.01 (d, J = 6.6 Hz, 6 H), 0.96 (d, J = 7.0 Hz, 3 H). ^{13}C NMR (DMSO-d_6, 150 MHz): δ 165.1, 164.2, 163.6, 151.1, 150.8, 149.9, 141.4, 139.2, 131.9, 131.8, 129.8, 129.6, 125.0, 124.0, 122.3, 119.74, 119.70, 119.6, 114.2, 111.1, 74.6, 74.5, 71.5, 59.2, 47.0, 29.9, 27.9, 27.81, 27.76, 19.4, 19.12, 19.05. HRMS-ESI (m/z): $[M-H]^-$ calcd for $C_{38}H_{47}N_4O_9$: 703.3349, found 703.3334.

^{1}H and ^{13}C NMR spectra of **ERX-41**

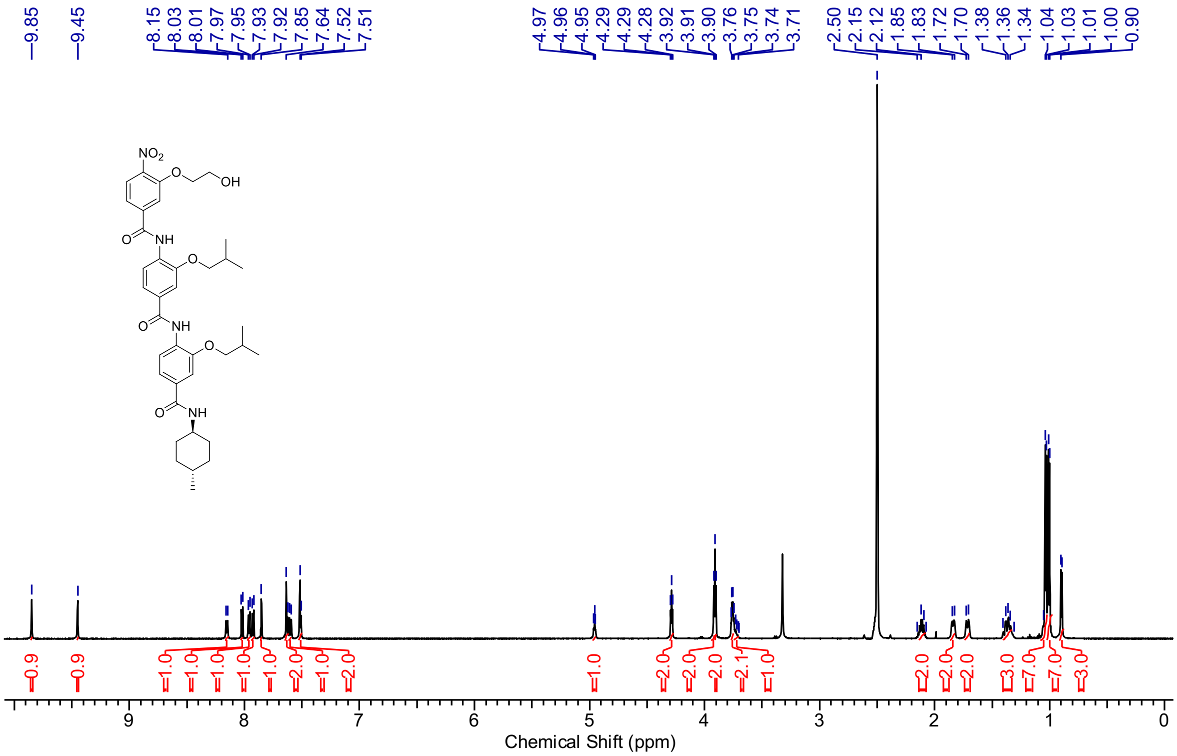

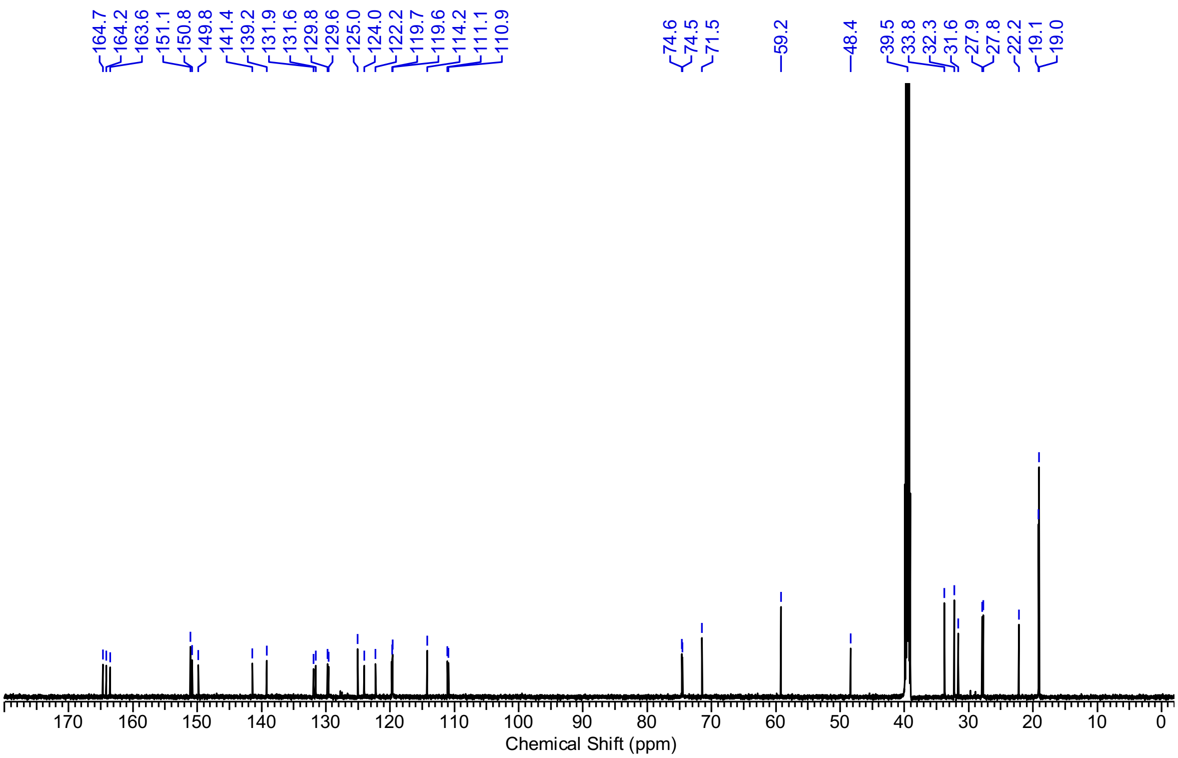

^{1}H and ^{13}C NMR spectra of compound **5**

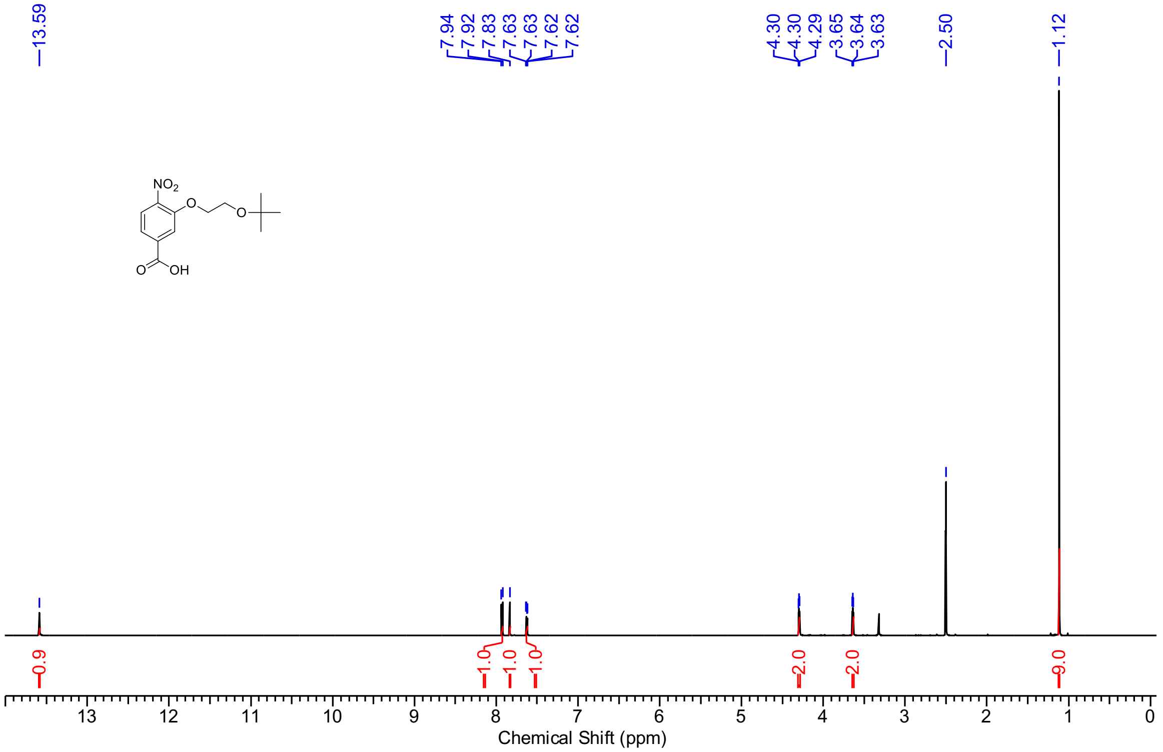

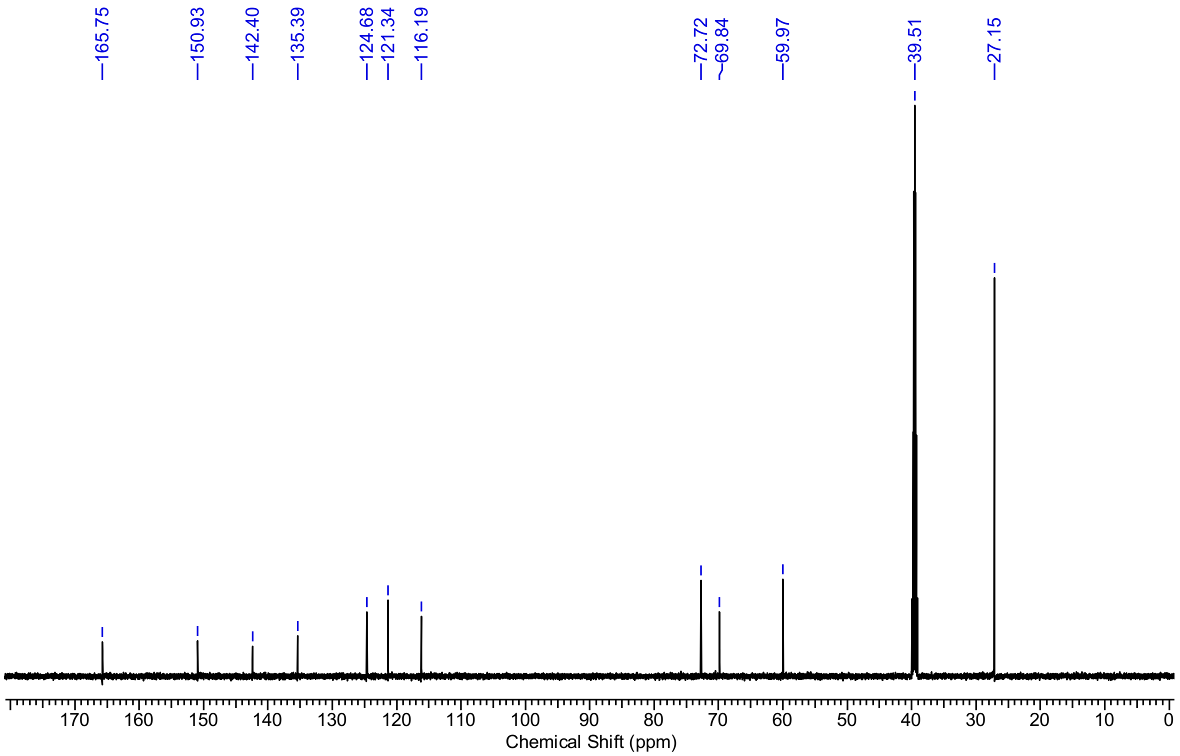

^{1}H and ^{13}C NMR spectra of compound **8**

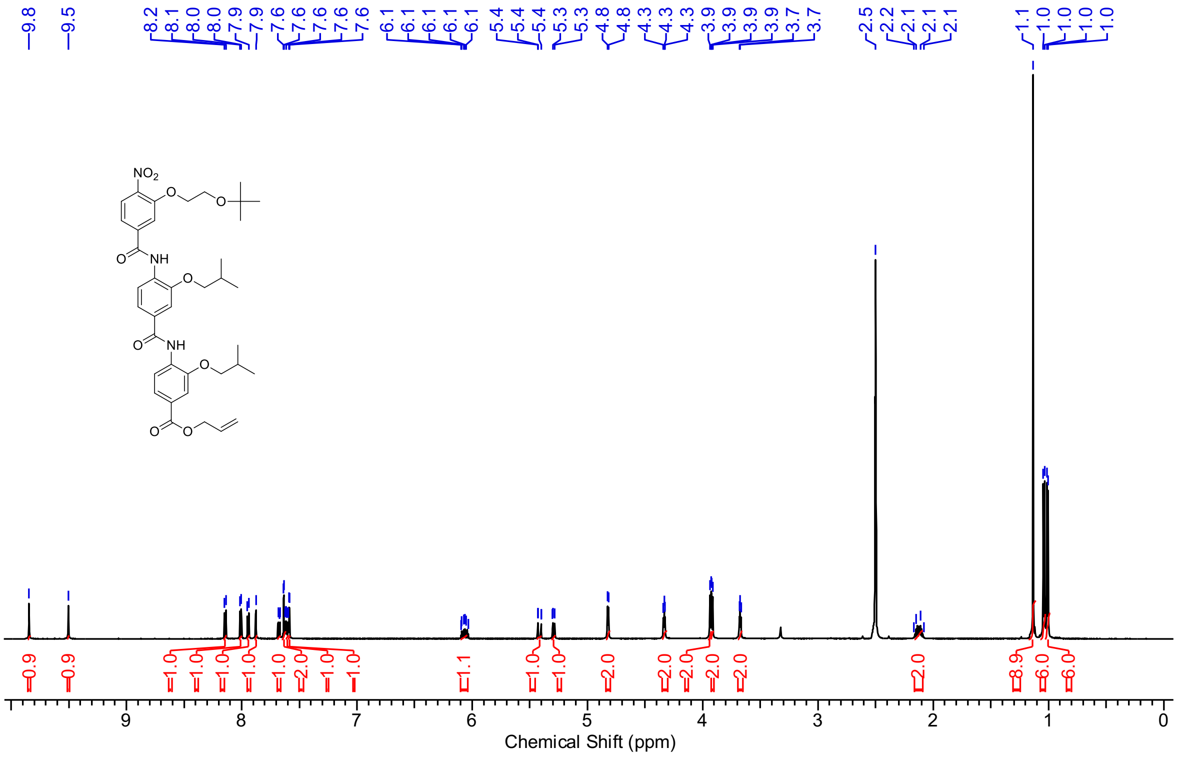

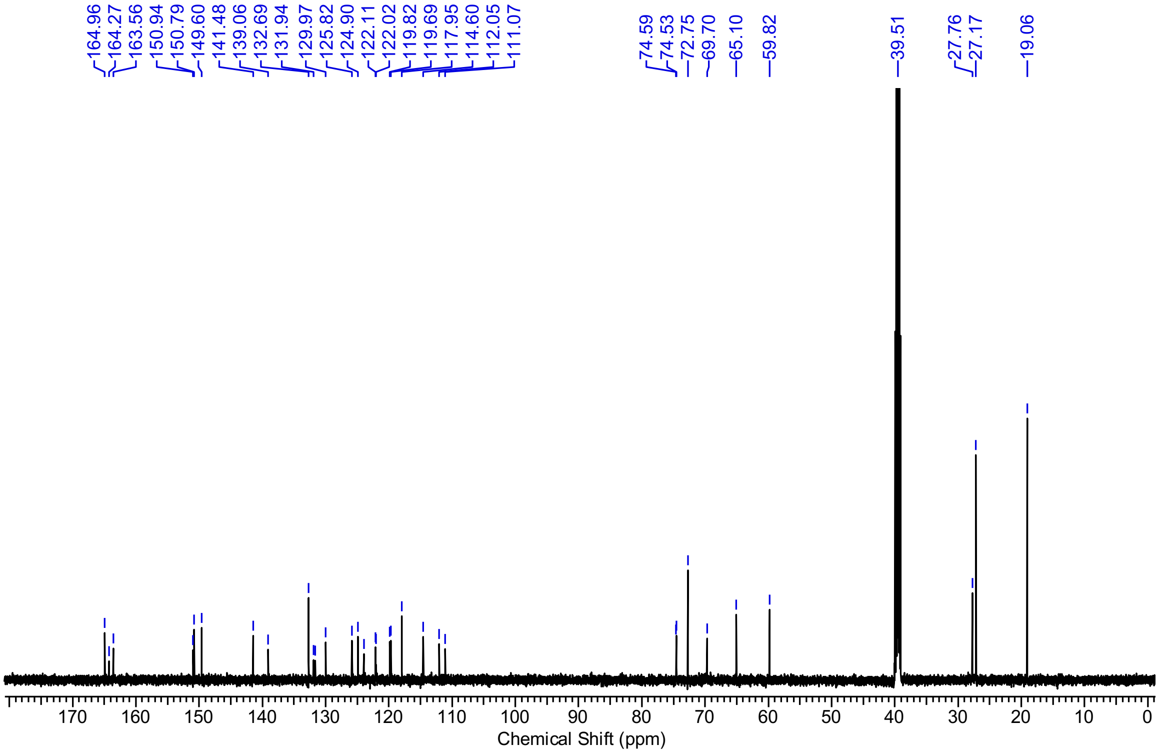

^1H and ^{13}C NMR spectra of compound **9**

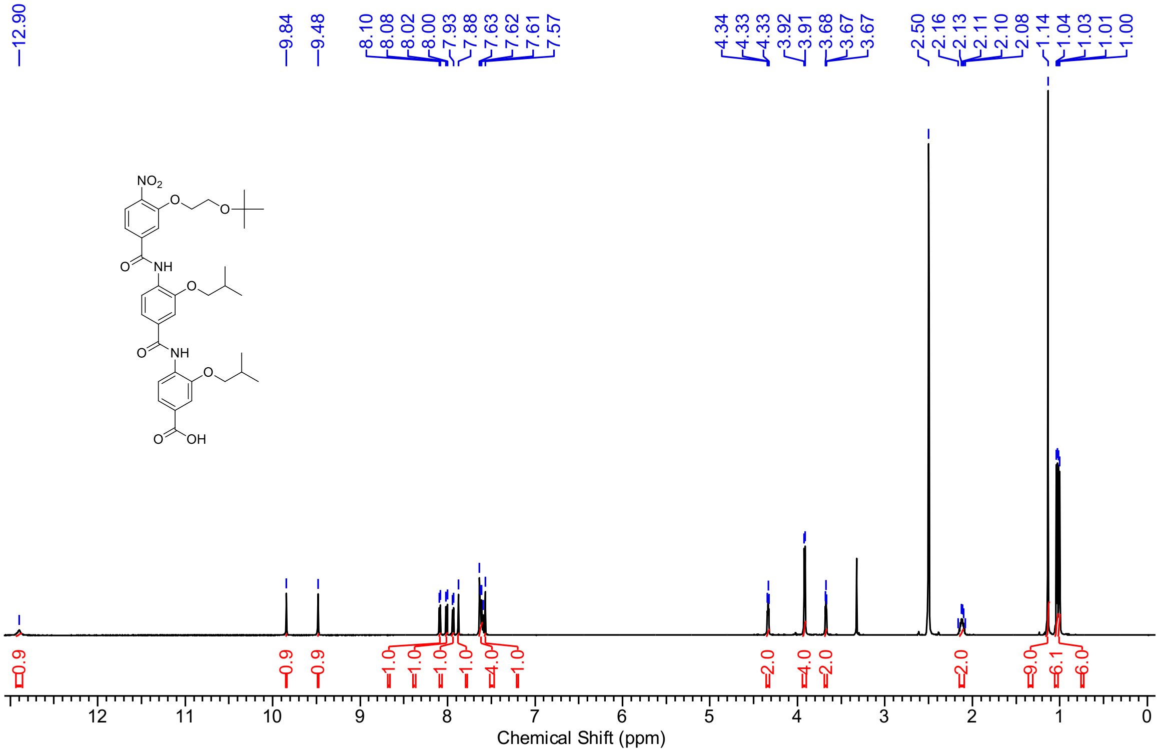

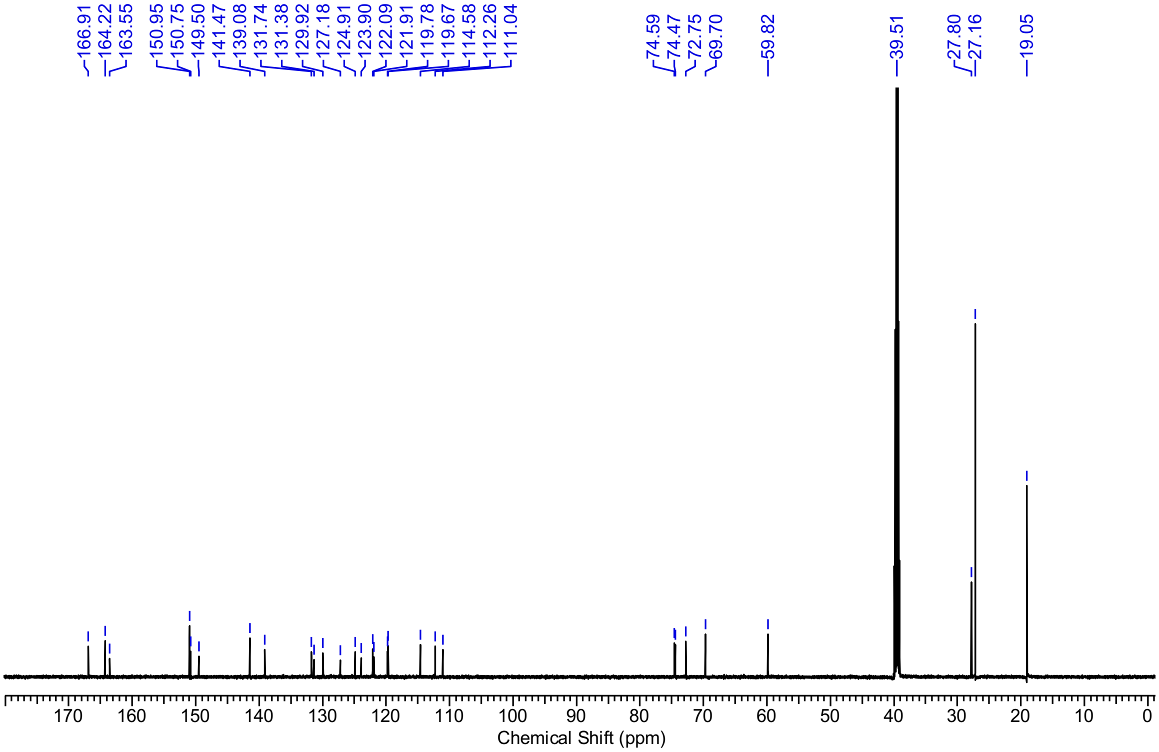

^1H and ^{13}C NMR spectra of **ERX-11-9**

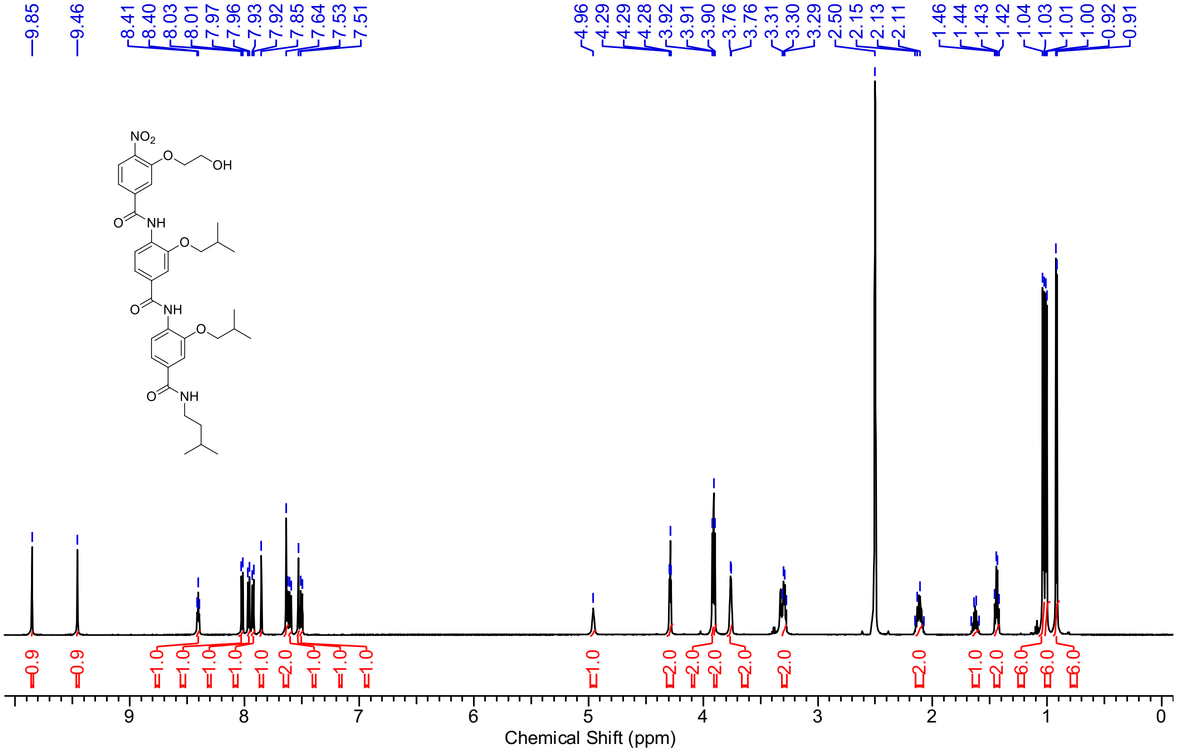

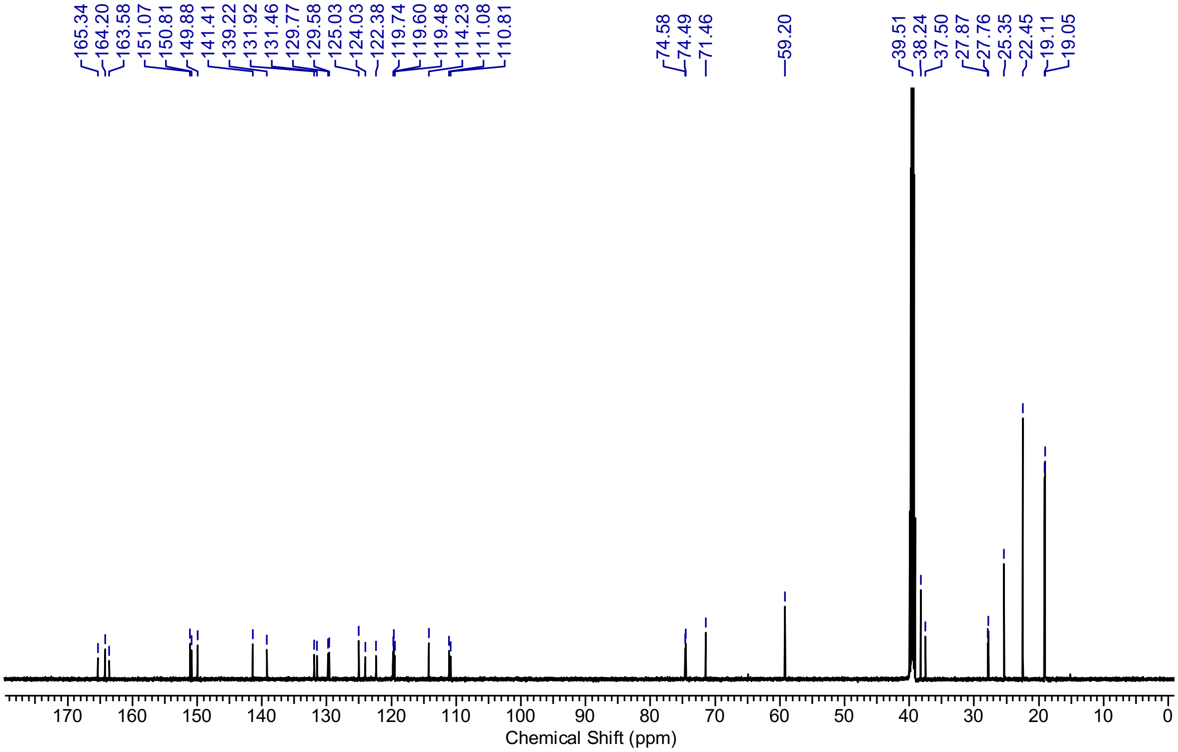

^{1}H and ^{13}C NMR spectra of **ERX-11-16**

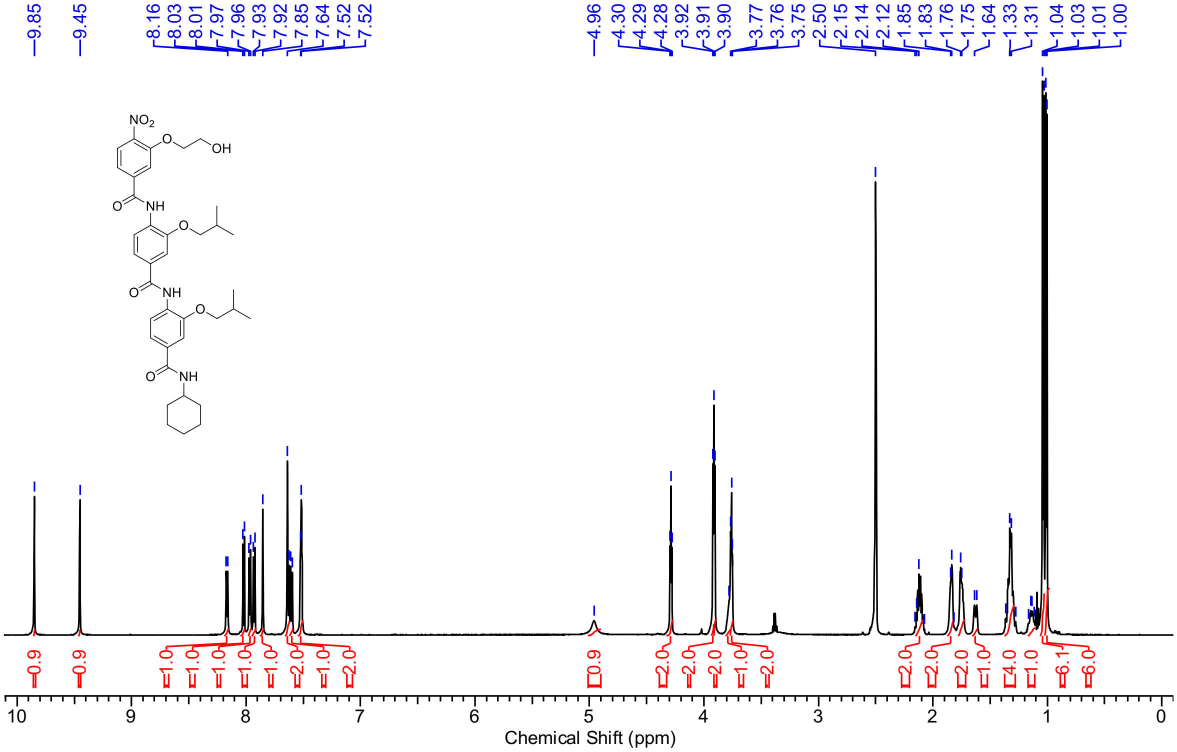

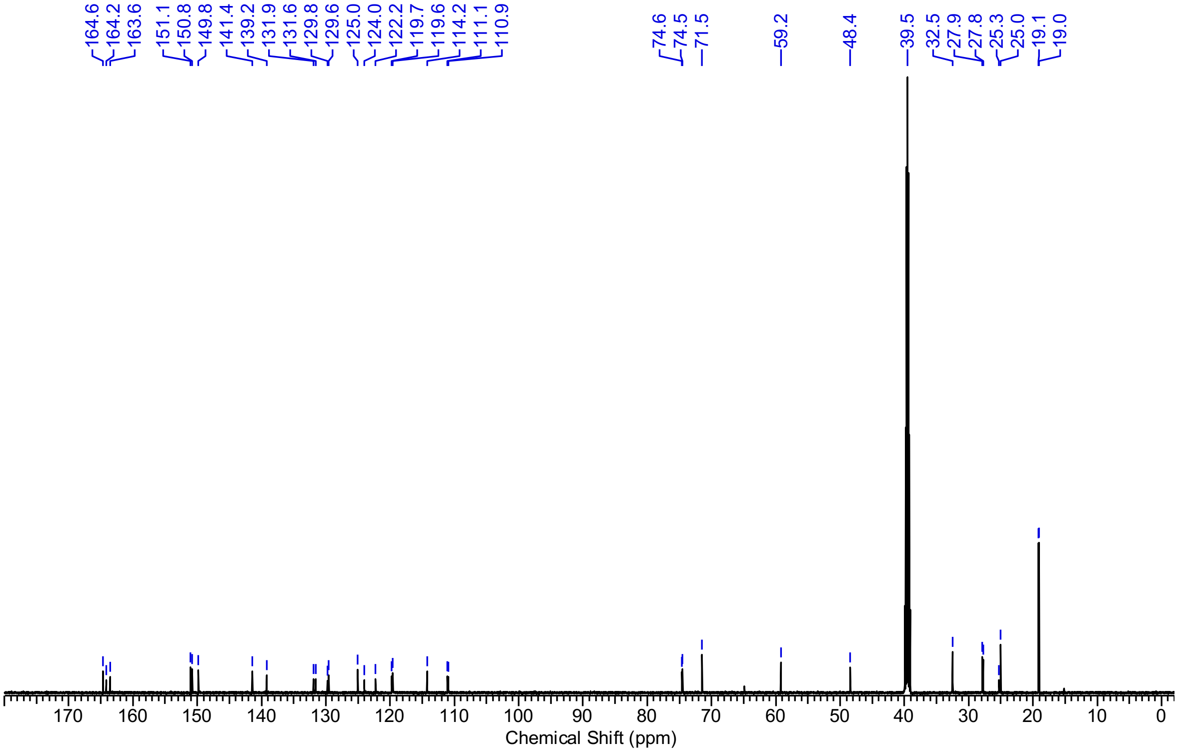

^{1}H and ^{13}C NMR spectra of **ERX-11-30**

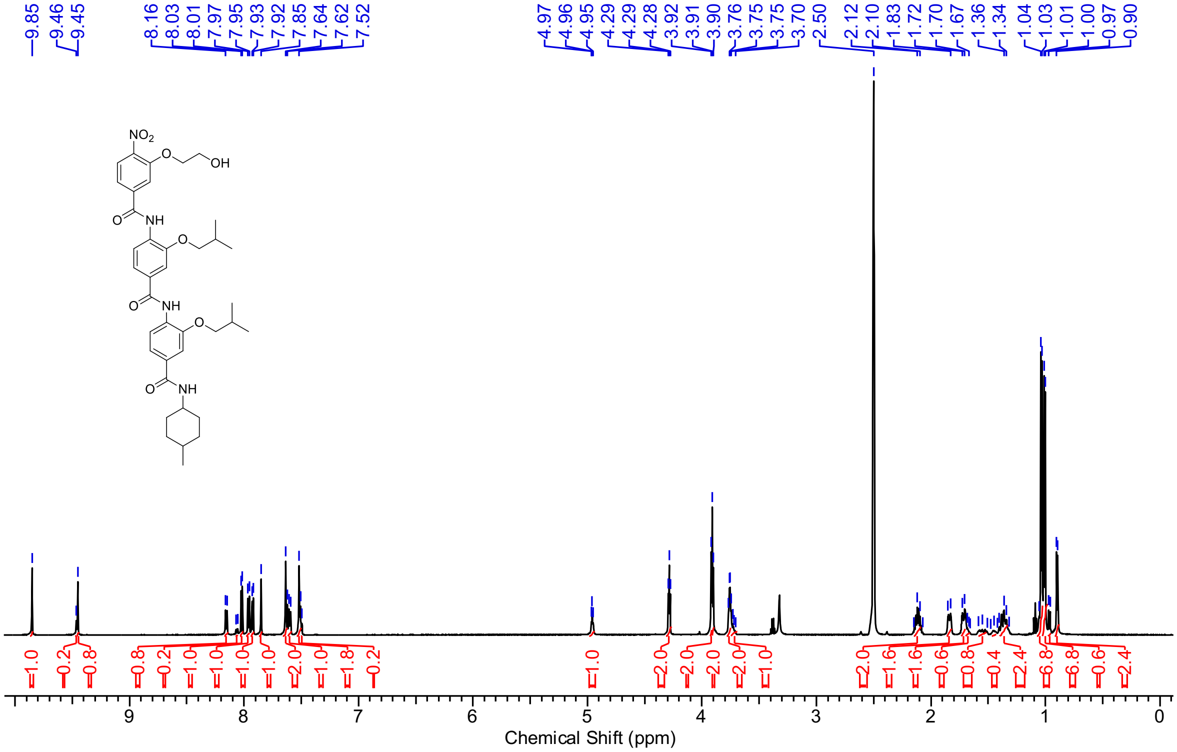

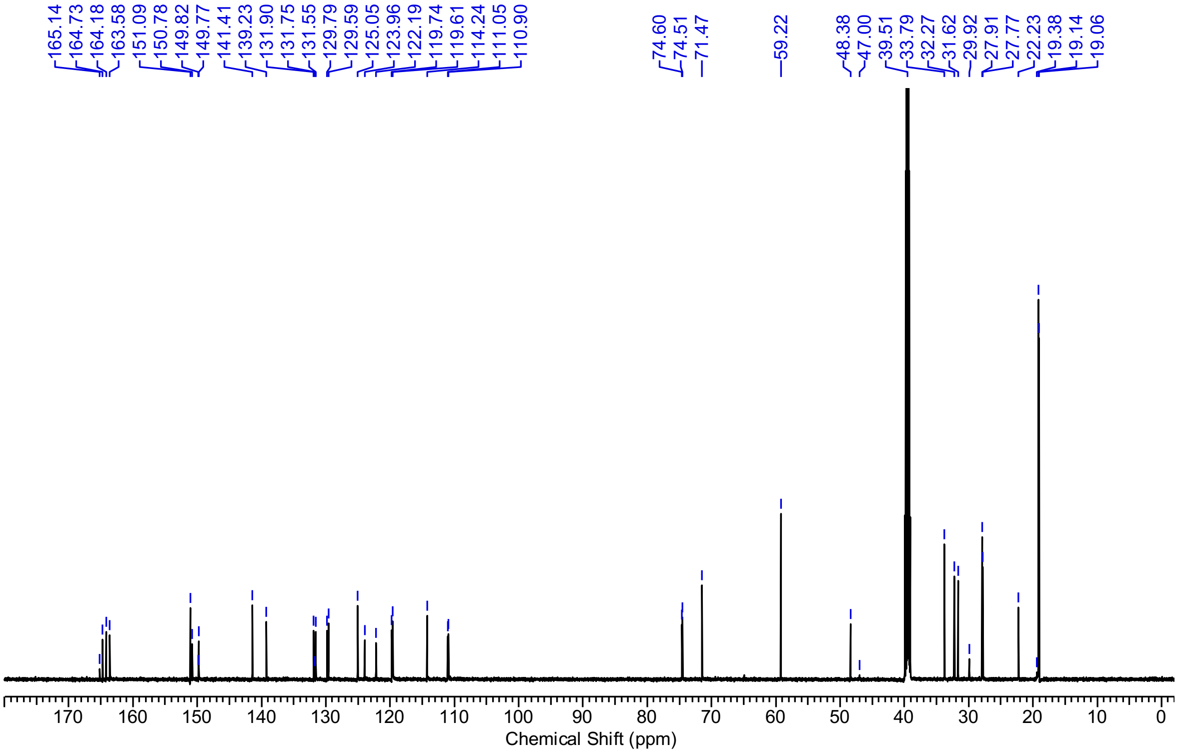

^{1}H and ^{13}C NMR spectra of **ERX-44**

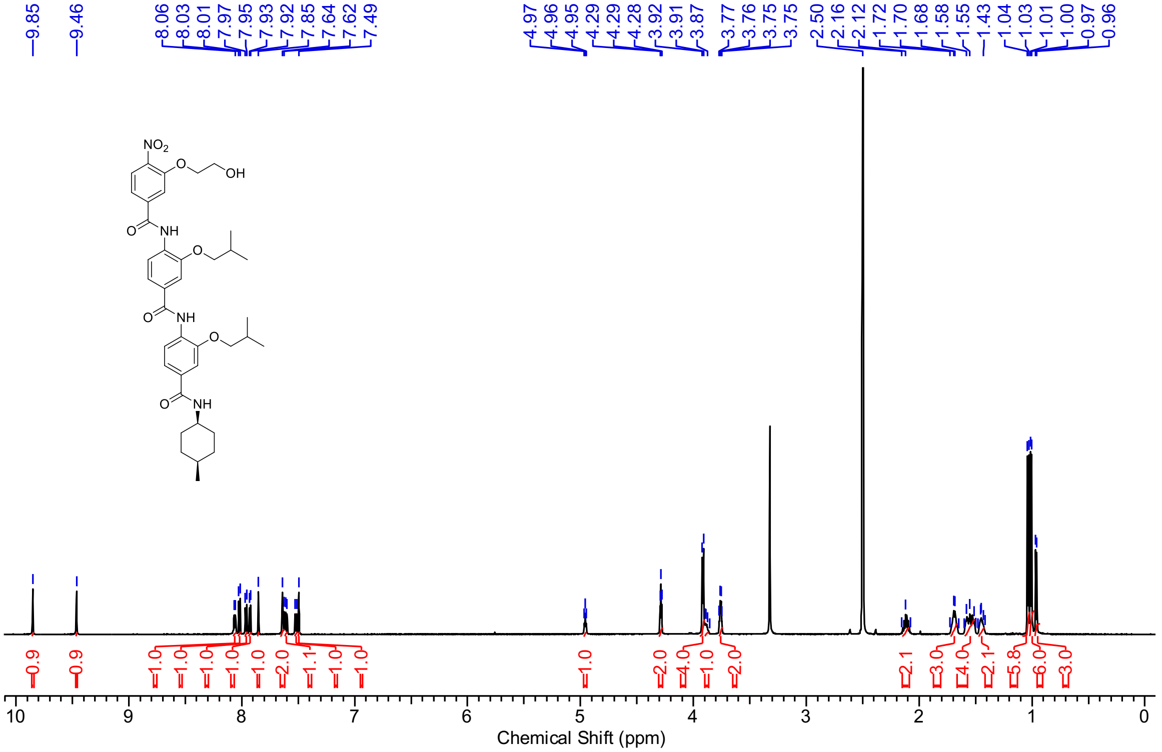

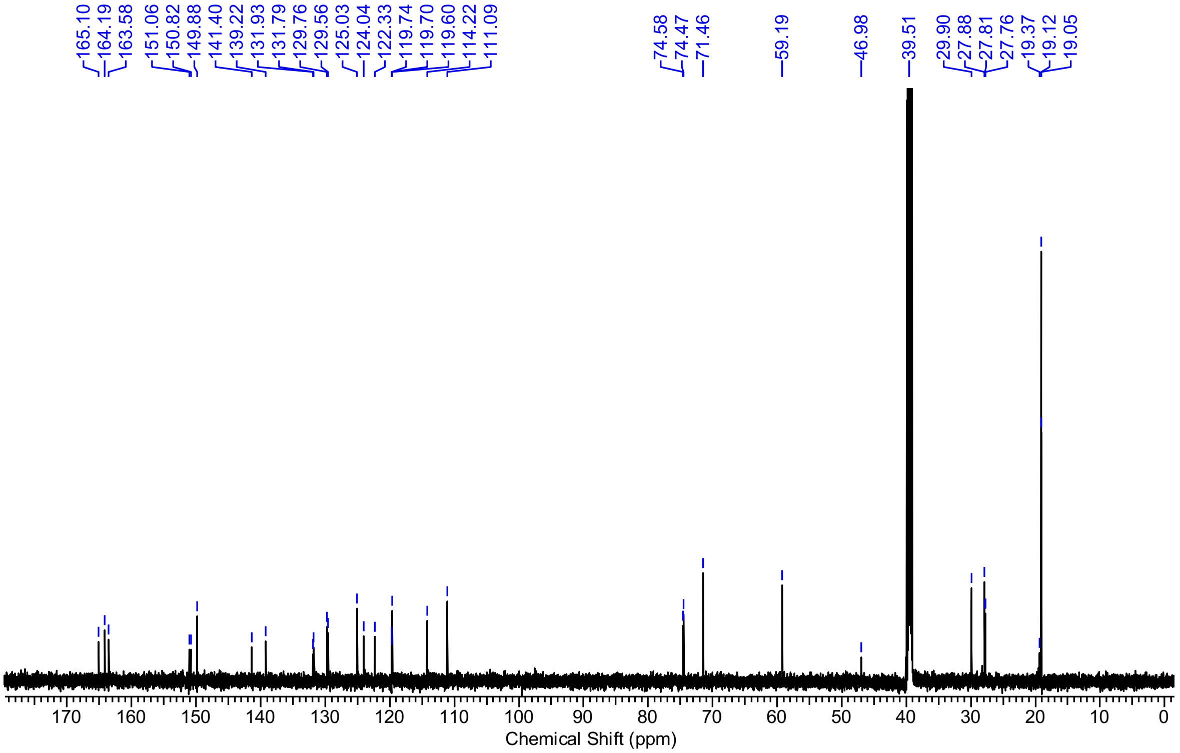

Supplement: Supplementary file 1 — Synthetic schema of ERX-41, ERX-11-9, ERX-11-16, ERX-11-30 and ERX-44, along with their characterization by NMR. [file 43018_2022_389_MOESM1_ESM.pdf]
