# Supplementary material for: Targeting LIPA independent of its lipase activity is a therapeutic strategy in solid tumors via induction of endoplasmic reticulum stress
Source: Nat Cancer. 2022 Jun 2;3(7):866–84. doi: 10.1038/s43018-022-00389-8 (PMC9325671; doi:10.1038/s43018-022-00389-8)
Supplement: Supplementary file 2 — Reporting Summary [file 43018_2022_389_MOESM2_ESM.pdf]

Corresponding author(s): Ratna K. Vadlamudi
Jung-Mo Ahn
Ganesh V. Raj

Last updated by author(s): Apr 19, 2022

Reporting Summary

Nature Research wishes to improve the reproducibility of the work that we publish. This form provides structure for consistency and transparency in reporting. For further information on Nature Research policies, see our Editorial Policies and the Editorial Policy Checklist.

Statistics

For all statistical analyses, confirm that the following items are present in the figure legend, table legend, main text, or Methods section.

n/a	Confirmed	
☐	☒	The exact sample size (*n*) for each experimental group/condition, given as a discrete number and unit of measurement
☐	☒	A statement on whether measurements were taken from distinct samples or whether the same sample was measured repeatedly
☐	☒	The statistical test(s) used AND whether they are one- or two-sided<br>*Only common tests should be described solely by name; describe more complex techniques in the Methods section.*
☒	☐	A description of all covariates tested
☐	☒	A description of any assumptions or corrections, such as tests of normality and adjustment for multiple comparisons
☐	☒	A full description of the statistical parameters including central tendency (e.g. means) or other basic estimates (e.g. regression coefficient) AND variation (e.g. standard deviation) or associated estimates of uncertainty (e.g. confidence intervals)
☐	☒	For null hypothesis testing, the test statistic (e.g. *F*, *t*, *r*) with confidence intervals, effect sizes, degrees of freedom and *P* value noted<br>*Give P values as exact values whenever suitable.*
☒	☐	For Bayesian analysis, information on the choice of priors and Markov chain Monte Carlo settings
☒	☐	For hierarchical and complex designs, identification of the appropriate level for tests and full reporting of outcomes
☒	☐	Estimates of effect sizes (e.g. Cohen's *d*, Pearson's *r*), indicating how they were calculated

Our web collection on statistics for biologists contains articles on many of the points above.

Software and code

Policy information about availability of computer code

Data collection	Gen 5 (version 3.09, BioTek) was used to collect live cell imaging, luminescence and fluorescence data from microplates.<br>ZEN (2.3 SP1, black, Carl Zeiss) was used to collect confocal and AiryScan data.<br>NIS Elements (4.13.04, Build 925, Nikon) was used to collect immunohistochemical staining data.<br>CFX Manager 3.1 (Bio-Rad) was used to collect qPCR data.<br>Image Lab 5.2.1 (Bio-Rad) was used to collect Western Blotting data.<br>ELISPOT images were captured by the ImmunoSpotÒ software, part of BioSpot 5.1.36 Professional (Cellular Technology Ltd.)
Data analysis	Gen 5 (version 3.09, BioTek) was used to analyze live cell imaging, luminescence and fluorescence data from microplates.<br>ZEN (2.3 SP1, black, Carl Zeiss) was used to analyze confocal and AiryScan data.<br>CFX Manager 3.1 (Bio-Rad) was used to analyze qPCR data.<br>Image Lab 5.2.1 (Bio-Rad) was used to analyze Western Blotting data.<br>CTL ImmunoSpot software (Biospot 5.1.36, Cellular Technology) was used to analyze ELISpot data.<br>Graphpad Prism (versions 8 and 9) was used for statistical analyses.<br>mRNA-seq data were analyzed by RNASeq Analysis Workflow (v.0.4.2 and v.0.5.15, https://git.biohpc.swmed.edu/BICF/Astrocyte/rnaseq) developed by Bioinformatics Core Facility of UTSW.<br>Tcga_shiny (version1.0.3, https://git.biohpc.swmed.edu/BICF/Astrocyte/tcga_shiny) developed by Bioinformatics Core Facility of UTSW was used for the TCGA analysis.<br>AutoDock Tools 1.5.6 (ADT; The Scripps Research Institute, La Jolla, CA, USA, RRID:SCR_012746) was used to create input PDBQT files of a protein and a ligand.<br>A predicted binding mode was visualized using Maestro (version 9.1, Schrödinger, LLC, New York, NY, USA).<br>MAGeCK (version 0.5.8) was used to analysis CRISPR screen data.<br>FACS data were analyzed by the FlowJo® software (version 10.8.1) (BD).<br>Gene ontology analysis were performed using the Database for Annotation, Visualization and Integrated Discovery (DAVID) bioinformatics resource v6.8 (https://david.ncifcrf.gov/tools.jsp).

The Ven diagram analysis was done using BioVenn (https://www.biovenn.nl/index.php).
Phoenix WinNonLin version 8.01 (Certara/Pharsight, Sunnyvale, CA) was used for LC-MS/MS analysis.
Scaffold DIA (v3.1.0; Proteome Software) was used for all DIA-MS data processing.

For manuscripts utilizing custom algorithms or software that are central to the research but not yet described in published literature, software must be made available to editors and reviewers. We strongly encourage code deposition in a community repository (e.g. GitHub). See the Nature Research guidelines for submitting code & software for further information.

Data

Policy information about availability of data

All manuscripts must include a data availability statement. This statement should provide the following information, where applicable:

- Accession codes, unique identifiers, or web links for publicly available datasets
- A list of figures that have associated raw data
- A description of any restrictions on data availability

mRNA-seq data are available from NCBI GEO under accession # GSE168800. Proteomic data are available from ProteomeXchange accession # PXD032693 and MassIVE accession #MSV000089091.

Field-specific reporting

Please select the one below that is the best fit for your research. If you are not sure, read the appropriate sections before making your selection.

☒ Life sciences ☐ Behavioural & social sciences ☐ Ecological, evolutionary & environmental sciences

For a reference copy of the document with all sections, see nature.com/documents/nr-reporting-summary-flat.pdf

Life sciences study design

All studies must disclose on these points even when the disclosure is negative.

Sample size	No statistical method was used to predetermine sample size. For in vivo experiments, we used at least 4 mice per group which is sufficient to detect meaningful biological difference. For in vitro experiments, unless otherwise stated, n=2 was chosen as the minimal number of replicates per experiment that would allow for adequate analysis to draw meaningful conclusions. We determined this to be sufficient based on the low observed variability between samples from in vitro experiments.
Data exclusions	Animals were excluded if the implanted tumors did not take.
Replication	For electron microscopy (Fig. 3g-h), confocal microscopy studies (Figs. 3i-j, 5g-h) and immunofluorescence (Extended Data Figs. 6a, 6d) where representative images were used, at least two independent experiments were performed with similar results. Tissue microarray staining (Fig. 6a) and mouse organ tissue staining (Extended Data Fig. 2a, 2d, 7d) were performed once. Immunohistochemical staining studies (Fig. 6i, Extended Data Fig. 3i) were performed in multiple samples. For blots/gels with representative images (Figs. 3m-o, 5k-l, 7a, 7j, 7m, 7q-r, 8n, Extended Data Figs. 3e-h, 3l, 5e, 5j-k,6e-f, 7c, 8b, 9f-g, 9j-k, 10a-b) each experiment was performed at least twice with similar results. The data shown in Extended Data Figs. 4d-h was performed once.
Randomization	in vitro studies were not randomized. All samples were analyzed equally without sub-sampling, therefore, there was no need for randomization. Mice were randomized for xenograft studies before initiation of treatment.
Blinding	Blinding was not used in any experiment in this study since it is not possible based on treatment and general conditions of the samples used.

Reporting for specific materials, systems and methods

We require information from authors about some types of materials, experimental systems and methods used in many studies. Here, indicate whether each material, system or method listed is relevant to your study. If you are not sure if a list item applies to your research, read the appropriate section before selecting a response.

Materials & experimental systems

n/a	Involved in the study	
☐	☒	Antibodies
☐	☒	Eukaryotic cell lines
☒	☐	Palaeontology and archaeology
☐	☒	Animals and other organisms
☐	☒	Human research participants
☒	☐	Clinical data
☒	☐	Dual use research of concern

Methods

n/a	Involved in the study	
☒	☐	ChIP-seq
☐	☒	Flow cytometry
☒	☐	MRI-based neuroimaging

Antibodies

Antibodies used

Antibodies used in this study are listed in Supplementary Table 2.
ARID1A, Santa Cruz, Cat #: sc-32761
ATF-4, Santa Cruz, Cat #: sc-390063
Calreticulin, Cell Signaling, Cat #: 12238
CHOP, Sigma, Cat #: HPA068416
DNAJC10, ABclonal, Cat #: A12260
eIF2a, Santa Cruz, Cat #: sc133132
ERalpha, Santa Cruz, Cat #: sc-8002
Histone H3, Abcam, Cat #: ab8898
IRE1a, Cell Signaling, Cat #: 3294
Ki67, GeneTex, Cat #: GTX16667
LAL, Santa Cruz, Cat #: sc-58374
LAL, Invitrogen, Cat #: PA5-84326
LAMP2, Santa Cruz, Cat #: SC-18822
LIMP2, Novus, Cat #: NB400-129SS
MANF, Bethyl, Cat #: A305-572A-T
Myc, Cell Signaling, Cat #: 2276
P3H2, ABclonal, Cat #: A8068
PDIA3, ABclonal, Cat #: A1085
PDIA5, ABclonal, Cat #: A14476
p-eIF2a, Cell Signaling, Cat #: 9721
PERK, Cell Signaling, Cat #: 5683
POGLUT2, Santa Cruz, Cat #: sc-390065
p-PERK, Abcam, Cat #: ab192591
SLC5A3, Proteintech, Cat #: 21628-1-AP
SOAT1, Santa Cruz, Cat #: sc-69836
TMEM208, Proteintech, Cat #: 23882-1-AP
Vinculin, Cell Signaling, Cat #: 13901
anti-moLy-6A/E (Sca-1)-PerCP, BioLegend, Cat #: 108121 (Clone D7)
anti-moCD3-APC-Cy7, BioLegend, Cat #: 100222 (Clone 17A2)
anti-moCD138-PE-Cy7, BioLegend, Cat #: 142514 (Clone 281-2)
anti-moIgD-FITC, BioLegend, Cat #: 405704 (Clone 11-26c.2a)
anti-mo?appaLight Chain-PE, ThermoFisher, Cat #: MKAPPA04 (Clone 187.1)
anti-moB220-BV421, BioLegend, Cat #: 103239 (Clone RA3-6B2)
Invitrogen™ eBioscience™ Fixable Viability Dye eFluor™ 506, ThermoFisher, Cat #: 50-246-097
anti-moIgM, SouthernBiotech, Cat #: 1020-01 (polyclonal)
anti-moIgG, SouthernBiotech, Cat #: 1030-01 (polyclonal)
anti-moIgM-biotin, SouthernBiotech, Cat #: 1020-08 (polyclonal)
anti-moIgG-biotin, SouthernBiotech, Cat #: 1030-08 (polyclonal)

Validation

All other antibodies used in this study were validated by manufacturers for that specific application (Western blotting, immunohistochemistry, immunefluorescence and flow cytometry). Relevant validating results can be found in the website of each manufacturer. Some antibodies are further validated in this study by CRSPR KO experiments (LAL, TMEM208, ARID1A, SOAT1 and SLC5A3) and overexpression experiment (Myc).

Eukaryotic cell lines

Policy information about cell lines

Cell line source(s)

Cell lines used in this study are listed in Supplementary Table 1 with detailed information about their culture conditions and source.
BT-20: ATCC
BT-549: ATCC
HCC1143: ATCC
HCC1187: ATCC
HCC1419: KCLB
HCC1569: KCLB
HCC1806: KCB
HCC1937: ATCC
HCC1954: ATCC
HCC202: ATCC
HCC2185: ATCC
HCC2688: UTSW Core
HCC38: ATCC
HCC70: BCRJ
MCF-7: ATCC
MCF-7/TamR: UT ObGyn Core
MDA-MB-157: ATCC
MDA-MB-231: ATCC

MDA-MB-436: ATCC
MDA-MB-453: ATCC
MDA-MB-468: ATCC
SKBR3: ATCC
SUM-159: UTSW Core
UACC812: ATCC
ZR-75: ATCC
A172: ATCC
Hs683: ATCC
NHAP-MP: UTSW Core
U251: UTSW Core
U87MG: ATCC
AsPC-1: ATCC
BxPC-3: ATCC
MIA Paca-2: ATCC
PANC-1: ATCC
ES2: ATCC
OVCAR3: ATCC
OVCAR4: Sigma
SKOV3: ATCC
HMEC: ATCC
HEK293T: ATCC

Authentication

Authentication was performed by STR profiling in UTSW sequencing core facility.

Mycoplasma contamination

All cell lines tested and negative for mycoplasma contamination.

Commonly misidentified lines
(See ICLAC register)

None.

Animals and other organisms

Policy information about studies involving animals; ARRIVE guidelines recommended for reporting animal research

Laboratory animals

Strains used in this study:
C57BL6, Female mouse, 6-8 weeks age
BALBc, Female mouse, 6-8 weeks age
SCID mice, Female mouse, 6-8 weeks age
NSG, Female mouse, 6-8 weeks age

We were in compliance with the UT Health San Antonio humane end points determination policy in our animal studies. None of the mice in our study showed any signs of moribund distress or had signs of unalleviated pain or distress during the experimental duration. None of the mice in our studies died because of the tumor burden. The goal per our approved protocol is to monitor experimental animals and sacrifice mice before the pathologies that may develop before debilitation or when the tumor size reaches ~2000mm3 in diameter. The 2000mm3 size criteria was chosen as the earliest humane end-point at which we could get meaningful data from these animal studies: based on prior experience, the tumors would have to get considerably larger to meet IACUC moribund distress criteria. For these studies, we constantly evaluate the size of the tumors based on growth rates and plan for euthanasia when the tumor size reaches ~2000mm3 in diameter.

In 9 out of 12 experiments presented in this paper and performed at UT Health San Antonio, the mice were euthanized before the tumors reached 2000mm3. In each of these studies, the decision was made to perform euthanasia using isoflurane inhalation followed by cervical dislocation close to the time when then tumors were reaching the target size. Although euthanasia scheduling typically took 1-4 days, since the rate of growth of these tumors was slow, we did not exceed our targeted goal of 2000mm3 in any of these mice.

However, in 3 PDX tumor studies (Fig 2k, 2o, 6k), some of the mice in the vehicle treated controls exceeded our goal of 2000mm3 size, due to unexpected, unpredictable and rapid growth rate of their tumors. In each case, when the tumor volumes were >2000mm3, we made the determination that the mice had reached the endpoint criteria and scheduled euthanasia. However, since euthanasia scheduling typically took 1-4 days, inevitably tumors continued to grow rapidly, reaching larger sizes at the time of sacrifice. In each case, we followed the humane endpoint guidelines as per IACUC policy. All the mice were active and none of these mice showed any signs of moribund distress and weight loss during the entire experimental duration. Thus, while we exceeded the goal of 2000mm3, we were in compliance with the UT Health San Antonio humane end points determination policy for each of these mice.

Wild animals

No wild animals were used in the study.

Field-collected samples

No field collected samples were used in the study.

Ethics oversight

Animal protocol 2016-101380 approved by IACUC office in UT Southwestern Medical Center. Animal protocol 20170183AR approved by IACUC office in UTHSCSA.

Note that full information on the approval of the study protocol must also be provided in the manuscript.

Human research participants

Policy information about studies involving human research participants

Population characteristics	Inclusion criteria included women with prior histologic confirmation of TNBC who were undergoing surgical extirpation or biopsy of their primary tumor. Prior treatment with chemotherapy and/or radiation was allowed. Exclusion criteria included concurrent or prior diagnosis of other malignancies or prior evidence of ER-alpha+ or HER2+ BC. All cases were reviewed by UTSW Tissue Repository in advance and patients were consented for their tissue to be used for laboratory research.
Recruitment	De-identified patient tumors were obtained from UTSW Tissue Repository after institutional review board approval (STU-032011-187).All cases were reviewed by UTSW Tissue Repository in advance and patients were consented for their tissue to be used for laboratory research. Only de-identified information was shared with the laboratory. None of the laboratory personnel had access to additional patient information.
Ethics oversight	De-identified patient tumors were obtained from UTSW Tissue Repository after institutional review board approval (STU-032011-187).

Note that full information on the approval of the study protocol must also be provided in the manuscript.

Flow Cytometry

Plots

Confirm that:

☒ The axis labels state the marker and fluorochrome used (e.g. CD4-FITC).

☒ The axis scales are clearly visible. Include numbers along axes only for bottom left plot of group (a 'group' is an analysis of identical markers).

☒ All plots are contour plots with outliers or pseudocolor plots.

☒ A numerical value for number of cells or percentage (with statistics) is provided.

Methodology

Sample preparation	Single cell suspensions were flushed from tibia and fibula using sterile DPBS using a 10 ml syringe and 30 G needle. Red blood cells were removed by incubation with ACK lysis buffer (Lonzo) for 2 min. Bone marrow cells (2x106) were first stained in Hank's Buffered Salt Solution plus 0.1% BSA (HBSS-BSA) for 20 m with fixable viability dye (FVD, eFluor™ 506, ThermoFisher, Cat. #50-246-097) and fluorophore-labeled mAbs to surface markers, including CD3 (APC-Cy7, Clone 17A2, BioLegend, Cat. # 100221,), IgD (FITC, Clone 11-26c.2a, BioLegend, Cat. #405704), CD138 (PE-Cy7, Clone 281-2, BioLegend, Cat. #142514), SCA-1 (PerCP, Clone D7, BioLegend, Cat. # 108121), B220 (BV421, Clone RA3-6B2, BioLegend, Cat. #103239), in the presence of mAb Clone 2.4G2, which blocks FcgII and FcgIII receptors. After washing, cells were then fixed and permeablized by incubation for 1 h in 250 ml BD Cytofix/Cytoperm™ buffer (BD Fixation/Permeablization Kit, Cat. # 554714) at 4°C. After washing twice with the BD Perm/Wash™ buffer, cells were counted again and 106 cells were resuspended in 100 ul of BD Cytofix/CytopermÔ buffer for intracellular staining with anti-Igkappa mAb (PE, ThermoFisher, Clone 187.1, Cat. # MKAPPA04) for 30 m. After washing with BD Perm/Wash™ buffer, cells were analyzed by LSRII (BD). FACS data were analyzed by the FlowJo® software (version 10.8.1, BD).
Instrument	LSRII (BD)
Software	FlowJo® software (BD) version 10.8.1
Cell population abundance	In Extended Data Figure 2e, the numbers within each plot indicate the proportions (relative abundance) of indicated cells or cell subsets within the cell populations in the previous step. The frequency of plasma cells was calculated by multiplying all sequential proportions. No sorting to purity was necessary for such calculations.
Gating strategy	The gating strategy is depicted in Extended Data Figure 2e and as follows:<br>All cells -> Lymphocytes (first plot in Supplementary Figure S2E) -> Single cells (second plot) -> Live cells (third plot) -> IgD- (fourth plot) -> Sca1+CD138hi (fifth plot) -> B220loIgkappa+ (sixth and last plot), with the gates within each plot depicting the boundary of indicated cells or cell subsets.

☒ Tick this box to confirm that a figure exemplifying the gating strategy is provided in the Supplementary Information.
