# Supplementary figures and images for: Targeting LIPA independent of its lipase activity is a therapeutic strategy in solid tumors via induction of endoplasmic reticulum stress
Source: Nat Cancer. 2022 Jun 2;3(7):866–84. doi: 10.1038/s43018-022-00389-8 (PMC9325671; doi:10.1038/s43018-022-00389-8)

Source data for Fig. 3

Fig. 3m

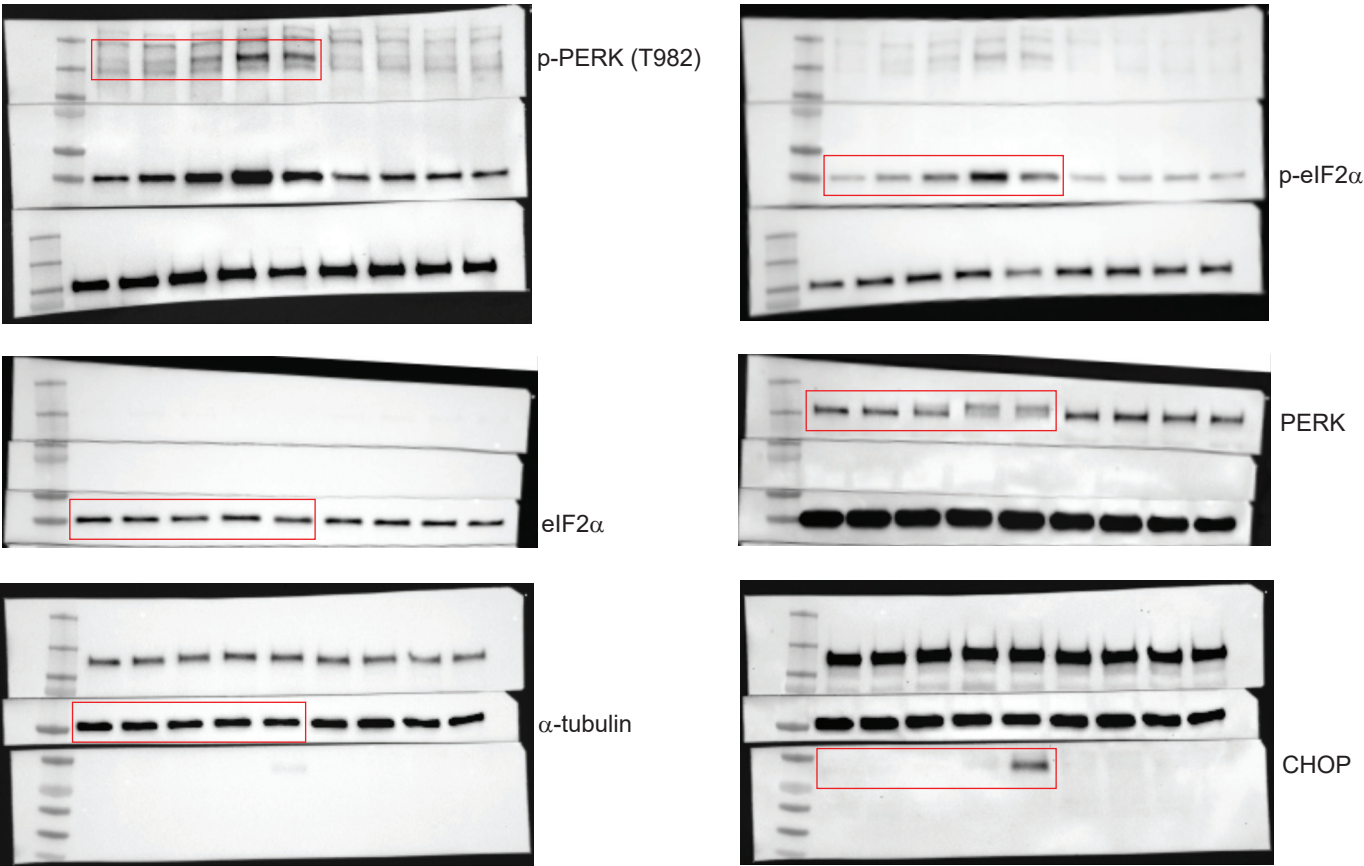

Fig. 3n

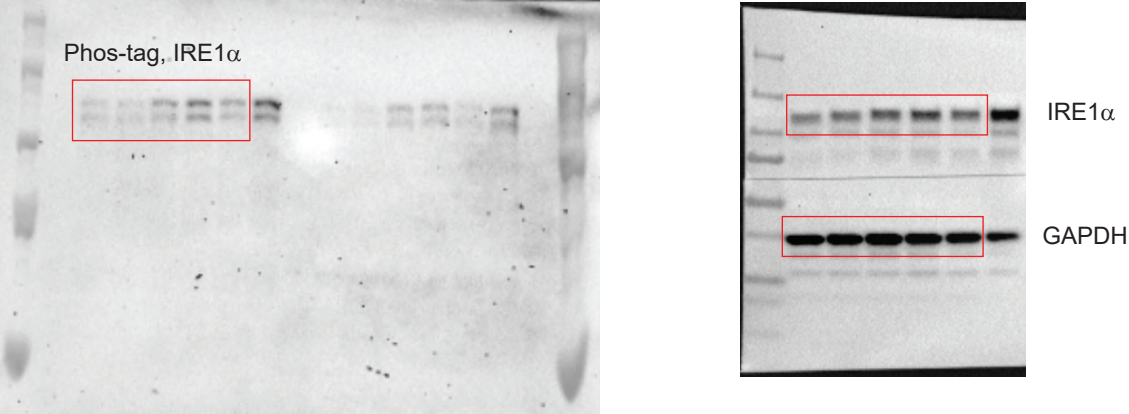

Fig. 3o

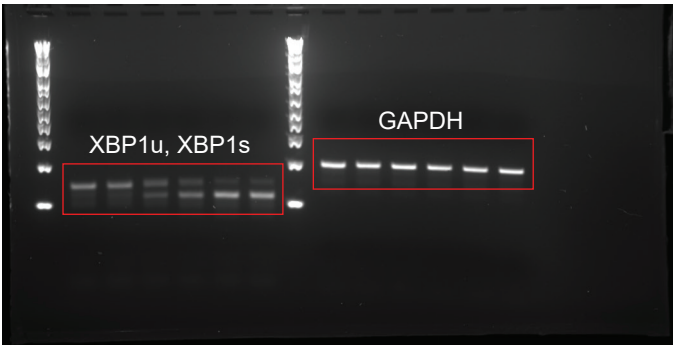

Supplement: Supplementary file 10 — Uncropped blots and gels. [file 43018_2022_389_MOESM10_ESM.pdf]

Source data for Fig. 5

Fig. 5k

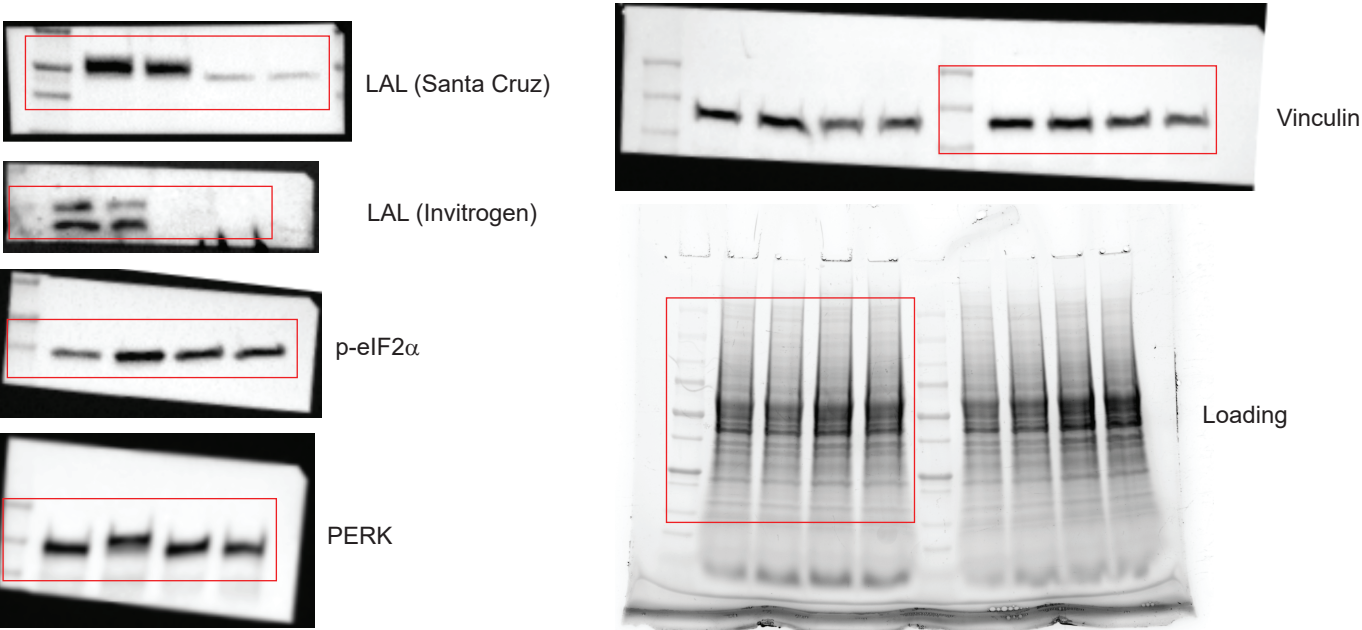

Fig. 5l

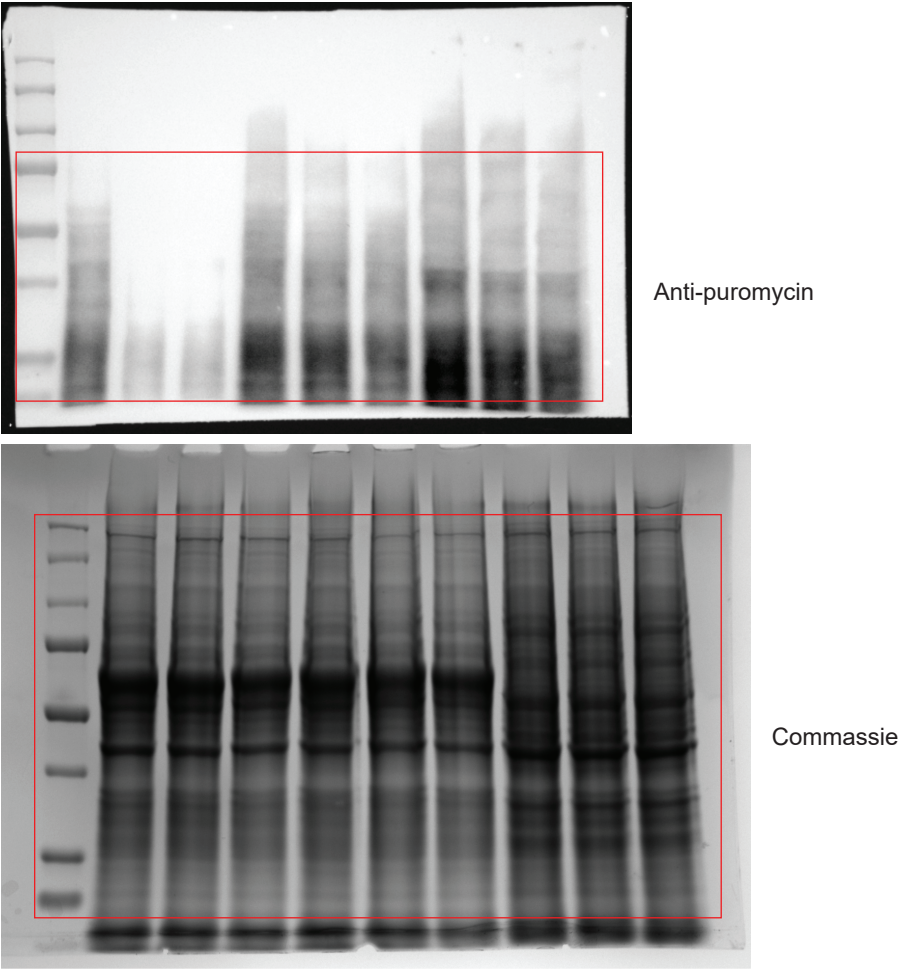

Supplement: Supplementary file 13 — Uncropped blots and gels. [file 43018_2022_389_MOESM13_ESM.pdf]

Source data for Fig. 6

Fig. 6c

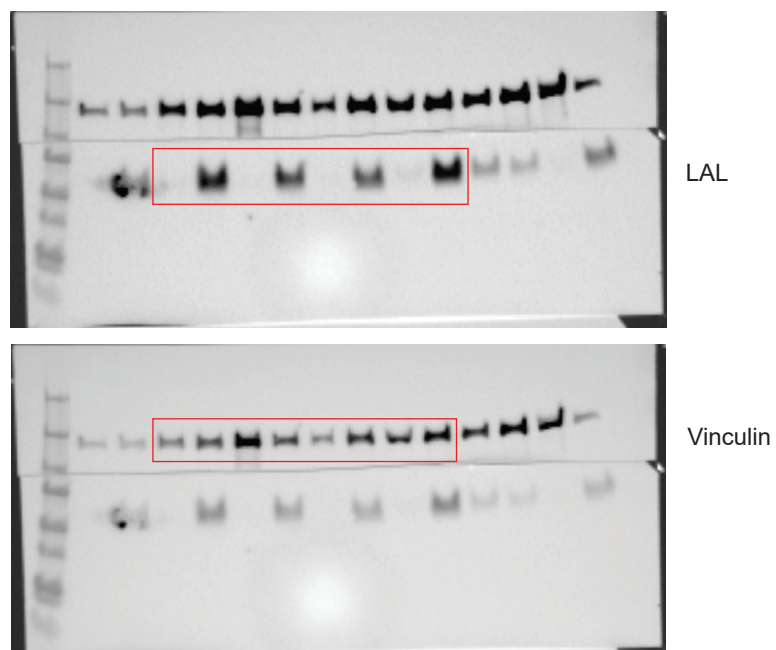

Supplement: Supplementary file 15 — Uncropped blots and gels. [file 43018_2022_389_MOESM15_ESM.pdf]

Fig. 7a

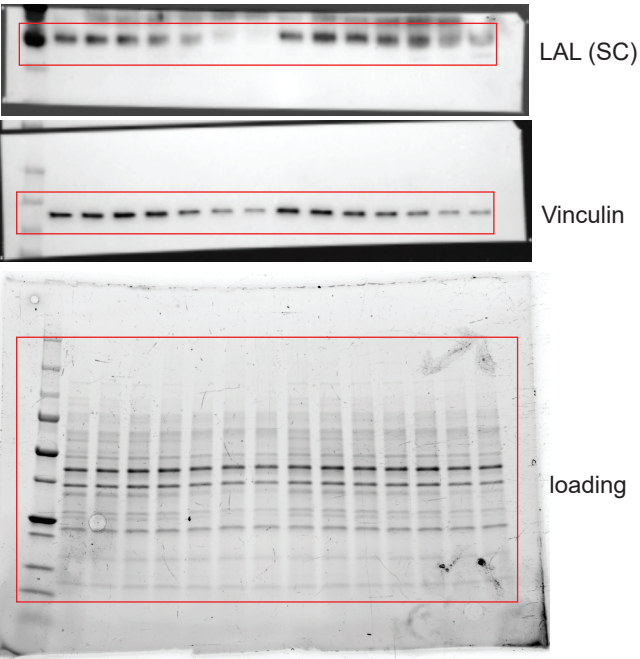

Fig. 7j

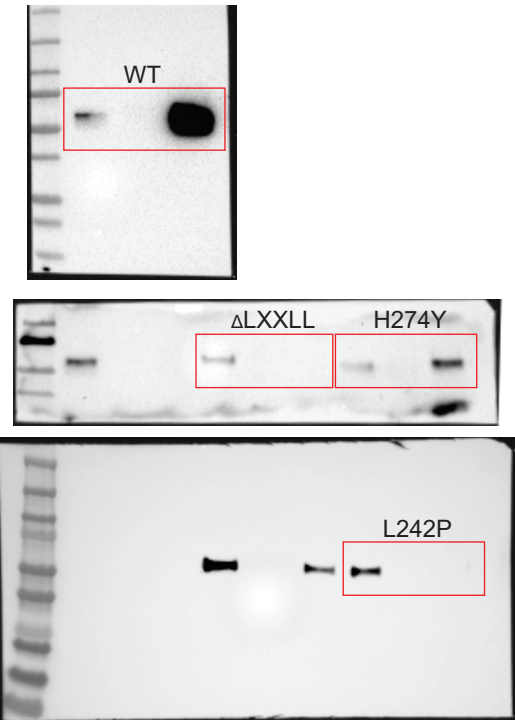

Fig. 7l

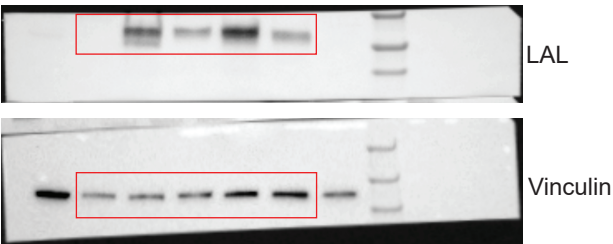

Fig. 7m

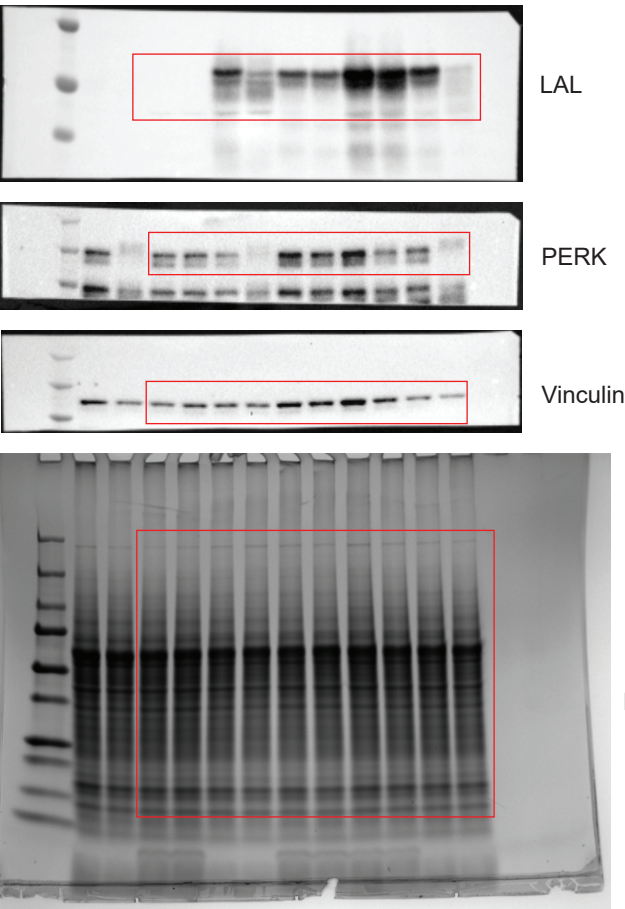

Fig. 7q

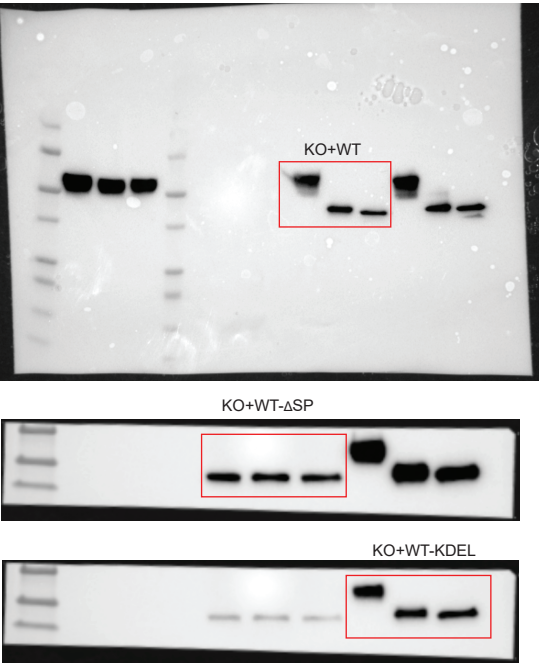

Source data for Fig. 7

Fig. 7r

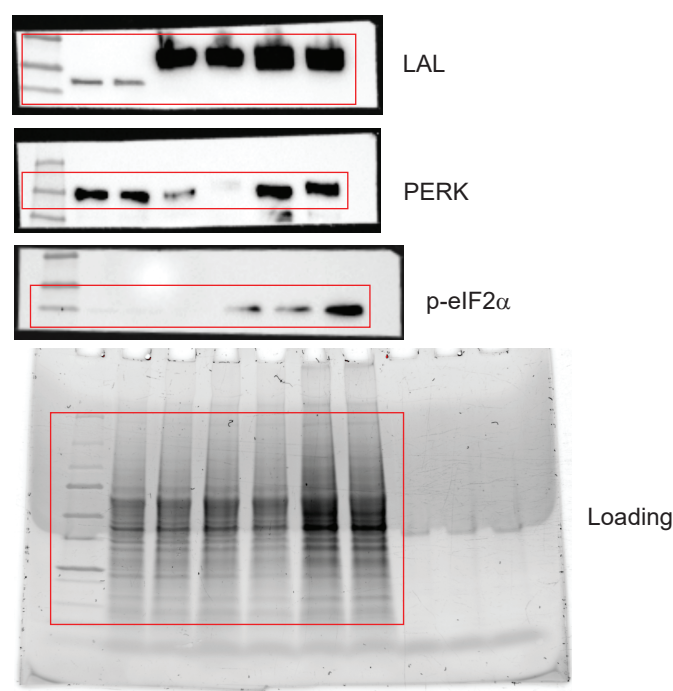

Supplement: Supplementary file 17 — Uncropped blots and gels. [file 43018_2022_389_MOESM17_ESM.pdf]

Fig. 8o

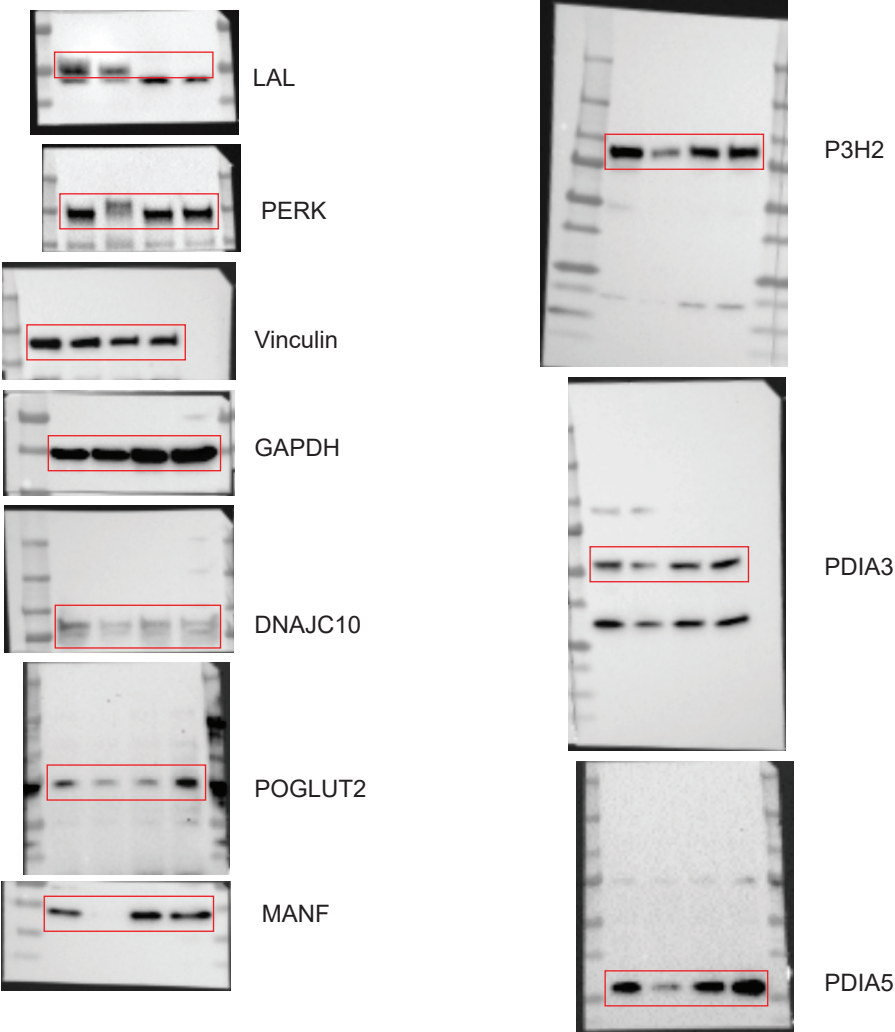

Supplement: Supplementary file 19 — Uncropped blots and gels. [file 43018_2022_389_MOESM19_ESM.pdf]

Source data for Extended Data Fig. 4

Fig. 4d

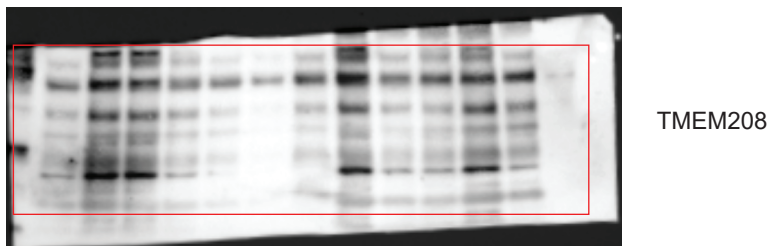

Fig. 4e

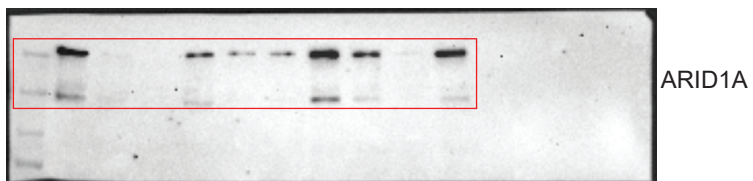

Fig. 4f

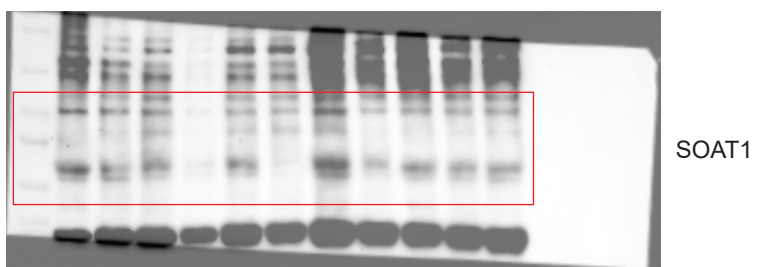

Fig. 4g

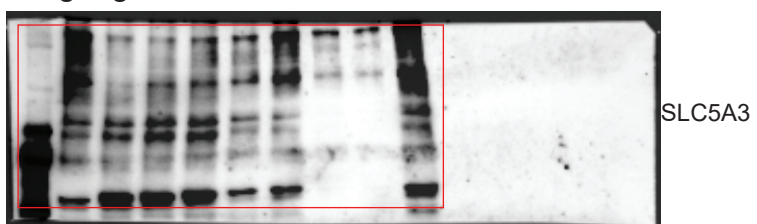

Fig. 4h

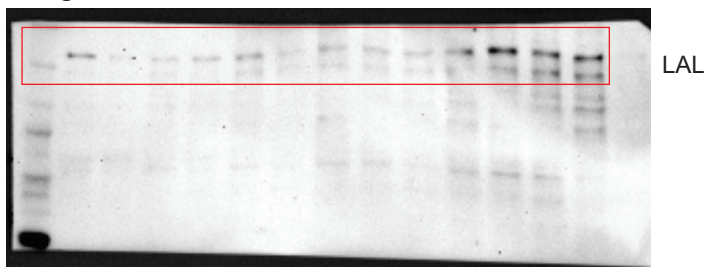

Supplement: Supplementary file 25 — Uncropped blots. [file 43018_2022_389_MOESM25_ESM.pdf]

Fig. 5e

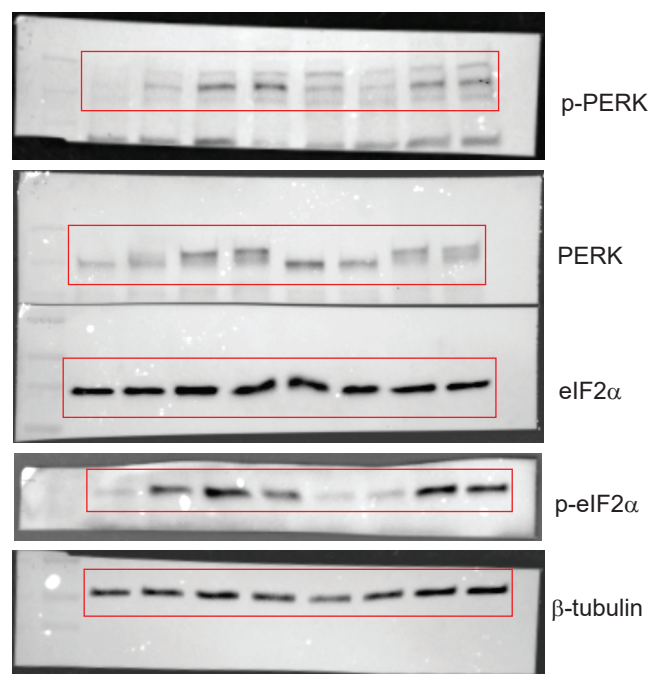

Supplement: Supplementary file 27 — Uncropped blots. [file 43018_2022_389_MOESM27_ESM.pdf]

Fig. 6e

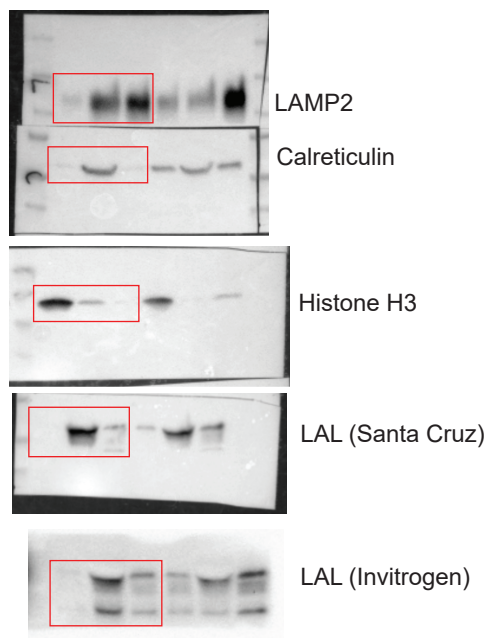

Fig. 6f

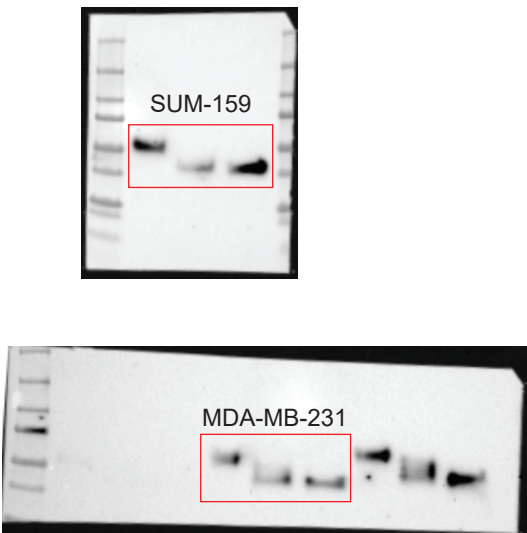

Supplement: Supplementary file 28 — Uncropped blots. [file 43018_2022_389_MOESM28_ESM.pdf]

Source data for Extended Data Fig. 7

Fig. 7c

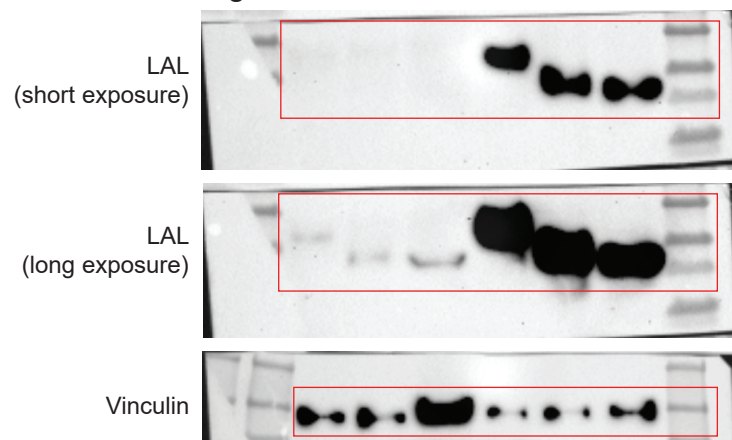

Supplement: Supplementary file 29 — Uncropped blots. [file 43018_2022_389_MOESM29_ESM.pdf]

Source data for Extended Data Fig. 8

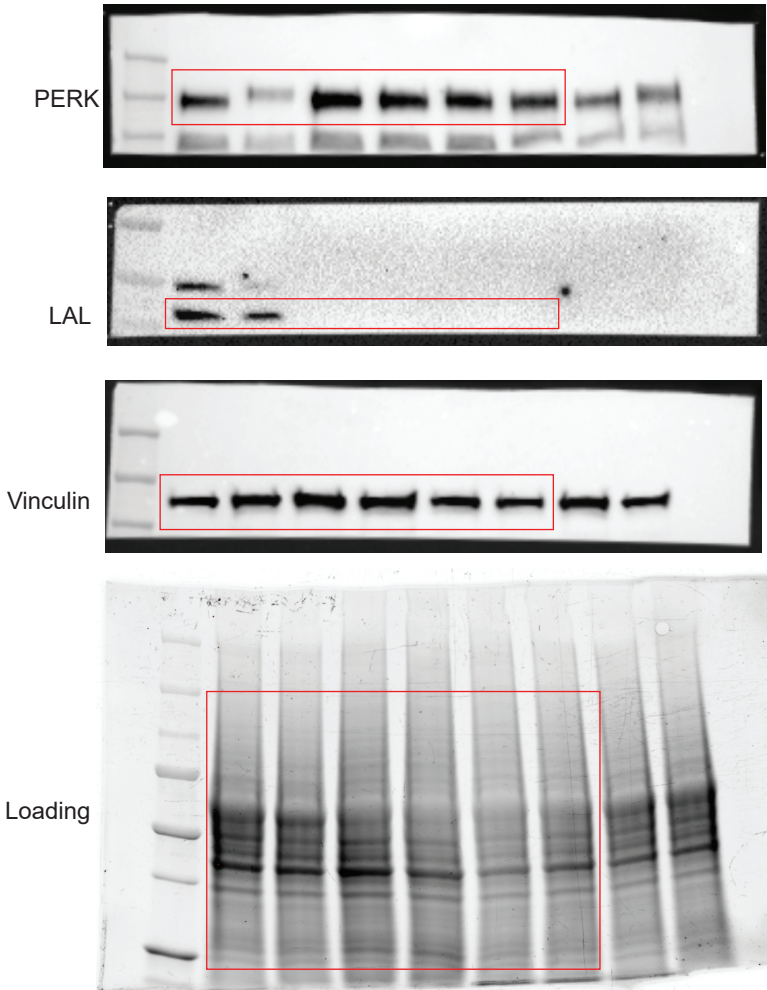

Supplement: Supplementary file 31 — Uncropped blots and gels. [file 43018_2022_389_MOESM31_ESM.pdf]

Fig. 9f

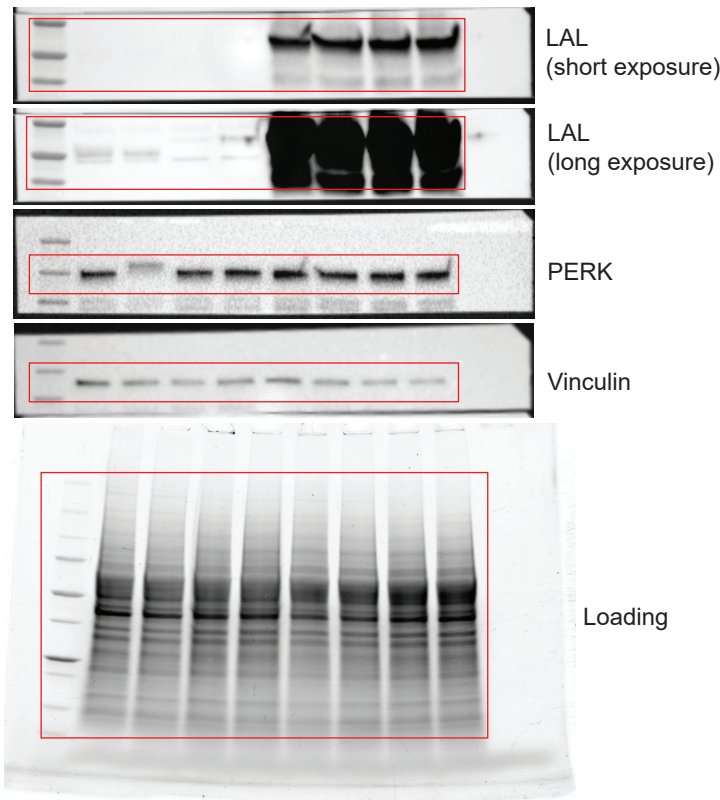

Fig. 9j

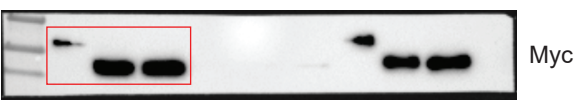

Fig. 9k

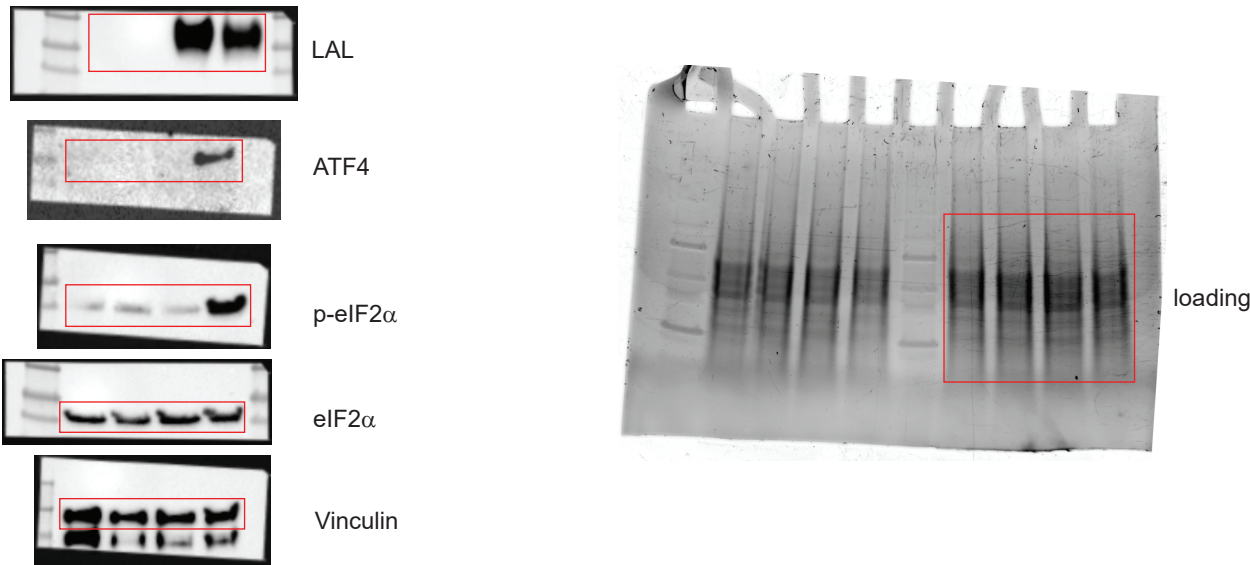

Supplement: Supplementary file 33 — Uncropped blots and gels. [file 43018_2022_389_MOESM33_ESM.pdf]

Fig. 10a

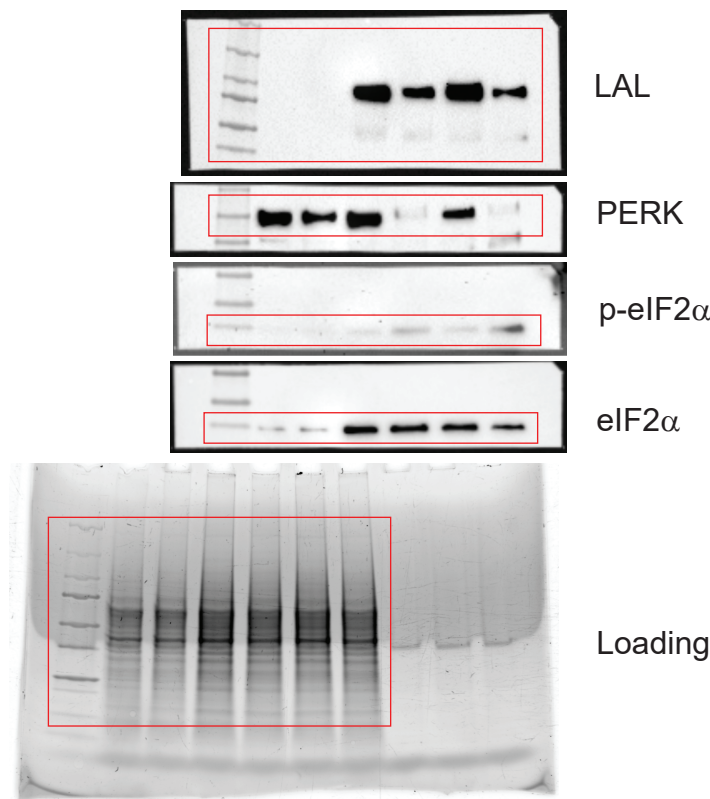

Fig. 10b

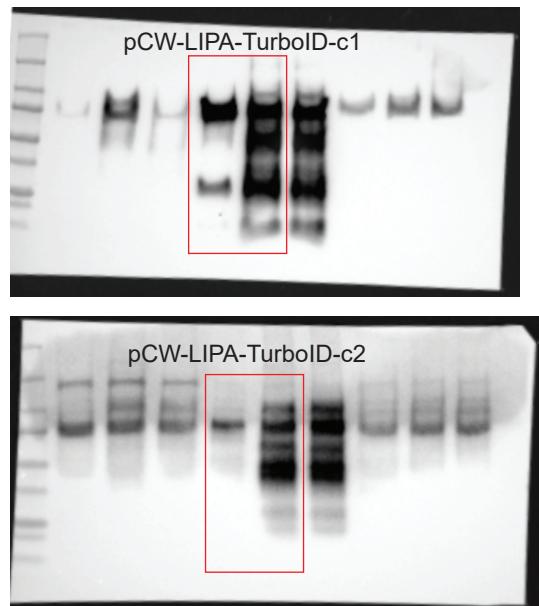

Supplement: Supplementary file 35 — Uncropped blots and gels. [file 43018_2022_389_MOESM35_ESM.pdf]
