# Supplementary material for: Targeting LIPA independent of its lipase activity is a therapeutic strategy in solid tumors via induction of endoplasmic reticulum stress
Source: Nat Cancer. 2022 Jun 2;3(7):866–84. doi: 10.1038/s43018-022-00389-8 (PMC9325671; doi:10.1038/s43018-022-00389-8)

Source data for Extended Data Fig. 3e

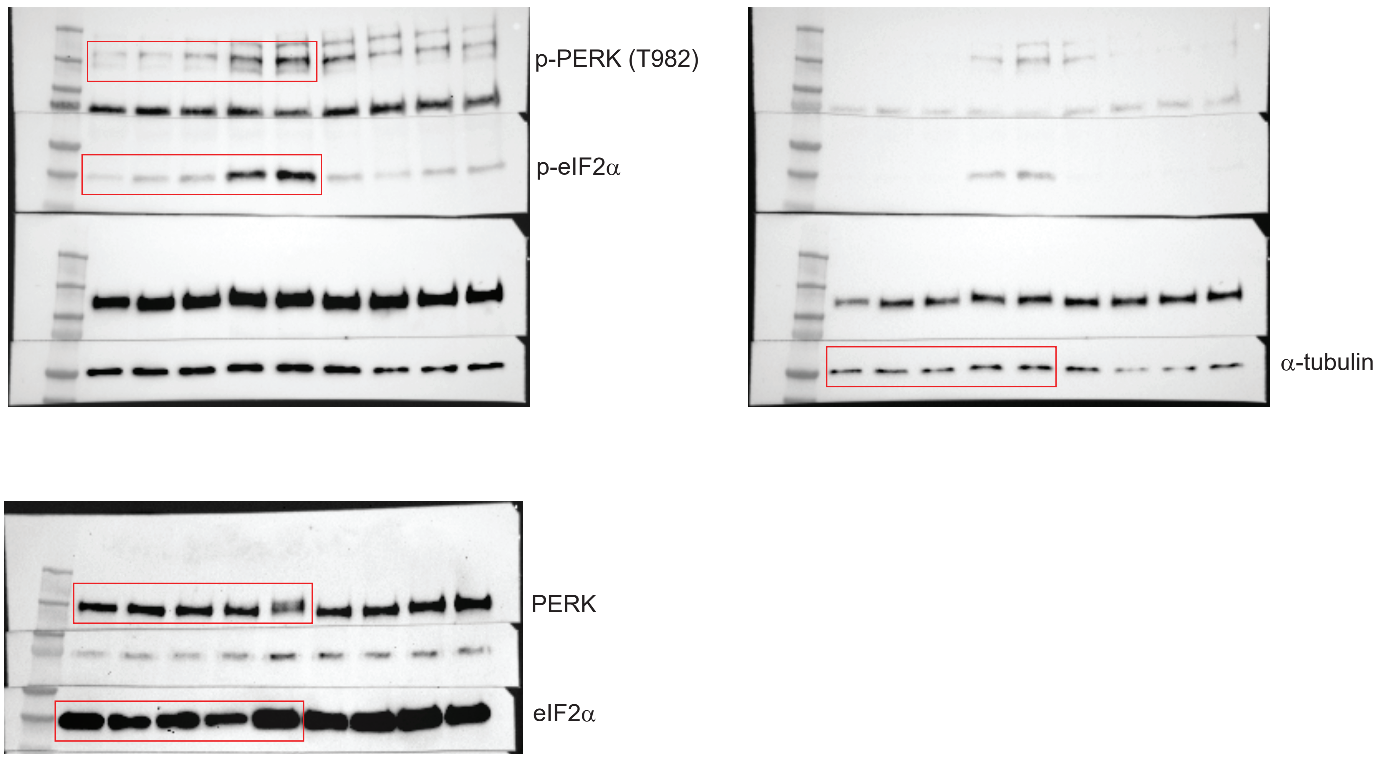

Source data for Extended Data Fig. 3f

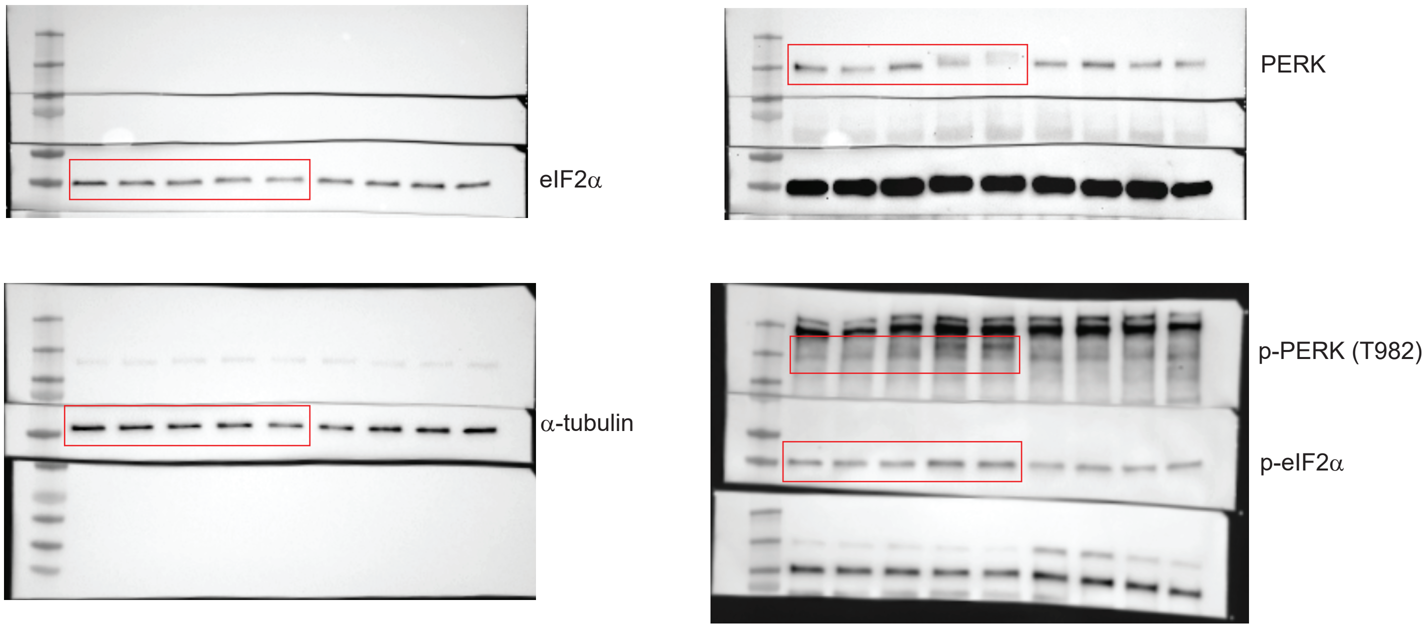

Source data for Extended Data Fig. 3g

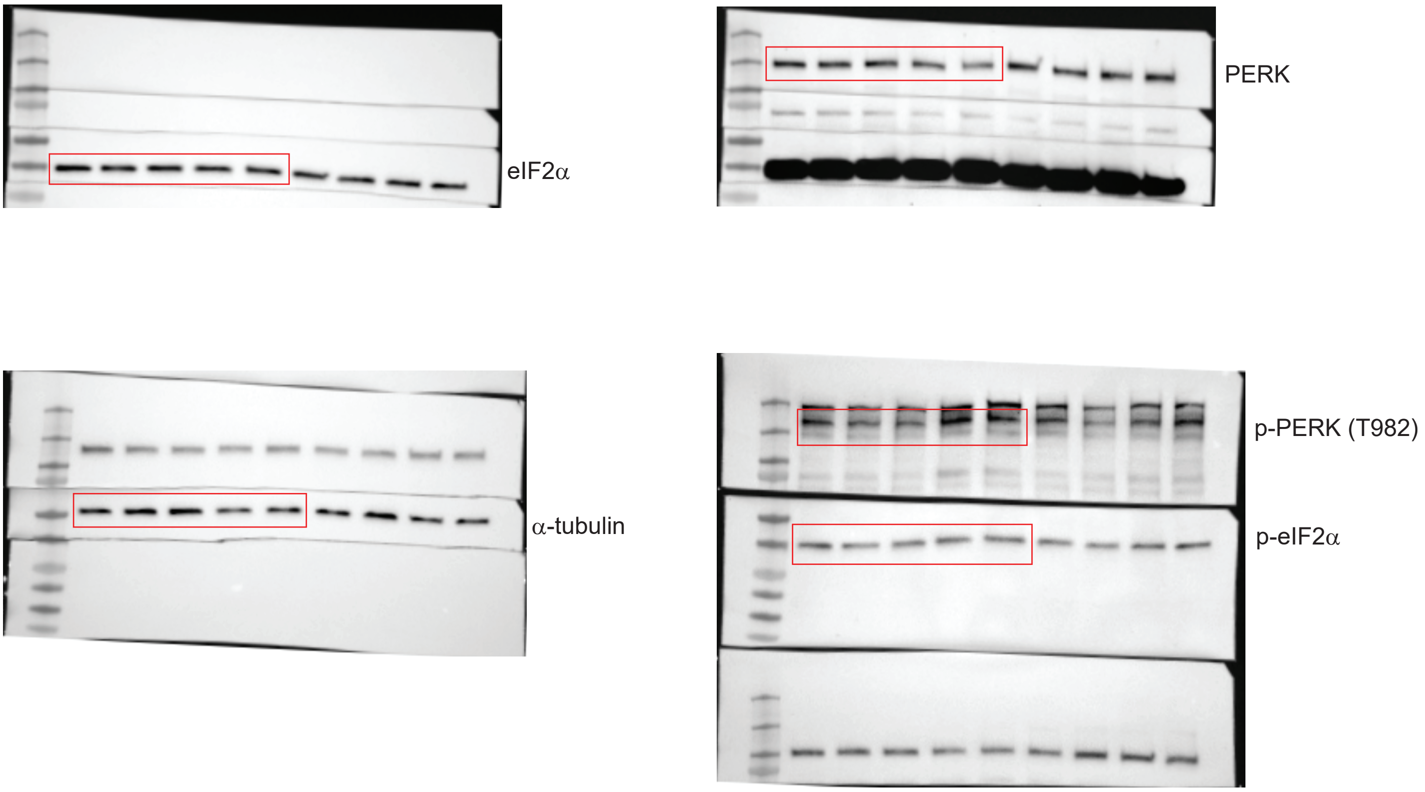

Source data for Extended Data Fig. 3h

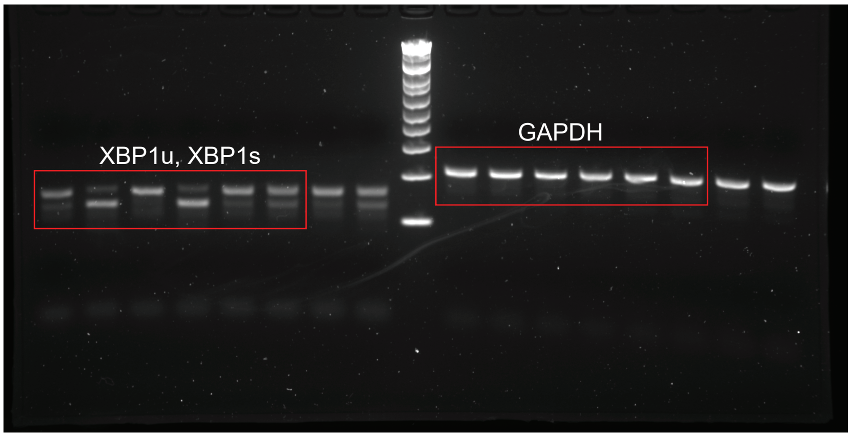

Source data for Extended Data Fig. 3i

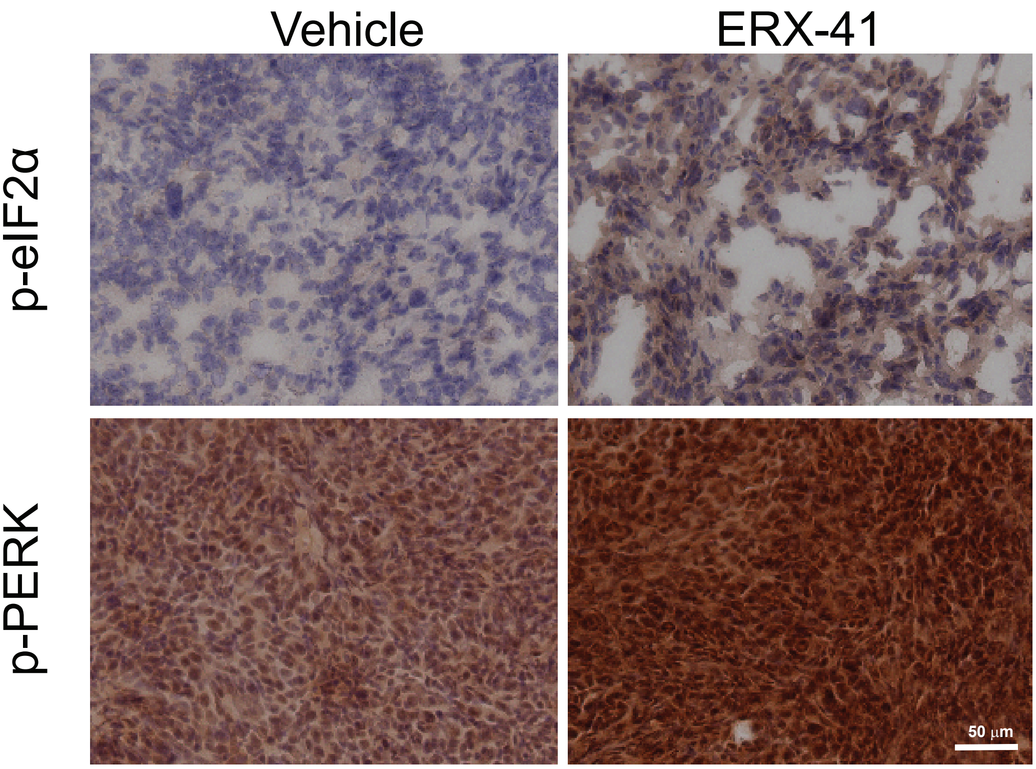

Source data for Extended Data Fig. 3l

MDA-MB-231

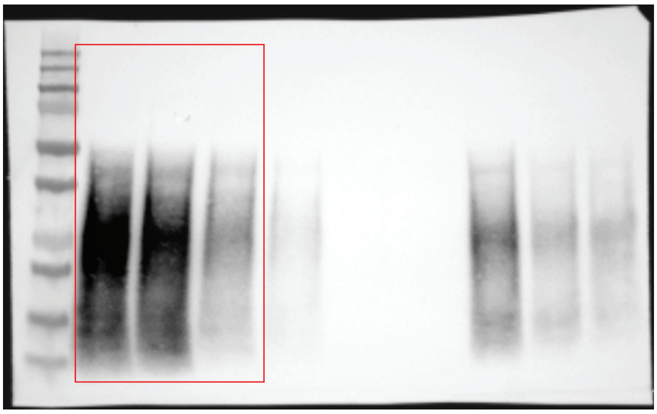

BT-549

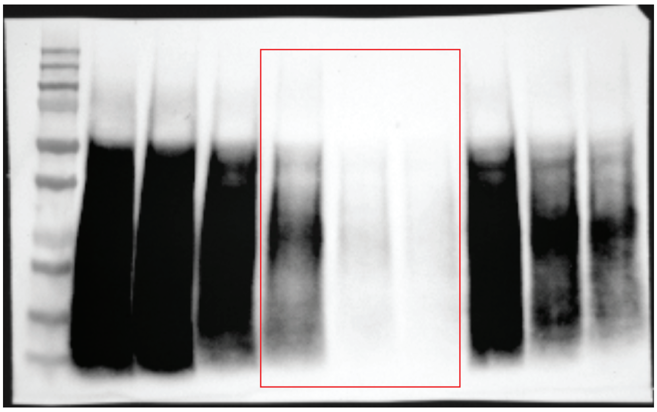

HMEC

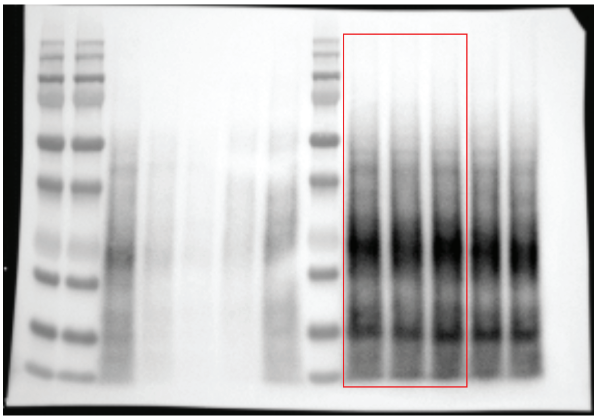

Supplement: Supplementary file 23 — Uncropped blots, gels and images. [file 43018_2022_389_MOESM23_ESM.pdf]
